# Exosomal small non-coding RNA profiling and the role of PIWI-interacting RNA pathway genes in Lumpy skin disease virus-infected bovines

**DOI:** 10.5713/ab.25.0217

**Published:** 2025-06-04

**Authors:** Anh Duc Truong, Ha Thi Thanh Tran, Thi Hoai Phan, Thi Hao Vu, Nhu Thi Chu, Hieu Minh Nguyen, Linh Phuong Nguyen, Lanh Phan, Chaeeun Kim, Hoang Vu Dang, Yeong Ho Hong

**Affiliations:** 1 Department of Biochemistry and Immunology, National Institute of Veterinary Research, Hanoi, Vietnam; 2 Department of Animal Science and Technology, Chung-Ang University, Anseong, Korea

**Keywords:** Bovine, Lumpy Skin Disease, Non-coding RNA, PIWI-interacting RNA, RNS-seq

## Abstract

**Objective:**

Lumpy skin disease (LSD) is a reemerging viral disease impacting cattle and buffaloes, posing substantial economic risks. However, the expression profile of non-coding RNAs (ncRNAs) in LSD virus (LSDV)-infected bovines has yet to be investigated. In this study, we employed small RNA sequencing (RNA-seq) to assess the expression of various ncRNAs in serum-derived exosomes from LSDV-infected bovines. We particularly focused on the bio-functional activity of PIWI-interacting RNAs (piRNAs).

**Methods:**

Cattle were infected with a 10^6.5^ TCID_50_/mL LSDV Vietnam/HaTinh/CX01 (HT10) strain and ncRNAs expression in the serum of infected cattle was analyzed small RNA-seq.

**Results:**

We identified 426 significantly differentially expressed (DE) piRNAs in serum-derived exosomes from LSDV-infected bovines compared to control groups, with 80 piRNAs being upregulated and 346 piRNA genes downregulated. Pathway analysis of DE piRNAs revealed their involvement in metabolism, cell signaling, and immune response pathways. Additionally, we identified a total of 35,170 tRNAs, 917 snoRNAs, 1,578 snRNAs, 17 Y-RNAs, five small cytoplasmic RNAs (scRNAs), ten vault RNAs, 248 sRNAs, 1,064 piRNAs, and 1,011 miRNAs (not shown in this study) expressed in serum-derived exosomes from LSDV-infected bovines. Among these, 15,649 DE tRNAs, 476 DE snoRNAs, 861 DE snRNAs, 11 DE Y-RNAs, three DE scRNAs, three DE vault RNAs, and 134 DE sRNAs were identified when compared to the control group.

**Conclusion:**

Our comprehensive analysis of small RNA-seq data revealed numerous DE ncRNAs in serum-derived exosomes from LSDV-infected bovines compared to controls. We propose that further elucidation and validation of the functions of these ncRNAs may be beneficial for the diagnosis, treatment, and prognosis of LSDV in bovines.

## INTRODUCTION

Lumpy skin disease (LSD) is a resurging viral ailment affecting cattle and buffaloes, posing a significant economic burden [1]. Classified as a notifiable disease by the World Organization for Animal Health [1], LSD spreads through hematophagous vectors such as mosquitoes and flies, facilitating swift dissemination during conducive climates [1]. Significant losses have been incurred due to LSD outbreaks in the European Union and Asia, involving animal fatalities, reduced productivity, control measures expenses, and market disruptions [2]. Disease severity ranges from asymptomatic to lethal, influenced by virus virulence and breed susceptibility, generally resulting in a mortality rate below 10% and morbidity ranging from 0% to 90% [3]. Initially confined to southern and eastern Africa since its discovery in Zambia in 1929, LSD expanded beyond sub-Saharan Africa during the late 1980s, emerging in Egypt and Israel [3]. It is prevalent in numerous African nations and has spread to southeast Europe, the Middle East, and South Asia, with documented outbreaks in China, India, Bangladesh, and Nepal [4,5]. The first outbreak of LSD in Vietnam occurred in October 2020 [6]. In response to this, vaccination initiatives utilizing a live-attenuated LSD virus (LSDV) vaccine centered on the Neethling strain have played a pivotal role in disease containment in Vietnam and Southeast Europe [6]. Recently, several studies have been conducted on the epidemiology of LSDV outbreaks and the immune response of bovine to LSDV or vaccination [7–10]. Nevertheless, there is a dearth of the small RNA component and expression in bovines infected with LSDV, especially in serum-derived exosomes from LSDV-infected bovines.

Non-coding RNAs (ncRNAs) represent a broad category of functional RNA molecules lacking sequences coding for proteins [11,12]. These encompass tRNAs, rRNAs, small cytoplasmic RNAs (scRNAs), repeat-associated small interfering RNA (rasiRNA), snRNAs, snoRNAs, miRNAs, siRNAs, and PIWI-interacting RNAs (piRNAs) [12–14]. These small ncRNAs, which are 18 to 32 nucleotides in length, play crucial roles in various cellular processes. Moreover, their abundance and functions are currently being extensively investigated across different model organisms and diseases. PiRNAs, first discovered in mouse testes in 2006, are single-stranded RNAs ranging from 25 to 32 nucleotides in length [12–14]. These RNAs are predominantly present in germ cells and functionally rely on their interaction with PIWI proteins to form piRNA-induced silencing complexes (RISC). Research on the piRNA pathway has demonstrated its involvement in transcriptional and post-transcriptional gene silencing. This includes heterochromatinization through DNA methylation or histone modifications, in addition to the targeting of transposable elements and protein-coding gene transcripts through RISC activity [13,15].

Furthermore, piRNAs exhibit diverse functions beyond germline regulation, influencing tissue regeneration, tumor biology, and embryogenesis, as demonstrated by research in somatic and cancer cells [13,15]. Additionally, piRNAs, are a class of small ncRNA molecules that play crucial roles in gene regulation and transposon control in various organisms. piRNAs are increasingly recognized in bovine diseases as potential key regulators of immune responses and disease progression. piRNAs are involved in the innate immune response and defense against viral and bacterial infections [16,17]. It has been demonstrated that piRNAs can modulate host immune responses, promoting inflammation and activating immune cells to combat infectious agents such as bovine respiratory disease, mastitis, and reproductive disorders in cattle [18]. Consequently, comprehensive investigations into piRNA transcriptomes across different species are imperative for a more profound comprehension of their regulatory mechanisms and evolutionary dynamics. Furthermore, exosomes, typically ranging from 30 to 250 nanometers in diameter, play a significant role in animals’ immune responses to infectious diseases [19]. Exosomes carry diverse molecular constituents, such as proteins, lipids, RNA, DNA, and snRNA, which can profoundly modulate the immune system through various immune cells [10,20,21]. Exosomal sncRNAs are being increasingly recognized for their critical roles in a variety of diseases, including cancer, cardiovascular diseases, avian influenza, and neurodegenerative disorders. These roles are attributed to their ability to modulate the activity of immune cells and play an essential role in immune surveillance and inflammation [20,21].

In this study, we performed high-throughput next-generation sequencing (NGS) to profile various types of ncRNAs, particularly piRNAs, in exosomes extracted from the serum of cattle infected with LSDV and to elucidate the functions of piRNA pathway genes.

## MATERIALS AND METHODS

### Lumpy skin disease virus strain, animal experiment, and real-time polymerase chain reaction

The LSDV Vietnam/HaTinh/CX01 (HT10) strain, obtained from Ha Tinh Province, North Central Vietnam, underwent adaptation and cultivation in Madin-Darby bovine kidney (MDBK) cells (KCLB#10022, Seoul, Korea). MDBK cells were cultured at 37°C in a 5% CO_2_ environment, in a Dulbecco’s Modified Eagle’s Medium (DMEM, Invitrogen) supplemented with penicillin (100 IU/mL), streptomycin (100 mg/mL), fungizone (1 μg/mL from Sigma-Aldrich), and 5% heat-inactivated fetal bovine serum (FBS). Virus cultivation entailed freeze-thaw cycles, with the clarified supernatant subsequently transferred to new cultures for multiple passages until cytopathogenic effects (CPE) were evident. Changes in CPE appearance and viral titer indicated cell susceptibility. The LSDV titer was quantified through titration in 96-well tissue culture plates using Reed and Muench’s method, reaching 10^7^ TCID50/mL after six passages. The treatment (HT10) and control groups comprised three Vietnamese local yellow cattle (*Bos taurus*) individuals aged between four and five months housed at the National Institute of Veterinary Research, Vietnam. The treatment group was intravenously injected with the HT10 strain (10^6.5^ TCID50/ml on MDBK cells), and the control group was injected with PBS. Post-treatment, body temperature, and clinical manifestations were monitored daily, and specimens were collected for real-time polymerase chain reaction (PCR) analyses. A necropsy and additional analyses were performed on deceased animals. Real-time PCR was conducted employing viral p32 gene-specific primers on DNA extracted with the QIAamp DNA Mini Kit (Qiagen) in accordance with WHOA guidelines, utilizing positive and negative amplification controls on a QuantStudio 5 Real-Time PCR System (Thermo Fisher Scientific).

### Exosome purification and characterization

Exosomes were purified from serum samples of both healthy and LSDV-infected bovines using the Total Exosome Isolation Reagent (Invitrogen) in accordance with the manufacturer’s protocol. The exosomes’ size was determined using a nanoparticle analyzer (HORIBA SZ-100). To further analyze the exosomes, western blotting was performed on combined exosome samples using three known exosomal markers: CD9 (#13174; Cell Signaling Technology), CD63 (sc-5275; Santa Cruz Biotechnology), and CD81 (#56039; Cell Signaling Technology), following established procedures [10,22].

### Exosomal RNA isolation and small RNA sequencing

Exosomal RNA was individually extracted using the miRNeasy Serum/Plasma Kit (Qiagen) following the manufacturer’s protocol. At 21 days post-infection (dpi), three samples were selected from both the control and infected groups based on clinical evaluations, which confirmed that all LSDV-infected cattle at 21 dpi displayed clinical, digestive, or respiratory symptoms. These samples were utilized to generate a small RNA library employing the SMARTer smRNA-Seq Kit for Illumina (TAKARA Bio), following the manufacturer’s instructions. The quality and quantity of the isolated small RNA were assessed using an Agilent 2100 Bioanalyzer (Agilent Technologies). Small RNA sequencing was conducted on the Illumina platform (Illumina) by Macrogen.

### Sequencing data analysis

Firstly, the FastQC v0.11.7 (https://www.bioinformatics.babraham.ac.uk/projects/fastqc/) program was used to perform filtering and quality assessment of the raw sequences before analysis to ensure data integrity. Then, Cutadapt v4.4 (https://cutadapt.readthedocs.org/en/stable/) was utilized to remove adapter sequences, primers, poly-A tails, and other types of unwanted sequences from high-throughput sequencing reads. For each sample, final processed reads were sequentially aligned to a bovine reference genome (ARS-UCD1.2, RefSeq: GCF_002263795.3), miRBase v22.1 (http://www.mirbase.org/) and ncRNA database (RNAcentral release 22.0; https://rnacentral.org/) to classify known miRNAs and other types of RNA such as piRNA, tRNA, snRNA, snoRNA etc. Genome mapping was performed by Bowtie2 version v.2.5.2 (http://bowtie-bio.sourceforge.net/index.shtml) and STAR version v.2.6.0c using RSEM version v1.3.1 (http://deweylab.github.io/RSEM/). The Bowtie output was subsequently used for miRDeep2 v2.0.0.8 (https://www.mdc-berlin.de/content/mirdeep2-documentation) analysis using the genomic sequence. The RSEM program was utilized to align reads to transcript sequences via STAR, if required. Known/novel miRNA predicted by miRDeep2 as indicated in prior reports [10] and other smRNAs matching RNAcentral were aligned using Bowtie (target smRNA, <50 nt) and Bowtie2 (target smRNA, ≥50nt). Finally, reads exhibiting a 1U or 10A feature within the remaining sequences were identified as potential piRNA reads. Among these, piRNA reads with identical sequences were grouped and were considered piRNA tags. Subsequently, the length distribution of piRNA reads and tags (reads with identical nucleotide sequences considered as tags) in serum-derived exosomes from LSDV-infected cattle was analyzed, and the base frequency for each position from the 5′ to 3′ ends was determined. The predicted piRNA sequences were subsequently aligned to the bovine genome using bowtie2 (version 2.5.2) to determine their genomic composition.

### Differentially expressed genes, target gene prediction, and pathway analysis

Differentially expressed (DE) small RNAs were statistically analyzed based on fold changes and a precise edgeR comparison pair (empirical analysis of digital gene expression data in R). The criteria of a |fold-change|≥2 and an exactTest raw p-value<0.05 were employed to identify statistically significant results. The target genes of the DE piRNAs were predicted using various tools, including piRNAQuest V.2 (http://dibresources.jcbose.ac.in/zhumur/pirnaquest2/), Airbase (http://bigdata.ibp.ac.cn/piRBase/index.php), and NCBI BLAST with sequence coverage >70% and identity >80%. Additionally, the programs miRanda, RNAhybrid, and TargetScan were utilized to predict DE piRNA target genes. The overlapping genes identified by these three programs were designated as candidate target genes. To investigate the properties of these genes and their products, we conducted gene ontology (GO) (http://geneontology.org/) annotation and enrichment analysis across three categories: molecular function, cellular component, and biological process. Functional analysis of the predicted gene targets was performed using the Database for Annotation, Visualization, and Integrated Discovery (DAVID 2021, Dec. 2021) (https://david.ncifcrf.gov/summary.jsp#) for pathway analysis.

### Reverse transcription quantitative real-time polymerase chain reaction

Quantitative polymerase chain reaction (qPCR) was employed to validate the accuracy of small RNA sequencing. Five piRNAs were randomly chosen for validation, while U6 served as the reference gene. The primers for qPCR amplification are listed in Table 1. Total RNA was reverse transcribed into cDNA using the Mir-X miRNA First-Strand Synthesis Kit (Takara Bio). The Mir-X miRNA qRT-PCR TB Green kit (Takara Bio) was then utilized to quantify piRNA expression following the manufacturer’s instructions. The reverse transcription quantitative real-time polymerase chain reaction (RT-qPCR) reaction conditions were as follows: an initial step at 95°C for 15 minutes, followed by 40 cycles of 10 seconds at 95°C and 30 seconds at 60°C. Each sample was tested in three technical replicates. Finally, the relative expression levels of the piRNAs were calculated using the 2^−ΔΔCt^ method [23].

### Statistical analysis

Statistical analysis was conducted using SPSS software (version 25.0; IBM). Data is presented as mean±standard error of the mean (SEM). Comparisons between groups were conducted using a two-tailed Student’s t-test, with statistical significance established at p<0.05.

## RESULTS

### Sequence analysis of non-coding RNA in Lumpy skin disease virus-infected bovines

High-throughput NGS was carried out on serum-derived exosomes from both LSDV-infected and control bovines using the Illumina HiSeq platform. Raw NGS data were processed to yield clean reads by removing low-quality reads (Q-value<13), short read tags (<18 nt), and adaptor-ligated contaminants. Post-cleaning, the raw data comprised 17.952.058, 42.157.230, 46.774.533 total reads in three control bovines and 43.057.103, 40.705.089, 41.778.682 total reads in three LSDV-infected bovines, respectively. From these total reads, we obtained unique 2.876.226, 12.372.294, and 7.741.921 reads in the three control bovines and 19.533.361, 19.945.704, and 19.827.301 in three LSDV-infected bovines, respectively (Table 2). Post-cleaning, the Q20 and Q30 values exceeded 98% and 93%, respectively, for all tested samples. GC contents varied from 50.50% to 60.89% in the control and from 46.68% to 47.02% in the LSDV-infected group (Table 2). Notably, similar ncRNA length distributions were observed in the six samples of serum-derived exosomes from control and LSDV-infected bovines (data are not shown).

**Figure 1:**
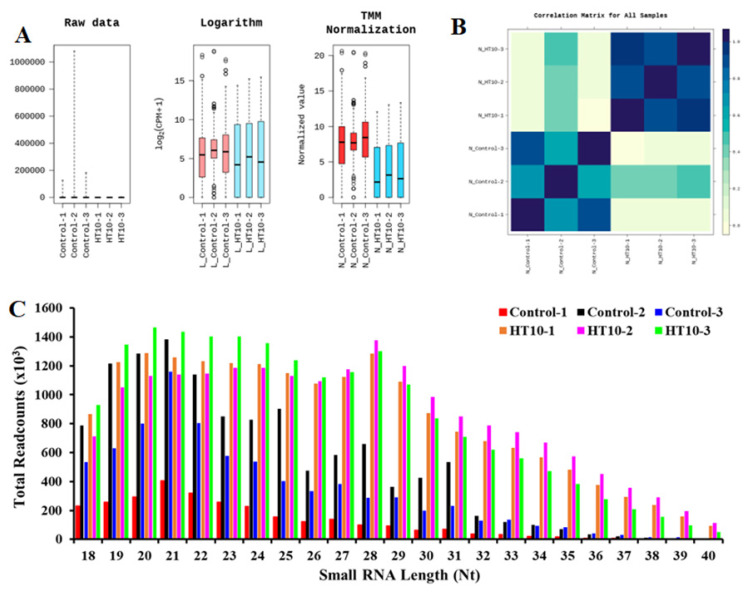
Distance analysis (A) non-coding RNAs (ncRNA) expression value (FPKM) distribution in six samples. The ordinate shows each RNA’s log10 (TPM+1) from the six sets of RNA-seq data. (B) sample correlation matrix (p<0.01, FDR<0.01). (C) Length distribution of ncRNA. Bar graphs represent the total read counts of ncRNAs in the treatment and control samples. FPKM, fragments per kilobase of transcript per million mapped reads; TPM, transcripts per million; FDR, false discovery rate.

**Figure 2:**
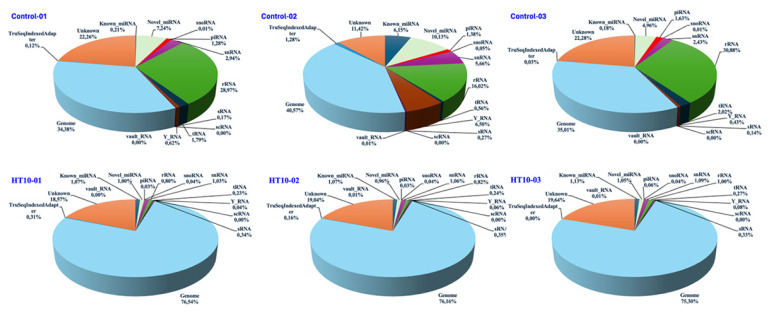
Classification of RNA types of reads in the serum samples of LSDV-infected and control cattle groups. LSDV, Lumpy skin disease virus.

All obtained sequence reads were aligned using the current bovine genome release o in the National Center for Biotechnology Information (NCBI, *Bos taurus*: ARS-UCD1.2). All groups exhibited comparable FPKM (Fragments Per Kilobase of transcript per Million mapped reads) and TPM (Transcripts Per Million) values regarding ncRNA distribution (Figure 1A). Pearson inter-sample correlation analysis of the raw read counts indicated high correlation coefficients among independent replicates for each sample, with the greatest differences and similarities observed when comparing the various samples (Figure 1B). Read lengths ranging from 19 to 34 nucleotides were the most prevalent among the annotated reads (Figure 1C). Among the annotated reads in the control group, the most unique reads were mapped to rRNAs (15.92%–30.88%), followed by snRNAs, miRNA, piRNA, tRNA, Y_RNA, sRNA, sno RNA, vault RNA, and scRNA sequences. In LSDV-infected bovines, most unique reads were mapped to miRNA (2.03%–2.13%), followed by snRNA, rRNA, tRNA, sRNA, Y-RNA, piRNA, snoRNA, vaultRNA, and scRNA sequences (Figure 2 and Table 2). The remaining unique reads that were mapped to the genome ranged from 34.38% to 40.32% in the control group and from 75.30% to 76.54% in the LSDV-infected group. Unknown sequences ranged from 11.35% to 22.28% in the control and from 18.57% to 19.64% in the LSDV-infected group (Figure 2 and Table 2).

### Differential expression analysis of non-coding RNAs

**Figure 3:**
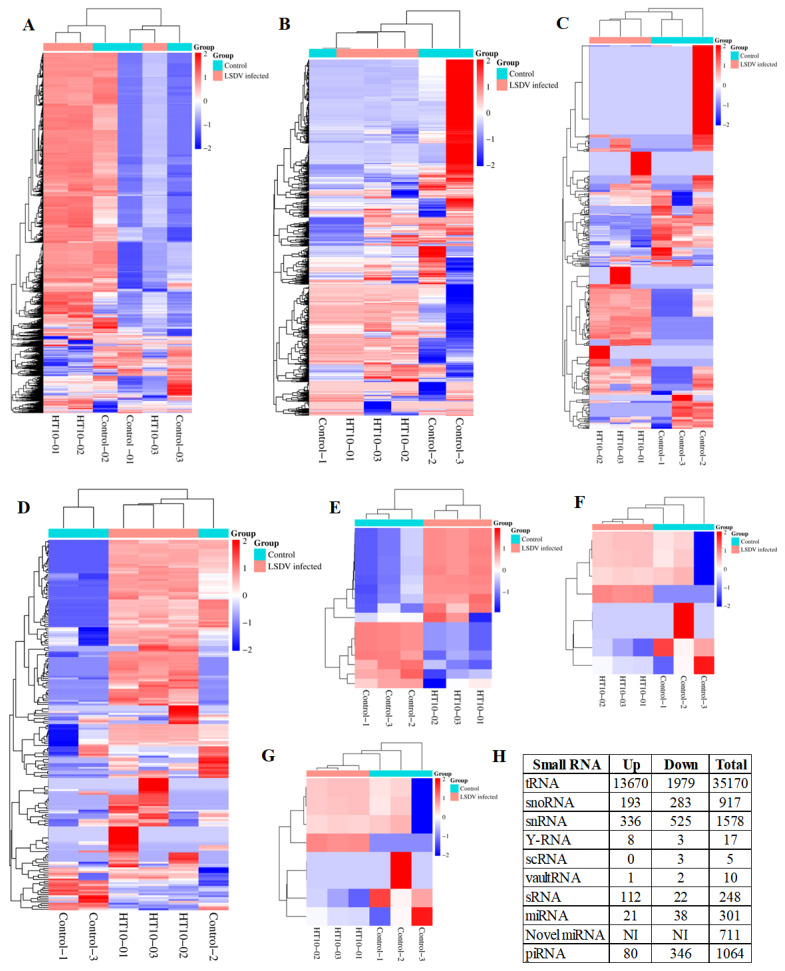
Differential expression of non-coding RNAs (ncRNAs) in the serum samples of LSDV-infected and control cattle groups (A) tRNAs, (B) snRNAs, (C) snoRNAs, (D) sRNAs, (E) Y-RNAs, (F) vault RNAs, (G) scRNAs and (H) summary of total ncRNA types. Hierarchical clustering is based on FPKMs, where log10 (FPKM+1) is used for clustering. Genes with higher expression are indicated by a red color, while genes with lower expression are indicated by a blue color. LSDV, Lumpy skin disease virus.

We compared the RPKM (reads per kilobase per million reads) values of all unique reads to identify the upregulated and downregulated ncRNAs in serum-derived exosomes from LSDV-infected bovines (Supplement 1). Based on a 2-fold cutoff threshold, 13,670 (38.86%) and 1,979 (5.62%) small tRNAs were upregulated and downregulated in LSDV-infected bovines, respectively (Figures 3A, 3H). In total, 336 (21.29%) and 525 (33.27%) snRNAs were upregulated and downregulated in LSDV-infected bovines, respectively (Figures 3B, 3H). Furthermore, 193 (21.04%) and 283 (30.86%) snoRNAs were correspondingly upregulated and downregulated (Figures 3C, 3H). Moreover, 112 (45.16%) and 22 (8.87%) sRNAs were correspondingly upregulated and downregulated (Figures 3D, 3H). Furthermore, the expression of 8 (47.05%) Y-RNAs and 1 (10%) vault RNA was increased in serum-derived exosomes from LSDV-infected bovines, while 3 (17.64%) Y-RNAs, 3 (60%) scRNAs and2 (20%) vault RNAs were downregulated (Figures 3E–3H).

### Differential expression analysis of PIWI-interacting RNAs

**Figure 4:**
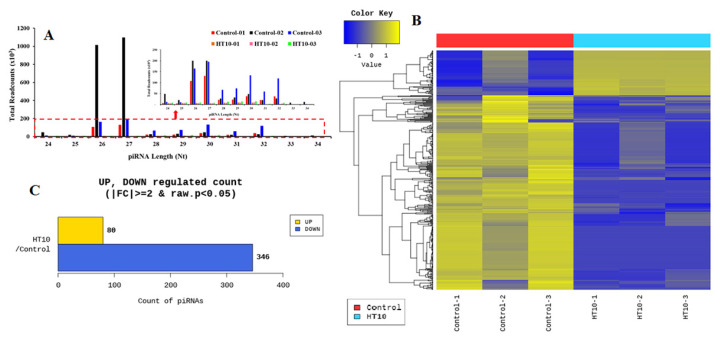
Identification and expression of PIWI-interacting RNAs (piRNAs) during LSDV infection. (A) Length distribution of piRNAs. (B) Hierarchical clustering of piRNA high-throughput sequencing data. Heat mapping and hierarchical clustering were used to analyze the PIWI-interacting RNA (piRNA) high-throughput sequencing data in the serum samples of LSDV-infected and control cattle groups based on their expression levels. The yellow line indicates high relative expression, and the blue line indicates low relative expression. (C) Upregulated and downregulated piRNAs in the serum samples of LSDV-infected bovines compared to the control non-infected individuals. LSDV, Lumpy skin disease virus.

PiRNA expression profiles were generated using the Illumina HiSeq 2000 platform to determine the piRNA transcriptome from LSDV-infected and control bovines. Clean and adapter-trimmed reads were produced, showing that piRNA lengths from both LSDV-infected and control groups ranged from 24 to 34 nucleotides (nt), with a predominant range of 26 to 32 nt (Figure 4A). A total of 1,064 piRNAs were identified from both groups based on RPKM analyses (Supplement 1). DE of piRNAs was assessed using fold change and exactTest values with edgeR. PiRNAs with a |fold change|≥2 and an exact raw p-value<0.05 were considered significantly DE. Out of the 1,064 detected piRNAs (Supplement 1), 426 exhibited significant differential expression, comprising 80 piRNAs that were significantly upregulated and 346 piRNAs that were significantly downregulated in LSDV-infected bovines compared to control groups (Figures 4B, 4C and Supplement 1).

### Target prediction and gene ontology enrichment analysis of known PIWI-interacting RNAs

**Figure 5:**
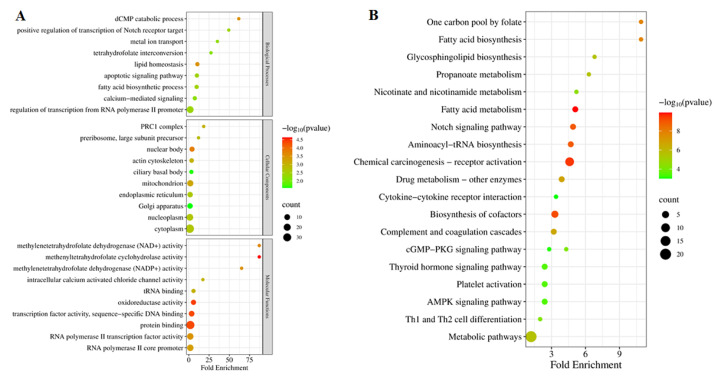
GO and KEGG analyses of 426 known DE piRNAs. (A) Top 10 GO terms of 426 known DE piRNA target genes across three GO categories (biological process, cellular component, and molecular function). Enrichment score is presented as −log10 (p-value). (B) Top 20 enriched KEGG pathways for the target genes of 426 known DE piRNAs. The sizes of the dots represent gene counts. The gene ratio indicates the ratio between the number of target genes associated with a KEGG term and the total number of genes in the KEGG terms. GO, gene ontology; DE, differentially expressed; piRNAs, PIWI-interacting RNAs.

Genes targeted by the DE piRNAs were predicted using multiple tools, including piRNAQuest V.2 (http://dibresources.jcbose.ac.in/zhumur/pirnaquest2/), airbase (http://bigdata.ibp.ac.cn/piRBase/index.php), and blast on NCBI with sequence coverage exceeding 70% and identity exceeding 80%. Subsequently, GO enrichment analysis was conducted to explore the potential functions of these target genes, aiming to infer the roles of the corresponding DE piRNAs. Of the 426 DE piRNAs, 353 were used to predict 294 unique target genes. Target genes of DE piRNAs were significantly (p<0.05, number of genes>2) enriched in 104 GO terms (51 biological processes, 21 cellular components, and 32 molecular function GO terms, respectively) (Supplement 2). The most enriched biological process GO terms were regulation of transcription from negative regulation of apoptotic signaling pathway (p = 1.1×10^−4^, 3 gene count), regulation of transcription from RNA polymerase II promoter (p = 4.2×10^−3^, 18 gene count), and apoptotic signaling pathway (p = 3.6×10^−3^, 3 gene count) (Figure 5A and Supplement 3). The most enriched cellular component GO terms included the nuclear body (p = 1.7×10^−4^, 6 gene count), mitochondrion (p = 6.5×10^−3^, 13 gene count), and cytoplasm (p = 2.2×10^−3^, 36 gene count) (Figure 5A and Supplement 2). The most enriched molecular function GO terms were protein binding (p = 4.8×10^−5^, 33 gene count), transcription factor activity, sequence-specific DNA binding (p = 4.6×10^−5^, 9 gene count), and RNA polymerase II transcription factor activity, sequence-specific DNA binding (p = 3.3×10^−4^, 15 gene count) (Figure 5A and Supplement 2)

### KEGG pathway analysis of potential targets of known PIWI-interacting RNAs

The KEGG database was utilized to analyze the 294 target genes of the DE piRNAs for their involvement in signaling pathways. This analysis was conducted using DAVID Bioinformatics Resources version 2021, with a significance threshold of p<0.05, to identify relevant pathways in cattle. Based on the results, 55 KEGG pathways were significantly enriched among the target genes of the DE piRNAs (Supplement 3), and the top 20 KEGG pathways are shown in Figure 5B. The DE piRNAs were significantly enriched in immune response signal transduction pathways (Th1 and Th2 cell differentiation, cGMP-PKG signaling pathway, cGMP-PKG signaling pathway, Notch signaling pathway, and Cytokine-cytokine receptor interactions), metabolism pathways (AMPK signaling pathway, chemical carcinogenesis-receptor activation, biosynthesis of cofactors, fatty acid metabolism, aminoacyl-tRNA biosynthesis, one carbon pool by folate, fatty acid biosynthesis, drug metabolism - other enzymes, complement, coagulation cascades, and metabolic pathways) (Figure 5B and Supplement 3). These constituted the predominant pathways and potentially are pivotal in modulating cattle response to LSDV infection.

### Validation of differentially expressed PIWI-interacting RNAs by reverse transcription quantitative real-time polymerase chain reaction

**Figure 6:**
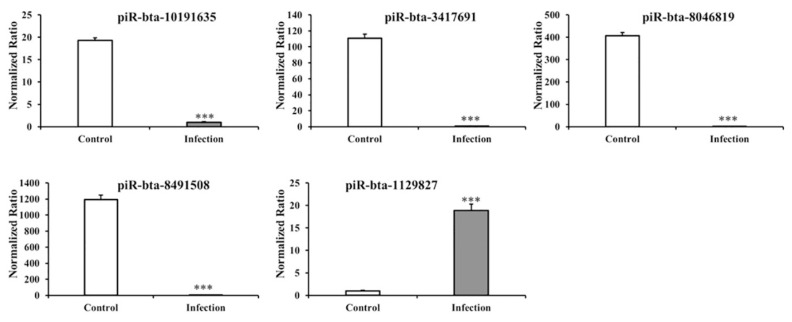
Validation of DE piRNAs by qRT-PCR. The bar graphs indicate the average fold change in individual samples. The expression of piRNAs was normalized using the expression of the bovine gene U6. The error bars indicate the SEM (*** p<0.001). Experiments were performed on individual samples in triplicate. DE, differentially expressed; piRNAs, PIWI-interacting RNAs; qRT-PCR, quantitative real-time reverse transcription polymerase chain reaction; SEM, standard error of the mean.

We validated the expression of 5 DE piRNAs (piR-bta10191635, piR-bta-3417691, piR-bta-8046819, piR-bta-8491508, and piR-bta-1129827) by RT-qPCR. The expression of these five piRNA genes was consistent with the RNA-seq results in this study, as illustrated in Figure 6. In summary, the alterations in piRNA expression in serum-derived exosomes from LSDV-infected bovines may indicate that these animals exhibit intricate molecular responses mediated by piRNAs during viral infection.

## DISCUSSION

Understanding the intricate roles of ncRNAs in infectious cattle diseases is essential for elucidating host-pathogen interactions, pinpointing potential therapeutic targets, and developing disease control and management strategies. However, there is a dearth of information regarding the ncRNA profile, particularly the expression of piRNAs in serum-derived exosomes from LSDV-infected bovines. In this study, we performed RNA-sequencing to identify different ncRNA types, particularly focusing on piRNA expression in serum-derived exosomes from LSDV-infected compared with control groups.

Based on our findings, a total of 35.170 tRNAs, 917 snoRNAs, 1.578 snRNAs, 17 Y-RNAs, 5 scRNAs, 10 vault RNAs, 248 sRNAs, 1064 piRNAs, and 1011 miRNAs (data not shown) were expressed in serum-derived exosomes from LSDV-infected bovines. Additionally, 15,649 DE tRNAs, 476 snoRNAs, 861 snRNAs, 11 Y-RNAs, 3 scRNAs, 3 vault RNAs, 134 sRNAs, and 426 piRNAs were identified in serum-derived exosomes from LSDV-infected when compared to control non-infected individuals. ncRNAs are crucial in regulating gene expression and have pivotal functions in various biological processes, including the pathogenesis of infectious diseases [13,14]. For example, previous research indicated that alternative splicing, regulated by snRNA and spliceosome, could generate distinct protein isoforms involved in the immune response. Isoforms may differ with regard to immune signaling functions, pathogen recognition, or immune cell activation. snRNAs are essential for spliceosome function and post-transcriptional gene regulation. Although seldom directly associated with infections, their role in cellular health and gene expression, including host-pathogen interactions, is crucial [24]. tRNAs are traditionally recognized for their roles in protein synthesis, acting as adaptors that decode mRNA into amino acids to form proteins [24]. However, emerging research has revealed that tRNAs and their associated fragments, termed tRNA-derived fragments (tRFs), significantly contribute to various biological processes, including the pathogenesis and response to infectious diseases, through their association and binding with gametocyte-specific factor 1 (Asterix/Gtsf1) or p53 activation [25]. Our findings may indicate that tRNAs may be important in regulating viral replication or immune responses in LSDV-infected cattle. Further validation of identified ncRNAs, such as tRNAs or snRNAs is needed to confirm their biological functions and possible use as biomarkers for LSDV infection in cattle.

piRNAs have emerged as crucial regulators in the defense system against viral infections [26]. Traditionally known for their function in the germline to safeguard against transposon activity and maintain genomic integrity, research suggests that piRNAs also significantly contribute to immune defense against viral infections in somatic cells [27,28]. Moreover, piRNAs have been identified as regulators of immune response gene expression and modulators of the inflammatory response during viral infections, highlighting their importance in shaping the immune response to infectious diseases [29]. Despite their established roles, piRNA expression patterns during LSDV infection in bovines remain largely unknown, particularly in serum-derived exosomes. We observed that 80 piRNAs were upregulated and 346 piRNAs were downregulated in serum-derived exosomes from LSDV-infected bovines compared to non-infected controls. GO analyses of the target genes of these piRNAs revealed significant associations with metabolism, cell signaling, and immune response pathways. KEGG analysis further indicated that these piRNAs regulate immune responses including cytokine-cytokine receptor interactions, the Notch signaling pathway, the cGMP-PKG signaling pathway, the Th1-Th2 cell differentiation pathway or metabolism-related pathways such as the AMPK signaling pathway, fatty acid metabolism, glycosphingolipid biosynthesis and propanoate metabolism. GO and pathway analyses also indicated that piRNAs regulate metabolism, cell signaling, and immune response pathways via their target genes. Small RNA sequencing of LSDV-infected and control bovines identified numerous DE piRNAs. Using RT-qPCR, we confirmed the upregulation or downregulation of six piRNAs in the serum-derived exosomes from LSDV-infected individuals compared to controls. These findings indicate that piRNAs may contribute to the advancement of LSDV, although additional evidence is required.

Previous studies have highlighted the critical roles of the Notch signaling pathway, the cGMP-PKG signaling pathway, the cytokine-cytokine receptor interactions, and the Th1-Th2 cell differentiation pathway in viral entry and progression through various biological processes [30,31]. Notch signaling is pivotal in the immune system for the development and functions of multiple cell types, including B cells, T cells, dendritic cells, and macrophages [30,32,33]. Active Notch signaling has been observed in various inflammatory conditions such as rheumatoid arthritis, cancer, PRRS, IBD, the Influenza virus H1N1, Echinococcus, the respiratory syncytial virus, Japanese encephalitis virus, *Mycobacterium bovis* BCG, tuberculin, *Ehrlichia chaffeensis*, and LPS, through its association with and activation of Toll-like receptors, NF-κB, and MAPK pathways [30,32–34]. The cGMP pathway regulates the production of nitric oxide, gene transcription, cell programming, and natriuretic peptides, influencing various physiological processes [31,35]. This pathway also plays a role in the replication of several viruses, such as PRRS, PCV, avian influenza, ASFV, and IBD virus [22,36]. The Th1-Th2 cell differentiation pathway is crucial for mounting host defenses against intracellular pathogens, including protozoa, bacteria, and viruses, and is also involved in certain autoimmune diseases [37,38]. Cytokine and cytokine receptor interaction networks are critical in orchestrating inflammatory and anti-inflammatory responses and serve as genetic markers for significant infectious or zoonotic diseases such as the Influenza virus, IBD, FMDV, ASFV, PRRS, PCV2, CSFV, Tuberculosis, Salmonella, and cancer [20,22,39]. Moreover, cytokine and cytokine receptor interactions can activate and regulate cell differentiation. For instance, Th1 cell differentiation is induced by IL-12 or IFN-γ, which activate STAT4 or STAT1, respectively. Th2 cell differentiation is initiated by IL-4, activating STAT6, while Th17 cell differentiation is induced by TGF-β in conjunto with IL-6 (or IL-21), activating STAT3 [40–42]. Our findings suggest that the cytokine-cytokine receptor interactions, as well as the Notch, cGMP-PKG, and Th1-Th2 cell differentiation pathways, may be crucial in modulating inflammatory cytokine expression rather than viral replication in cattle during LSDV infection. Specifically, piRNAs present in serum-derived exosomes may affect the regulation of LSDV replication via these immune or signaling pathways.

## CONCLUSION

In this study, we have identified multiple ncRNAs that may play significant roles in the replication and defense-modulation against LSDV in infected bovines. Despite their identification, the functions of these ncRNAs remain largely unknown. Our analyses provide a comprehensive list of piRNAs, snRNAs, tRNAs, Y_RNAs, sRNAs, snoRNAs, vault RNAs, and scRNAs, with a particular focus on piRNAs in serum-derived exosomes of LSDV-infected bovines compared to their expression in non-infected individuals. Additional examination and confirmation of the functions of these ncRNAs will enable us to link these distinct small RNAs with their roles in modulating LSDV infection in cattle. This could lead to the development of prospective biomarkers for the control of LSDV in cattle. Furthermore, a more profound comprehension of their functions in LSDV signaling pathways may reveal innovative therapeutic targets.

## Figures and Tables

**Table 1:** Primers targeting bovine piRNAs used for real-time quantitative polymerase chain reaction (RT-qPCR) analyses in this study

piRNAs	Sequences (5′→3′)
piR-bta10191635	AGCTCAGTCGGTAGAGCATCAGAC
piR-bta-3417691	TCGCCGTGATCGTATAGTGGTTAGTACTCTG
piR-bta-8046819	CCTGGGCAACATAGCGAGACCCCGTCTCTA
piR-bta-8491508	CACCGCCGCGGCCCGGGTTCGATTCCCGGT
piR-bta-1129827	GTGCTCAGTAAATATTTGTGAAATGAATGA
U6-F	CTCGCTTCGGCAGCACATATACT
U6-R	ACGCTTCACGAATTTGCGTGTC

piRNAs, PIWI-interacting RNAs.

**Table 2:** Classification statistics of small RNA sequences in control and LSDV-infected bovines

Items	Control-1	Control-2	Control-3	HT10-1	HT10-2	HT10-3
Small RNA data statistics						
Raw total bases	915.554.958	2.150.018.730	2.385.501.183	2.195.912.253	2.075.959.539	2.130.712.782
Raw read count	17.952.058	42.157.230	46.774.533	43.057.103	40.705.089	41.778.682
Raw GC (%)	22.15	37.76	20.96	39.81	39.37	39.84
Raw Q20 (%)	90.12	92.17	90.43	92.94	93.38	92.7
Raw Q30 (%)	83.37	84.78	83.87	85.7	86.46	85.14
Clean total bases	67.195.415	302.920.799	184.376.126	518.187.235	541.494.662	511.076.577
Clean read count	2.876.226	12.372.294	7.741.921	19.533.361	19.945.704	19.827.301
Clean GC (%)	50.5	60.89	50.66	46.68	47.16	47.02
Clean Q20 (%)	98.07	98.09	98.02	98.24	98.33	98.15
Clean Q30 (%)	93.93	94.15	93.82	94.44	94.78	94.4
Annotation read count	2.117.434	10.798.041	5.522.022	19.501.812	19.911.858	19.789.907
Mapped_reads	1.015.584 (47.96%)	7.489.394 (69.36%)	2.547.755 (46.14%)	13.877.543 (71.16%)	14.106.381 (70.84%)	13.955.506 (70.52%)
Unmapped_reads	1.101.850 (52.04%)	3.308.647 (30.64%)	2.974.267 (53.86%)	5.624.269 (28.84%)	5.805.477 (29.16%)	5.834.401 (29.48%)
Total small RNA read count						
Known_miRNA	6.119 (0.21%)	755.397 (6.11%)	14.082 (0.18%)	208.208 (1.07%)	212.598 (1.07%)	224.856 (1.13%)
Novel_miRNA	208.256 (7.24%)	1.325.182 (10.71%)	384.038 (4.96%)	195.162 (1.0%)	192.142 (0.96%)	207.987 (1.05%)
piRNA	36.698 (1.28%)	169.436 (1.37%)	125.922 (1.63%)	5.542 (0.03%)	6.635 (0.03%)	11.098 (0.06%)
snoRNA	231 (0.01%)	6.368 (0.05%)	1.006 (0.01%)	6.958 (0.04%)	7.089 (0.04%)	6.981 (0.04%)
snRNA	84.490 (2.94%)	694.948 (5.62%)	187.901 (2.43%)	201.436 (1.03%)	211.830 (1.06%)	215.301 (1.09%)
rRNA	833.372 (28.97%)	1.969.562 (15.92%)	2.390.850 (30.88%)	156.275 (0.8%)	163.117 (0.82%)	199.165 (1.0%)
tRNA	51.405 (1.79%)	69.313 (0.56%)	156.345 (2.02%)	4.5089 (0.23%)	47.503 (0.24%)	53.941 (0.27%)
Y_RNA	17.957 (0.62%)	798.632 (6.46%)	33.365 (0.43%)	7.977 (0.04%)	11.207 (0.06%)	16.308 (0.08%)
scRNA	5 (0.0%)	0 (0.0%)	0 (0.0%)	0 (0.0%)	0 (0.0%)	0 (0.0%)
vault_RNA	13 (0.0%)	1.041 (0.01%)	26 (0.0%)	709 (0.0%)	1.003 (0.01%)	1257 (0.01%)
sRNA	4.884 (0.17%)	33.669 (0.27%)	10.714 (0.14%)	65.694 (0.34%)	69.355 (0.35%)	64.752 (0.33%)
Other						
Genome	988.960 (34.38%)	4.988.053 (40.32%)	2.710.697 (35.01%)	14.951.657 (76.54%)	15.193.120 (76.17%)	14.930.833 (75.3%)
TruSeq Indexed Adapter	3.534 (0.12%)	15.6691 (1.27%)	2.401 (0.03%)	60.653 (0.31%)	31.833 (0.16%)	495 (0.0%)
Unknown	640.302 (22.26%)	1.404.002 (11.35%)	1.724.574 (22.28%)	3.628.001 (18.57%)	3.798.272 (19.04%)	3.894.327 (19.64%)

## SUPPLEMENTARY MATERIAL

**Supplement 1**. piRNA-expression.

**Supplement 2**. Gene ontology enrichment analysis.

**Supplement 3**. KEGG pathway enrichment analysis.
