# Supplementary material for: Exosomal small non-coding RNA profiling and the role of PIWI-interacting RNA pathway genes in Lumpy skin disease virus-infected bovines
Source: Anim Biosci. 2025 Jun 4;38(11):2364–76. doi: 10.5713/ab.25.0217 (PMC12580968; doi:10.5713/ab.25.0217)
Supplement: Supplementary file 1 [file ab-25-0217-supplementary-1.pdf]

Supplement 1. piRNA-expression

	·	Sequence	Location	HT10/Control.fc	HT10/Control.logC	HT10/Control.raw.pv	HT10/Control.bh.pval	Control.mean	HT10.mean
1	piR-bta-18464269	GAGCCCGGATAGCTCAGTCGGTAGAGC	23:30029713-30029740:-	-93.043966	6.280956	0.000165583	0.000357346	6.576765	0.770737
2	piR-bta-4161779	GTTTCCGTAGTGTAGTGGTCATCACGTTCGCC	3:21035264-21035296:+	-93.840796	13.963849	0.000192506	0.000408267	14.163871	7.289687
3	piR-bta-512593	GGTGGTTCAGTGGTAGAATTCTCGC	3:20877480-20877505:+	-10150.937384	10.987878	9.03516E-09	4.52587E-08	10.835591	0.000000
4	piR-bta-399832	GGGTCGGGGTTTCGTACGTAGCAGAGCAGC	2:121574990-121575020:-	4.094195	10.705095	0.038835009	0.051843313	9.173185	11.384528
5	piR-bta-7143852	ACACGCAGAAGGTCCTGGGTTCGAGCCCCAGTGG	15:82766877-82766911:-	-79.789599	7.278546	2.94534E-08	1.32905E-07	8.065680	1.673750
6	piR-bta-1210928	CTGGGTCGGGGTTTCGTACGTAGCAG	2:121574996-121575022:-	4.045720	10.750689	0.043119213	0.057005062	9.191187	11.427156
7	piR-bta-5010085	AGCTCAGTCGGTAGAGCATCAGACTT	23:31093151-31093177:-	-92.977341	7.034878	2.53697E-07	9.17341E-07	7.896366	1.053041
8	piR-bta-4102797	GACGAGGTGGCCGAGTGGTTAAGGCAATGGA	19:27830142-27830173:+	-5.051809	6.570954	0.022315227	0.03202143	7.005660	4.715786
9	piR-bta-1455465	GCCCGGCTAGCTCAGTCGGTAGAGCATGAGAC	3:45753112-45753144:+	-39.874742	7.758051	5.35975E-05	0.000130063	8.498884	1.677535
10	piR-bta-16262955	GCCTGGATAGCTCAGTTGGTAGAGCATCAGA	24:12411762-12411793:-	-80.978708	7.297109	8.44495E-09	4.34995E-08	8.153949	1.673750
11	piR-bta-10967861	GCTGCCTTCCAAGCAGTTGACCCGGG	19:27846594-27846620:+	-114.105033	4.803095	0.001286948	0.002342245	5.390347	0.000000
12	piR-bta-558649	ATAGCTCAGTCGGTAGAGCATCAGAC	15:82774088-82774114:-	-109.483340	7.260519	7.0723E-08	2.90336E-07	8.072631	1.053041
13	piR-bta-1953525	TGAGGTAGTAGGTTGTATAGTTAG	5:116408412-116408436:+	-34.207297	11.839945	8.61829E-06	2.29541E-05	12.716313	7.699024
14	piR-bta-4542634	GGATAGCTCAGTCGGTAGAGCATCAGAC	16:2247973-2248001:+	-125.128157	7.441531	5.19256E-09	3.22175E-08	8.337513	1.053041
15	piR-bta-1329249	CTTCTGGGTCGGGGTTTCGTACGTAG	16:25007219-25007245:+	9.222685	9.832332	0.011228662	0.017157042	5.560225	10.675148
16	piR-bta-2922035	CCGTAGTGTAGTGGTTATCACGTTCGCC	3:21128488-21128516:-	-137.477039	8.944560	4.65868E-07	1.56051E-06	9.720021	1.427184
17	piR-bta-1526861	TTCTGGGTCGGGGTTTCGTACGTAGCAG	rRNA rRNA LSU-rRNA_Hsa;	3.938055	10.758393	0.045821037	0.059790847	9.212804	11.427156
18	piR-bta-10755347	AGGTCCCGGGTTCAAATCCCGGACGAGCCCCCA	15:55651371-55651404:-	-64.969212	8.607980	2.77875E-08	1.26433E-07	9.507080	3.513633
19	piR-bta-952149	TTCCGTAGTGTAGTGGTTATCACGTTCGCCTC	3:20957585-20957617:+	-185.405634	9.700753	1.28174E-07	5.1458E-07	10.459919	1.542514
20	piR-bta-1883372	GTTTCCGTAGTGTAGTGGTTATCACTTTCGCC	3:21035264-21035296:+	-98.944428	14.084985	0.0001376	0.000306652	14.292042	7.445521
21	piR-bta-853808	TTCTGGGTCGGGGTTTCGTACGTA	16:58623635-58623659:-	10.091765	9.621071	0.006440753	0.010373602	5.438319	10.474768
22	piR-bta-6446	GCGGCCCGGGTTCGATTCCCGGTCAGG	NKLS02000508.1:29784-29811	-68.163695	10.915207	4.03286E-07	1.37622E-06	11.736100	5.734121
23	piR-bta-18470437	AGCCCGGATAGCTCAGTCGGTAGAG	23:30029714-30029739:-	-293.290642	5.982678	9.29853E-05	0.000210664	6.325836	0.000000
24	piR-bta-10281522	TGGGTGGTTCAGTGGTAGAATTCTCGC	13:21370304-21370331:-	-11064.596503	11.112091	6.22654E-09	3.54134E-08	10.972375	0.000000
25	piR-bta-153457	TCGGGGTTTCGTACGTAGCAGAGCAGCTC	1:85343020-85343049:+	5.526440	7.382071	0.002047679	0.003583439	5.131605	8.087825
26	piR-bta-578587	GTTCAGTGGTAGAATTCTCGCCTG	2:39839401-39839425:+	-3624.998154	9.505686	1.23373E-09	2.13306E-08	9.865391	0.000000
27	piR-bta-462815	CTGGGTCGGGGTTTCGTACGTAGCAGA	16:58623630-58623657:-	4.042913	10.750877	0.042557862	0.056399496	9.191726	11.427156
28	piR-bta-4037872	GTTTCCGTAGTGTAGTGGTCATCACGTTCGC	7:40358384-40358415:-	-90.840393	13.810034	0.000262653	0.00054736	13.780796	7.223542
29	piR-bta-1530943	TGGTTCAGTGGTAGAATTCTCGCCTGC	NKLS02000508.1:23492-23519	-7735.494054	10.596547	1.39476E-09	2.17583E-08	10.868963	0.000000
30	piR-bta-747118	GTTCAGTGGTAGAATTCTCGCCTGCC	17:61128911-61128937:+	-3735.973344	9.549356	3.09726E-10	2.11388E-08	10.031392	0.000000
31	piR-bta-1308635	GCCCGGATAGCTCAGTCGGTAGAGCATCA	23:30029709-30029738:-	-36.406788	6.899786	2.32644E-05	5.96355E-05	7.522672	2.070916
32	piR-bta-13367656	AGGCCCCGGGTTCGATCCCCGGCATCTCCAC	17:50880782-50880813:+	-44.405748	5.898404	0.002777999	0.00465623	4.847719	0.882888
33	piR-bta-522967	GCCCGGCTAGCTCAGTCGGTAGAGCATGAGACT	25:2629352-2629385:+	-40.639255	7.782556	3.7024E-05	9.15282E-05	8.537234	1.677535
34	piR-bta-1407187	GCCCGGCTAGCTCAGTCGGTAGAGCAT	25:2627613-2627640:+	-57.171527	5.638160	0.00034538	0.000703647	6.294566	0.770737
35	piR-bta-16910604	TCAGTCGGTAGAGCATCAGACTTTTAA	19:27761060-27761087:+	-55.784235	6.347626	4.62498E-05	0.000112734	7.196158	1.053041
36	piR-bta-2798878	GGTAGCGTGGCCGAGCGGTCTAAGGCGCTG	23:30293278-30293308:+	-50.005048	5.482923	0.001588065	0.002880677	5.721722	0.770737
37	piR-bta-382338	TCTGGGTCGGGGTTTCGTACGTAGCAGA	16:58623630-58623658:-	3.976568	10.755579	0.043856974	0.057562278	9.205008	11.427156
38	piR-bta-3777300	CACGCGGGAGGCCCGGGTTCGATTCCCGGC	18:1819459-1819489:+	-4.821004	8.640141	0.007255922	0.011652157	9.271385	7.081279
39	piR-bta-5197584	CGTGAGTTCGATCCTCACACGGGGCACCA	14:16621779-16621808:+	-17.806933	7.029454	0.002780093	0.00465623	5.637386	3.729211
40	piR-bta-12008687	GATAGCTCAGTCGGTAGAGCATCAGA	16:2247974-2248000:+	-99.561873	7.128954	1.38753E-07	5.41136E-07	7.940464	1.053041
41	piR-bta-64860	GTGGTTCAGTGGTAGAATTCTCGCCT	23:31686625-31686651:+	-9588.836565	10.905863	4.23756E-09	3.01423E-08	10.933024	0.000000
42	piR-bta-494197	TGGGTCGGGGTTTCGTACGTAGCAGAGCA	16:58623627-58623656:-	3.844597	10.754402	0.049285962	0.063499393	9.251676	11.415939
43	piR-bta-1665756	CATTGGTGGTTCAGTGGTAGAATTCTCGCC	23:30943694-30943724:+	-19227.377705	11.908768	5.32005E-09	3.26376E-08	11.669121	0.000000
44	piR-bta-255405	TGCTTCTGGGTCGGGGTTTCGTACGT	16:58623636-58623662:-	8.527315	9.574728	0.012211514	0.018418472	5.502376	10.404416
45	piR-bta-704967	TTCTGGGTCGGGGTTTCGTACGTAG	16:58623634-58623659:-	9.756453	9.823417	0.008684837	0.013548346	5.530051	10.673983
46	piR-bta-11055413	TCTCGCCTGCCACGCGGGAGGCCCGG	3:8034319-8034345:+	-96.134977	4.603265	0.002389839	0.004059896	5.220745	0.000000
47	piR-bta-1704508	TAGCTTATCAGACTGATGTTGACA	19:10803478-10803502:+	-220.204804	8.100333	1.72833E-08	8.27781E-08	8.778616	0.882888
48	piR-bta-10521165	GGGGGTATAGCTCAGTGGCAGAGCATTTGA	17:56539852-56539882:-	-2161.635665	14.695958	2.98832E-07	1.0595E-06	13.873815	4.388659
49	piR-bta-4552673	CTCGCCTGCCACGCGGGAGGCCCGGG	2:39839417-39839443:+	-102.568495	4.680219	0.001734351	0.003084547	5.300436	0.000000
50	piR-bta-352070	GCTAGCTCAGTCGGTAGAGCATGAGACT	25:2633976-2634004:+	-673.005154	7.113812	3.37921E-09	2.80152E-08	7.928191	0.000000
51	piR-bta-3151217	ACGCGGGAGGCCCGGGTTCGATTCCCGG	18:1819460-1819488:+	-4.135326	8.437472	0.017443853	0.025534434	8.946200	7.056152
52	piR-bta-8046819	CCTGGGCAACATAGCGAGACCCCGTCTCTA	15:40852004-40852034:-	-46.385440	8.787181	0.002985534	0.004954715	6.537216	3.124389
53			3:78116895-78116925:-						
54	piR-bta-802061	GTGGTTCAGTGGTAGAATTCTCGCCTGCC	NKLS02000508.1:23491-23520	-9850.867857	10.944846	9.91544E-10	2.13306E-08	11.215713	0.000000
55	piR-bta-4504416	CCGGATAGCTCAGTCGGTAGAGCATCAGA	23:30029707-30029736:-	-176.770504	7.932593	3.28679E-09	2.80152E-08	8.879487	1.053041
56	piR-bta-1675519	TCAGTGGTAGAATTCTCGCCTGCC	NKLS02000508.1:4495-4519:+	-1669.123379	8.393807	4.53313E-09	3.0184E-08	8.968589	0.000000
57	piR-bta-1896697	TGAGGTAGTAGGTTGTATAGTTCT	8:85450401-85450425:+	-35.557611	11.832390	6.25343E-06	1.71577E-05	12.714497	7.629306
58	piR-bta-1401945	TCGGGGTTTCGTACGTAGCAGAGC	1:85343020-85343044:+	8.254856	7.319415	8.18063E-05	0.000190069	4.796257	8.087825
59	piR-bta-1053888	TCCAGGGTTCAAGTCCCTGTTCGGGCG	23:30029665-30029692:-	-48.983303	5.348633	0.000359201	0.000721043	5.809081	0.620709
60	piR-bta-405069	TTGGTGGTTCAGTGGTAGAATTCTCGCCTGCC	18:1416099-1416131:-	-12186.699212	11.251483	6.74833E-10	2.13306E-08	11.499702	0.000000
61	piR-bta-1416247	GGTTCAGTGGTAGAATTCTCGCCTGC	3:8034303-8034329:+	-6707.642455	10.391296	1.19354E-09	2.13306E-08	10.693913	0.000000
62	piR-bta-990162	TGGTTCAGTGGTAGAATTCTCGCCT	NKLS02000508.1:31733-31758	-7515.110891	10.554740	6.16928E-09	3.54134E-08	10.607265	0.000000
63	piR-bta-4595426	CCGCGGCCCGGGTTCGATTCCCGGTCAGG	NKLS02000508.1:1278-1307:-	-72.866834	11.168467	6.33092E-07	2.08234E-06	11.940691	5.865409
64	piR-bta-10253910	CCTGGTGGTCTAGTGGTTAGGATTTGGC	NKLS02002208.1:8011011-801	-170.303022	11.329242	6.85692E-08	2.83627E-07	11.932756	4.841230
65	piR-bta-6575840	AACATTCAACGCTGTCGGTGAGTG	11:95709010-95709034:+	-49.502072	5.497720	0.009622406	0.014883381	4.270010	0.721544
66			16:77762898-77762922:-						
67	piR-bta-5875198	CGGCTAGCTCAGTCGGTAGAGCATGAG	25:2629355-2629382:+	-709.416605	7.188091	1.22242E-09	2.13306E-08	8.043199	0.000000
68	piR-bta-6447234	CTCGCCTGCCACGCGGGAGGCCCGG	2:39839417-39839442:+	-96.138586	4.603265	0.002345852	0.004003078	5.220745	0.000000
69	piR-bta-1606549	GATAGCTCAGTCGGTAGAGCATCAGAC	15:82772310-82772337:+	-110.624508	7.273785	4.56666E-08	1.918E-07	8.097567	1.053041
70	piR-bta-2864346	GCTTCTGGGTCGGGGTTTCGTACGTAG	16:58623634-58623661:-	8.930474	9.836958	0.012842012	0.019210243	5.577204	10.675148
71	piR-bta-3250391	TAGCTCAGTCGGTAGAGCATCAGA	23:30029707-30029731:-	-96.770306	7.091731	3.36106E-07	1.16888E-06	7.862934	1.053041
72	piR-bta-3250431	AGCTCAGTCGGTAGAGCATGAGACTCTT	25:2638938-2638966:+	-181.056449	7.083514	5.58213E-09	3.33017E-08	7.881107	0.620709
73	piR-bta-1420574	ATGGGTGGTTCAGTGGTAGAATTCTCGC	13:21370304-21370332:-	-15869.491286	11.631984	4.8614E-09	3.08642E-08	11.330165	0.000000
74			4:85606090-85606118:-						
75	piR-bta-978534	GGTCGGGGTTTCGTACGTAGCAGAG	1:85343018-85343043:+	6.180254	8.981950	0.004791183	0.007832293	6.908319	9.740973
76	piR-bta-248617	AGCTCAGTCGGTAGAGCATGAGACT	25:2622404-2622429:+	-586.473618	6.921240	1.43934E-08	6.95471E-08	7.771879	0.000000
77	piR-bta-7286419	CCTGCTTCTGGGTCGGGGTTTCGTACGTAG	16:58623634-58623664:-	7.974993	9.856355	0.016414589	0.02422261	5.941750	10.677329
78	piR-bta-4754231	TCCGTAGTGTAGTGGTTATCACGTTCGCCTCA	3:21086762-21086794:-	-167.001911	9.551066	2.09153E-07	7.82173E-07	10.342399	1.542514
79	piR-bta-402045	GTGGTTCAGTGGTAGAATTCTCGCC	2:69790198-69790223:+	-9543.531451	10.899018	5.14892E-09	3.22175E-08	10.874231	0.000000
80	piR-bta-2399945	TAGCTTATCAGACTGATGTTGAAT	19:10803478-10803502:+	-203.906199	7.994562	7.8591E-09	4.24859E-08	8.721156	0.882888
81	piR-bta-398522	CTTCTGGGTCGGGGTTTCGTACGT	16:58623636-58623660:-	9.276655	9.561050	0.008714084	0.013555242	5.456802	10.403021
82	piR-bta-1298592	GCTCAGTCGGTAGAGCATCAGACTTTT	23:30158667-30158694:+	-83.652459	6.891167	8.13456E-07	2.62809E-06	7.759591	1.053041
83	piR-bta-19415484	CCCTGGTAGTCTAGTGGTTAGGATTCGG	29:17466361-17466389:+	-242.667340	11.436109	8.1187E-07	2.62809E-06	11.544292	4.091510
84	piR-bta-1702297	GTTCGACTCCCGGTATGGGAACCA	2:1313366-1313390:-	-310.295093	6.037958	2.53881E-05	6.41755E-05	6.698279	0.000000
85	piR-bta-1696485	GCCCGGATAGCTCAGTCGGTAGAG	23:30029714-30029738:-	-293.303273	5.982678	8.78188E-05	0.000200624	6.325836	0.000000
86	piR-bta-1061676	GCTTCTGGGTCGGGGTTTCGTACGTAGC	16:58623633-58623661:-	4.549567	10.682919	0.038727348	0.051826304	8.743498	11.391378
87	piR-bta-1378682	GTGGTTCAGTGGTAGAATTCTCGCCTG	5:23826387-23826414:+	-9734.018144	10.927612	1.62707E-09	2.33785E-08	11.115771	0.000000
88	piR-bta-1534598	GCCCGGCTAGCTCAGTCGGTAGAGCATG	3:45753112-45753140:+	-81.400719	6.096882	9.6528E-05	0.000217786	6.656110	0.770737
89	piR-bta-18372126	GAGCCCGGATAGCTCAGTCGGTAG	23:30029716-30029740:-	-258.994174	5.808726	0.000274592	0.000567906	5.990348	0.000000
90	piR-bta-1150257	GATAGCTCAGTCGGTAGAGCATCAGACTTT	16:2248341-2248371:-	-122.750605	7.418509	2.11667E-08	9.79408E-08	8.222567	1.053041
91	piR-bta-9241657	GGCTCGTTGGTCTAGGGGTATGATTCTCGG	15:55651416-55651446:-	-86.578040	8.500377	6.0641E-07	2.00667E-06	9.224442	2.148728
92	piR-bta-5937147	TCAGTCGGTAGAGCATCAGACTTT	15:82774085-82774109:-	-55.202630	6.334449	6.10082E-05	0.000146742	7.177950	1.053041
93	piR-bta-8465728	CTAGACTGAAGCTCCTTGAGGTTT		-188.086754	8.022191	4.36124E-09	3.01423E-08	8.948144	1.053041
94	piR-bta-680657	TTCCAAGCAGTTGACCCGGGTTCG	19:27846600-27846624:+	-47.399537	3.801057	0.049804332	0.063845613	3.683433	0.000000
95	piR-bta-278371	GGGTCGGGGTTTCGTACGTAGCAGAGC	16:58623628-58623655:-	4.196048	10.698271	0.034975795	0.04774196	9.152536	11.384528
96	piR-bta-11028797	CGACCTGCTTCTGGGTCGGGGTTTCGTAC	16:58623638-58623667:-	-21.561586	5.254762	0.030559534	0.042241786	3.695733	1.492281
97	piR-bta-1404645	TGGTTCAGTGGTAGAATTCTCGCC	3:20877482-20877506:+	-7469.823473	10.545998	8.11506E-09	4.26887E-08	10.534776	0.000000
98	piR-bta-9008335	GGGGGGTGTAGCTCAGTGGTAGAGCGCGTGC	23:30156914-30156945:+	-27.632243	12.207496	0.000293501	0.000602449	12.439434	8.280299
99	piR-bta-3055746	TGGTTCAGTGGTAGAATTCTCGCCTCC	28:40004083-40004110:+	-7725.373488	10.594649	1.5022E-09	2.27833E-08	10.851240	0.000000
100	piR-bta-253523	TCGCCTGCCACGCGGGAGGCCCGGGT	3:8034321-8034347:+	-20.259103	4.962957	0.017345667	0.025458963	4.037790	0.882888
101	piR-bta-111972	GCCCGGATAGCTCAGTCGGTAGAGCAT	23:30029711-30029738:-	-102.371920	6.408481	6.57341E-05	0.000157416	6.728703	0.770737
102	piR-bta-2418075	CGCGGGTTCGATCCCCGTACGGGCCACC	23:31723699-31723727:+	-9.667786	7.481766	0.000697341	0.001340663	7.836645	4.963225
103	piR-bta-6031023	AGCCCGGCTAGCTCAGTCGGTAGAGCATGAG	3:45753111-45753142:+	-76.227995	7.675955	9.98273E-07	3.11461E-06	8.388666	1.358674
104	piR-bta-240810	TGCTTCTGGGTCGGGGTTTCGTACGTAG	16:58623634-58623662:-	8.602602	9.843669	0.015085294	0.022442972	5.597310	10.676310

	N_Control-1	N_Control-2	N_Control-3	N_HT10-1	N_HT10-2	N_HT10-3	Control-1_Re	Control-2_Re	Control-3_Re	HT10-1_Read	HT10-2_Read	HT10-3_Read	Control-1_RP	Control-2_RP	Control-3_RP	HT10-1_RPM	HT10-2_RPM	HT10-3_RPM
1	4.749040	6.765960	8.215296	0.000000	2.312210	0.000000	2	80	41	0	1	0	5.115783	33.926956	48.958030	0.000000	18.045981	0.000000
2	15.191259	11.568821	15.731534	5.912168	6.177834	9.779060	2890	2253	7531	17	18	333	7392.306374	955.467901	8992.754212	295.677885	324.827661	3734.858681
3	12.268307	7.450215	12.788250	0.000000	0.000000	0.000000	381	129	979	0	0	0	974.556653	54.707217	1169.022225	0.000000	0.000000	0.000000
4	8.697845	10.132440	8.689271	11.415785	11.359079	11.378720	32	832	57	784	662	1010	81.852527	352.840344	68.063602	13635.968345	11946.439528	11327.949753
5	7.403354	7.829389	8.964297	0.000000	3.159123	1.862126	13	168	69	0	2	1	33.252589	71.246608	82.392782	0.000000	36.091962	11.215792
6	8.399087	10.231197	8.943278	11.443116	11.427158	11.411193	26	891	68	799	694	1033	66.505178	377.861474	81.198684	13896.860597	12523.910925	11585.912965
7	8.091982	7.155118	8.441998	0.000000	3.159123	0.000000	21	105	48	0	2	0	53.715721	44.529130	57.316718	0.000000	36.091962	0.000000
8	6.038414	6.950223	8.028343	5.015769	5.602801	3.528787	5	91	36	9	12	4	12.789457	38.591913	42.987538	156.535351	216.551774	44.863167
9	7.948148	8.021241	9.527263	0.000000	5.032606	0.000000	19	192	102	0	8	0	48.599938	81.424695	121.798025	0.000000	144.367849	0.000000
10	8.091982	7.536538	8.833328	0.000000	3.159123	1.862126	21	137	63	0	2	1	53.715721	58.099912	75.228192	0.000000	36.091962	11.215792
11	5.721963	5.946159	4.502920	0.000000	0.000000	0.000000	4	45	3	0	0	0	10.231566	19.083913	3.582295	0.000000	0.000000	0.000000
12	8.091982	7.247594	8.878318	0.000000	3.159123	0.000000	21	112	65	0	2	0	53.715721	47.497739	77.616389	0.000000	36.091962	0.000000
13	12.422550	13.375379	12.351009	7.536260	7.852081	7.708732	424	7883	723	53	58	79	1084.545987	3343.077438	863.333063	921.819289	1046.666907	886.047555
14	8.091982	7.998609	8.921948	0.000000	3.159123	0.000000	21	189	67	0	2	0	53.715721	80.152434	80.004585	0.000000	36.091962	0.000000
15	3.801717	8.929531	3.949426	10.681009	10.678721	10.665714	1	361	2	471	413	616	2.557891	153.095389	2.388197	8192.016697	7452.990219	6908.927770
16	10.250141	8.516808	10.393114	0.000000	0.000000	4.281551	94	271	186	0	0	7	240.441799	114.927564	222.102282	0.000000	0.000000	78.510543
17	8.399087	10.296049	8.943278	11.443116	11.427158	11.411193	26	932	68	799	694	1033	66.505178	395.249039	81.198684	13896.860597	12523.910925	11585.912965
18	9.156329	9.262788	10.102122	4.203014	3.689221	2.648663	44	455	152	5	3	2	112.547225	192.959563	181.502940	86.964084	54.137943	22.431584
19	10.865066	9.230779	11.283913	0.000000	0.000000	4.627541	144	445	345	0	0	9	368.336373	188.718693	411.963910	0.000000	0.000000	100.942127
20	15.287327	11.719432	15.869366	6.212452	6.327843	9.796267	3089	2501	8286	21	20	337	7901.326778	1060.641465	9894.298419	365.249152	360.919623	3779.721848
21	3.801717	8.563815	3.949426	10.477516	10.500591	10.446197	1	280	2	409	365	529	2.557891	118.744346	2.388197	7113.662058	6586.783123	5933.153881
22	11.577482	11.014495	12.616324	5.163306	5.715993	6.323062	236	1534	869	10	13	30	603.662389	650.549383	1037.671413	173.928168	234.597755	336.473755
23	4.749040	6.282261	7.946206	0.000000	0.000000	0.000000	2	57	34	0	0	0	5.115783	24.172956	40.599342	0.000000	0.000000	0.000000
24	12.408878	7.608053	12.900194	0.000000	0.000000	0.000000	420	144	1058	0	0	0	1074.314421	61.068521	1263.355989	0.000000	0.000000	0.000000
25	3.801717	6.378892	5.214206	8.296994	7.876637	8.089844	1	61	5	90	59	103	2.557891	25.869304	5.970491	1565.353509	1064.712888	1155.226559
26	10.952483	7.546974	11.096714	0.000000	0.000000	0.000000	153	138	303	0	0	0	391.357396	58.523999	361.811781	0.000000	0.000000	0.000000
27	8.399087	10.232814	8.943278	11.443116	11.427158	11.411193	26	892	68	799	694	1033	66.505178	378.285561	81.198684	13896.860597	12523.910925	11585.912965
28	15.039815	10.696027	15.606545	5.912168	6.096544	9.661915	2602	1230	6906	17	17	307	6655.633628	521.626950	8246.442781	295.677885	306.781680	3443.248093
29	11.874660	8.389220	12.343007	0.000000	0.000000	0.000000	290	248	719	0	0	0	741.788529	105.173564	858.556670	0.000000	0.000000	0.000000
30	10.998853	7.998609	11.096714	0.000000	0.000000	0.000000	158	189	303	0	0	0	404.146854	80.152434	361.811781	0.000000	0.000000	0.000000
31	6.297785	7.685666	8.584565	3.900537	2.312210	0.000000	6	152	53	4	1	0	15.347349	64.461217	63.287209	69.571267	18.045981	0.000000
32	0.000000	7.358396	7.184760	0.000000	0.000000	2.648663	0	121	20	0	0	2	0.000000	51.314521	23.881966	0.000000	0.000000	22.431584
33	7.948148	8.136291	9.527263	0.000000	5.032606	0.000000	19	208	102	0	8	0	48.599938	88.210086	121.798025	0.000000	144.367849	0.000000
34	5.316007	6.533832	7.033858	0.000000	2.312210	0.000000	3	68	18	0	1	0	7.673674	28.837913	21.493769	0.000000	18.045981	0.000000
35	7.870470	6.765960	6.952044	0.000000	3.159123	0.000000	18	80	17	0	2	0	46.042047	33.926956	20.299671	0.000000	36.091962	0.000000
36	3.801717	6.178690	7.184760	0.000000	2.312210	0.000000	1	53	20	0	1	0	2.557891	22.476608	23.881966	0.000000	18.045981	0.000000
37	8.399087	10.272659	8.943278	11.443116	11.427158	11.411193	26	917	68	799	694	1033	66.505178	388.887734	81.198684	13896.860597	12523.910925	11585.912965
38	9.340450	8.619263	9.854441	7.096529	6.906807	7.240501	50	291	128	39	30	57	127.894574	123.409303	152.844581	678.319854	541.379435	639.300135
39	0.000000	8.440498	8.471661	4.203014	3.159123	3.825496	0	257	49	5	2	5	0.000000	108.990347	58.510816	86.964084	36.091962	56.078959
40	8.021857	7.085234	8.714301	0.000000	3.159123	0.000000	20	100	58	0	2	0	51.157830	42.408695	69.257701	0.000000	36.091962	0.000000
41	12.170413	7.928502	12.700156	0.000000	0.000000	0.000000	356	180	921	0	0	0	910.609366	76.335651	1099.764524	0.000000	0.000000	0.000000
42	8.742134	10.323624	8.689271	11.439502	11.408333	11.399981	33	950	57	797	685	1025	84.410419	402.882604	68.063602	13862.074963	12361.497095	11496.186631
43	13.093276	8.136291	13.777797	0.000000	0.000000	0.000000	675	208	1944	0	0	0	1726.576748	88.210086	2321.327073	0.000000	0.000000	0.000000
44	3.801717	8.755983	3.949426	10.408938	10.419349	10.384960	1	320	2	390	345	507	2.557891	135.707824	2.388197	6783.198539	6225.863500	5686.406460
45	3.801717	8.839010	3.949426	10.681009	10.675225	10.665714	1	339	2	471	412	616	2.557891	143.765477	2.388197	8192.016697	7434.944238	6908.927770
46	4.749040	5.224709	5.688486	0.000000	0.000000	0.000000	2	27	7	0	0	0	5.115783	11.450348	8.358688	0.000000	0.000000	0.000000
47	8.399087	9.990554	7.946206	0.000000	0.000000	2.648663	26	754	34	0	0	2	66.505178	319.761561	40.599342	0.000000	0.000000	22.431584
48	15.299882	9.471667	16.849897	4.665885	3.159123	5.340970	3116	526	16350	7	2	15	7970.389848	223.069736	19523.507019	121.749717	36.091962	168.236878
49	4.749040	5.463780	5.688486	0.000000	0.000000	0.000000	2	32	7	0	0	0	5.115783	13.570782	8.358688	0.000000	0.000000	0.000000
50	7.701316	7.272963	8.810295	0.000000	0.000000	0.000000	16	114	62	0	0	0	40.926264	48.345912	74.034094	0.000000	0.000000	0.000000
51	9.311349	7.904354	9.622897	7.021150	6.906807	7.240501	49	177	109	37	30	57	125.336682	75.063390	130.156713	643.534220	541.379435	639.300135
52	0.000000	11.082432	8.529217	5.297145	4.076023	0.000000	0	1608	51	11	4	0	0.000000	681.931818	60.899013	191.320984	72.183925	0.000000
53																		
54	12.190531	8.742426	12.714183	0.000000	0.000000	0.000000	361	317	930	0	0	0	923.398824	134.435564	1110.511408	0.000000	0.000000	0.000000
55	8.652154	8.755983	9.230325	0.000000	3.159123	0.000000	31	320	83	0	2	0	79.294636	135.707824	99.110158	0.000000	36.091962	0.000000
56	9.905326	7.168697	9.831745	0.000000	0.000000	0.000000	74	106	126	0	0	0	189.283969	44.953217	150.456384	0.000000	0.000000	0.000000
57	12.425948	13.356592	12.360950	7.363920	7.852081	7.671917	425	7781	728	47	58	77	1087.103879	3299.820569	869.303554	817.462388	1046.666907	863.615971
58	3.801717	5.372849	5.214206	8.296994	7.876637	8.089844	1	30	5	90	59	103	2.557891	12.722609	5.970491	1565.353509	1064.712888	1155.226559
59	6.038414	6.885908	4.502920	0.000000	0.000000	1.862126	5	87	3	0	0	1	12.789457	36.895565	3.582295	0.000000	0.000000	11.215792
60	12.508396	8.972748	13.017962	0.000000	0.000000	0.000000	450	372	1148	0	0	0	1151.051166	157.760346	1370.824835	0.000000	0.000000	0.000000
61	11.631460	8.293302	12.156978	0.000000	0.000000	0.000000	245	232	632	0	0	0	626.683412	98.388173	754.670118	0.000000	0.000000	0.000000
62	11.849576	7.647382	12.324838	0.000000	0.000000	0.000000	285	148	710	0	0	0	728.999071	62.764869	847.809785	0.000000	0.000000	0.000000
63	11.782239	11.091372	12.948461	5.297145	5.715993	6.583090	272	1618	1094	11	13	36	695.746482	686.172687	1306.343528	191.320984	234.597755	403.768506
64	12.398538	10.319065	13.080664	5.419615	5.032606	4.071469	417	947	1199	12	8	6	1066.640747	401.610343	1431.723848	208.713801	144.367849	67.294751
65	5.316007	7.494022	0.000000	2.164632	0.000000	0.000000	3	133	0	1	0	0	7.673674	56.403565	0.000000	17.392817	0.000000	0.000000
66																		
67	7.701316	7.617986	8.810295	0.000000	0.000000	0.000000	16	145	62	0	0	0	40.926264	61.492608	74.034094	0.000000	0.000000	0.000000
68	4.749040	5.224709	5.688486	0.000000	0.000000	0.000000	2	27	7	0	0	0	5.115783	11.450348	8.358688	0.000000	0.000000	0.000000
69	8.091982	7.322400	8.878318	0.000000	3.159123	0.000000	21	118	65	0	2	0	53.715721	50.042260	77.616389	0.000000	36.091962	0.000000
70	3.801717	8.980468	3.949426	10.681009	10.678721	10.665714	1	374	2	471	413	616	2.557891	158.608520	2.388197	8192.016697	7452.990219	6908.927770
71	8.021857	6.852643	8.714301	0.000000	3.159123	0.000000	20	85	58	0	2	0	51.157830	36.047391	69.257701	0.000000	36.091962	0.000000
72	7.701316	7.155118	8.786889	0.000000	0.000000	1.862126	16	105	61	0	0	1	40.926264	44.529130	72.839996	0.000000	0.000000	11.215792
73	12.906443	7.637650	13.446402	0.000000	0.000000	0.000000	593	147	1545	0	0	0	1516.829647	62.340782	1844.881856	0.000000	0.000000	0.000000
74																		
75	7.608668	7.427802	5.688486	9.873548	9.654969	9.694402	15	127	7	269	203	314	38.368372	53.859043	8.358688	4678.667710	3663.334175	3521.758636
76	7.701316	7.113595	8.500726	0.000000	0.000000	0.000000	16	102	50	0	0	0	40.926264	43.256869	59.704914	0.000000	0.000000	0.000000
77	4.749040	9.126784	3.949426	10.684067	10.682208	10.665714	2	414	2	472	414	616	5.115783	175.571998	2.388197	8209.409514	7471.036200	6908.927770
78	10.695237	9.221037	11.110922	0.000000	0.000000	4.627541	128	442	306	0	0	9	327.410109	187.446433	365.394076	0.000000	0.000000	100.942127
79	12.166356	7.759317	12.697020	0.000000	0.000000	0.000000	355	160	919	0	0	0	908.051475	67.853912	1097.376327	0.000000	0.000000	0.000000
80	8.399087	9.818176	7.946206	0.000000	0.000000	2.648663	26	669	34	0	0	2	66.505178	283.714170	40.599342	0.000000	0.000000	22.431584
81	3.801717	8.619263	3.949426	10.408938	10.415164	10.384960	1	291	2	390	344	507	2.557891	123.409303	2.388197	6783.198539	6207.817519	5686.406460
82	8.021857	7.041620	8.215296	0.000000	3.159123	0.000000	20	97	41	0	2	0	51.157830	41.136434	48.958030	0.000000	36.091962	0.000000
83	12.472698	8.813303	13.346875	3.517347	3.159123	5.598060	439	333	1442	3	2	18	1122.914359	141.220955	1721.889732	52.178450	36.091962	201.884253
84	7.788371	6.835718	5.470749	0.000000	0.000000	0.000000	17	84	6	0	0	0	43.484155	35.623304	7.164590	0.000000	0.000000	0.000000
85	4.749040	6.282261	7.946206	0.000000	0.000000	0.000000	2	57	34	0	0	0	5.115783	24.172956	40.599342	0.000000	0.000000	0.000000
86	7.608668	10.272659	8.349165	11.399133	11.402004	11.372997	15	917	45	775	682	1006	38.368372	388.887734	53.734423	13479.432994	12307.359151	11283.086586
87	12.170413	8.462716	12.714183	0.000000	0.000000	0.000000	356	261	930	0	0	0	910.609366	110.686694	1110.511408	0.000000	0.000000	0.000000
88	5.316007	6.885908	7.766413	0.000000	2.312210	0.000000	3	87	30	0	1	0	7.673674	36.895565	35.822949	0.000000	18.045981	0.000000
89	4.749040	5.275798	7.946206	0.000000	0.000000	0.000000	2	28	34	0	0	0	5.115783	11.874435	40.599342	0.000000	0.000000	0.000000
90	8.091982	7.472285	9.103434	0.000000	3.159123	0.000000	21	131	76	0	2	0	53.715721	55.555391	90.751470	0.000000	36.091962	0.000000
91	10.121927	7.928502	9.622897	2.164632	0.000000	4.281551	86	180	109	1	0	7	219.978667	76.335651	130.156713	17.392817	0.000000	78.510543
92	7.870470	6.711335	6.952044	0.000000	3.159123	0.000000	18	77	17	0	2	0	46.042047	32.654695	20.299671	0.000000	36.091962	0.000000
93	9.251325	9.181394	8.411713	0.000000	3.159123	0.000000	47	430	47	0	2	0	120.220900	182.357389	56.122620	0.000000	36.091962	0.000000
94	5.316007	1.231372	4.502920	0.000000	0.000000	0.000000	3	1	3	0	0	0	7.673674	0.424087	3.582295	0.000000	0.000000	0.000000
95	8.697845	10.070493	8.689271	11.415785	11.359079	11.378720	32	797	57	784	662	1010	81.852527	337.997300	68.063602	13635.968345	11946.439528	11327.949753
96	3.801717	7.285482	0.000000	2.164632	2.312210	0.000000	1	115	0	1	1	0	2.557891	48.769999	0.000000	17.392817	18.045981	0.000000
97	11.844506	7.439052	12.320769	0.000000	0.000000	0.000000	284	128	708	0	0	0	726.441180	54.283130	845.421588	0.000000	0.000000	0.000000
98	13.631119	9.943933	13.743251	8.109560	7.775800	8.955536	980	730	1898	79	55	188	2506.733649	309.583475	2266.398552	1374.032525	992.528964	2108.568865
99	11.874660	8.336053	12.343007	0.000000	0.000000	0.000000	290	239	719	0	0	0	741.788529	101.356781	858.556670	0.000000	0.000000	0.000000
100	0.000000	6.424884	5.688486	0.000000	0.000000	2.648663	0	63	7	0	0	2	0.000000	26.717478	8.358688	0.000000	0.000000	22.431584
101	4.749040	7.221772	8.215296	0.000000	2.312210	0.000000	2	110	41	0	1	0	5.115783	46.649565	48.958030	0.000000	18.045981	0.000000
102	6.297785	9.224291	7.987859	4.203014	5.345692	5.340970	6	443	35	5	10	15	15.347349	187.870519	41.793440	86.964084	180.459812	168.236878
103	7.870470	7.768264	9.527263	0.000000	4.076023	0.000000	18	161	102	0	4	0	46.042047	68.277999	121.798025	0.000000	72.183925	0.000000
104	3.801717	9.040787	3.949426	10.681009	10.682208	10.665714	1	390	2	471	414	616	2.557891	165.393911	2.388197	8192.016697	7471.036200	6908.927770

105	piR-bta-1513678	CCCGGCTAGCTCAGTCGGTAGAGCATGA	25:2627614-2627642:+	-119.146865	4.918139	0.00336883	0.005573883	4.826972	0.000000	3.801717	6.729774	3.949426	0.000000	0.000000	0.000000	1	78	2	0	0	0	2.557891	33.078782	2.388197	0.000000	0.000000	0.000000
106	piR-bta-618747	TTGGTGGTTCAGTGGTAGAATTCTCGCCTG	18:1416101-1416131:-	-12066.472563	11.237165	1.06086E-09	2.13306E-08	11.411463	0.000000	12.492279	8.724149	13.017962	0.000000	0.000000	0.000000	445	313	1148	0	0	0	1138.261708	132.739216	1370.824835	0.000000	0.000000	0.000000
107	piR-bta-5794028	GCTCAGTCGGTAGAGCATCAGACTTTTA	16:2248339-2248367:-	-83.631624	6.891167	8.93032E-07	2.85143E-06	7.759591	1.053041	8.021857	7.041620	8.215296	0.000000	3.159123	0.000000	20	97	41	0	2	0	51.157830	41.136434	48.958030	0.000000	36.091962	0.000000
108	piR-bta-11110299	GCTCAGTCGGTAGAGCATCAGACTTTTA	16:2248339-2248367:-	-58.116316	7.269310	6.50371E-07	2.12636E-06	8.027474	1.951284	8.222766	6.981337	8.878318	2.164632	3.689221	0.000000	23	93	65	1	3	0	58.831504	39.440086	77.616389	17.392817	54.137943	0.000000
109	piR-bta-1238702	CCAGGGTTCAAGTCCCTGTTCGGGCG	23:30029665-30029691:-	-48.647781	5.339846	0.00035839	0.000721043	5.803568	0.620709	6.038414	6.869372	4.502920	0.000000	0.000000	1.862126	5	86	3	0	0	1	12.789457	36.471478	3.582295	0.000000	0.000000	11.215792
110	piR-bta-1370385	CCGGATAGCTCAGTCGGTAGAGCATCAGAC	23:30029706-30029736:-	-202.222855	8.125181	3.32701E-09	2.80152E-08	9.087697	1.053041	8.982624	8.933514	9.346952	0.000000	3.159123	0.000000	39	362	90	0	2	0	99.757768	153.519476	107.468846	0.000000	36.091962	0.000000
111	piR-bta-1329086	CCGGCTAGCTCAGTCGGTAGAGCATGAGAC	25:2627615-2627645:+	-750.273413	7.266736	3.02117E-10	2.11388E-08	8.151159	0.000000	7.788371	7.854813	8.810295	0.000000	0.000000	0.000000	17	171	62	0	0	0	43.484155	72.518869	74.034094	0.000000	0.000000	0.000000
112	piR-bta-705470	CTGGGTCGGGGTTTCGTACGTAGCAGAG	16:58623629-58623657:-	3.969064	10.756820	0.043411247	0.057252514	9.215238	11.427848	8.399087	10.261613	8.985014	11.443116	11.429235	11.411193	26	910	70	799	695	1033	66.505178	385.919126	83.586880	13896.860597	12541.956906	11585.912965
113	piR-bta-557968	CTGGGTCGGGGTTTCGTACGTAGCA	16:58623632-58623657:-	4.936205	10.689771	0.026506147	0.037397897	8.725325	11.418719	7.701316	10.125494	8.349165	11.430426	11.427158	11.398573	16	828	45	792	694	1024	40.926264	351.143996	53.734423	13775.110879	12523.910925	11484.970839
114	piR-bta-99766	TCCGTAGTGTAGTGGTTATCACGTTCGCCT	3:20991879-20991909:+	-162.384473	9.510308	2.68808E-07	9.65587E-07	10.296629	1.542514	10.672531	9.154348	11.063009	0.000000	0.000000	4.627541	126	422	296	0	0	9	322.294326	178.964693	353.453093	0.000000	0.000000	100.942127
115	piR-bta-16167771	AGCGCCGCTGGTGTAGTGGTATCATGCAA	11:68555305-68555334:-	-31.689149	16.542881	0.000958816	0.001792855	16.404583	12.425641	17.485945	13.262367	18.465437	11.707868	12.640913	12.928142	14180	7289	50101	960	1610	2957	36270.901171	3091.169789	59825.518358	16697.104096	29054.029668	33165.096456
116	piR-bta-5063054	CCGCGGCCCGGGTTCGATTCCCGGTCAGGG	NKLS02000508.1:10785-10815	-79.674446	12.730323	5.45311E-06	1.50374E-05	13.428078	7.152007	13.422419	12.296348	14.565468	6.570692	6.587881	8.297449	848	3731	3356	27	24	119	2169.091974	1582.268416	4007.393857	469.606053	433.103548	1334.679228
117	piR-bta-3045527	GTCGGGGTTTCGTACGTAGCAGAG	1:85343019-85343043:+	6.985921	7.772558	0.001155507	0.002124265	5.640838	8.502150	6.708231	5.000078	5.214206	8.734675	8.240548	8.531226	8	23	5	122	76	140	20.463132	9.754000	5.970491	2121.923646	1371.494568	1570.210857
118	piR-bta-125601	CGGGGTTTCGTACGTAGCAGAGCAGCTCC	1:85343021-85343050:+	5.927265	7.380343	0.001119434	0.002071901	5.082443	8.104490	3.801717	6.231405	5.214206	8.280926	7.970884	8.061660	1	55	5	89	63	101	2.557891	23.324782	5.970491	1547.960692	1136.896813	1132.794975
119	piR-bta-676695	TCGGGGTTTCGTACGTAGCAGAGCAGC	1:85343020-85343047:+	7.719044	7.327659	0.000119282	0.000266917	4.855081	8.087825	3.801717	5.549319	5.214206	8.296994	7.876637	8.089844	1	34	5	90	59	103	2.557891	14.418956	5.970491	1565.353509	1064.712888	1155.226559
120	piR-bta-3298838	TGGTGGTTCAGTGGTAGAATTCTCGCCTG	18:1416101-1416130:-	-11085.358628	11.114929	1.12673E-09	2.13306E-08	11.294931	0.000000	12.388123	8.614310	12.882360	0.000000	0.000000	0.000000	414	290	1045	0	0	0	1058.967072	122.985216	1247.832712	0.000000	0.000000	0.000000
121	piR-bta-3948742	TAGCACCATTTGAAATCAGTGTTA	16:75577096-75577120:+	-568.115506	6.911970	1.61718E-05	4.24509E-05	6.580222	0.000000	3.801717	9.073639	6.865309	0.000000	0.000000	0.000000	1	399	16	0	0	0	2.557891	169.210694	19.105573	0.000000	0.000000	0.000000
122			4:94636848-94636872:-																								
123	piR-bta-554673	GCCCGGATAGCTCAGTCGGTAGAGCATCAGAC	23:30029706-30029738:-	-218.797155	10.051924	4.01632E-09	2.98904E-08	10.848263	3.150628	10.834709	9.926054	11.784028	3.900537	3.689221	1.862126	141	721	488	4	3	1	360.662699	305.766692	582.719965	69.571267	54.137943	11.215792
124	piR-bta-19068973	CCACGCGGGAGGCCCGGGTTCGATTCCCG	3:8034328-8034357:+	-4.412340	8.512126	0.011913811	0.01806928	9.066204	7.056152	9.311349	8.150048	9.737216	7.021150	6.906807	7.240501	49	210	118	37	30	57	125.336682	89.058260	140.903598	643.534220	541.379435	639.300135
125	piR-bta-3463729	TGGTGGTTCAGTGGTAGAATTCTCGCC	23:30943697-30943724:+	-10878.026815	11.087615	3.71583E-09	2.89988E-08	11.043842	0.000000	12.384635	7.879796	12.867095	0.000000	0.000000	0.000000	413	174	1034	0	0	0	1056.409181	73.791130	1234.697630	0.000000	0.000000	0.000000
126	piR-bta-11140737	CGTGGGTTCGAATCCCACTTCTGACACCA		-16.850128	6.764032	0.007452246	0.011897445	5.234279	3.532951	6.876622	8.826214	0.000000	2.994044	4.076023	3.528787	9	336	0	2	4	4	23.021023	142.493216	0.000000	34.785634	72.183925	44.863167
127	piR-bta-1576687	ATTGGTGGTTCAGTGGTAGAATTCTCGC	18:1819427-1819455:+	-17305.196871	11.756873	6.03165E-09	3.50349E-08	11.424434	0.000000	12.982258	7.685666	13.605378	0.000000	0.000000	0.000000	625	152	1725	0	0	0	1598.682174	64.461217	2059.819548	0.000000	0.000000	0.000000
128	piR-bta-4960699	ACCTGCTTCTGGGTCGGGGTTTCGTACGTA	16:58623635-58623665:-	7.969544	9.658910	0.012480777	0.018721165	5.867956	10.478568	4.749040	8.905402	3.949426	10.481036	10.508469	10.446197	2	355	2	410	367	529	5.115783	150.550868	2.388197	7131.054874	6622.875086	5933.153881
129	piR-bta-1559158	GGTCGGGGTTTCGTACGTAGCAGAGCA	1:85343018-85343045:+	5.563199	9.002448	0.007961446	0.012599854	7.015823	9.740973	7.608668	7.750313	5.688486	9.873548	9.654969	9.694402	15	159	7	269	203	314	38.368372	67.429825	8.358688	4678.667710	3663.334175	3521.758636
130	piR-bta-2619143	ATCACATTGCCAGGGATTTCCATT		-445.580750	10.623536	3.5644E-10	2.13306E-08	11.419230	2.933944	10.834709	12.440103	10.982878	2.994044	3.159123	2.648663	141	4122	280	2	2	2	360.662699	1748.086414	334.347521	34.785634	36.091962	22.431584
131	piR-bta-1064604	GTCGGGGTTTCGTACGTAGCAGAGCAGCTC	1:85343019-85343049:+	4.174938	7.867231	0.010761418	0.016504871	6.274898	8.502150	6.708231	6.902258	5.214206	8.734675	8.240548	8.531226	8	88	5	122	76	140	20.463132	37.319652	5.970491	2121.923646	1371.494568	1570.210857
132	piR-bta-530876	TAGCTCAGTCGGTAGAGCATCAGACT	23:30029705-30029731:-	-109.686731	7.262739	6.13251E-08	2.55599E-07	8.076878	1.053041	8.091982	7.260334	8.878318	0.000000	3.159123	0.000000	21	113	65	0	2	0	53.715721	47.921826	77.616389	0.000000	36.091962	0.000000
133	piR-bta-335649	TCAGTGGTAGAATTCTCGCCTGCCA	5:23826392-23826417:+	-1670.242472	8.394799	4.33898E-09	3.01423E-08	8.973074	0.000000	9.905326	7.182150	9.831745	0.000000	0.000000	0.000000	74	107	126	0	0	0	189.283969	45.377304	150.456384	0.000000	0.000000	0.000000
134	piR-bta-4565814	TAGCTCAGTCGGTAGAGCATCAGACTTT	16:2248341-2248369:-	-119.643975	7.384428	4.3154E-08	1.82652E-07	8.156126	1.053041	8.091982	7.272963	9.103434	0.000000	3.159123	0.000000	21	114	76	0	2	0	53.715721	48.345912	90.751470	0.000000	36.091962	0.000000
135	piR-bta-1082424	GCCCGGCTAGCTCAGTCGGTAGAGCATGA	25:2627613-2627642:+	-81.741675	6.101975	8.93267E-05	0.000203218	6.661560	0.770737	5.316007	6.902258	7.766413	0.000000	2.312210	0.000000	3	88	30	0	1	0	7.673674	37.319652	35.822949	0.000000	18.045981	0.000000
136	piR-bta-1399267	GTTCAGTGGTAGAATTCTCGCCTGC	2:39839401-39839426:+	-3701.181509	9.535698	6.3718E-10	2.13306E-08	9.945607	0.000000	10.998853	7.741254	11.096714	0.000000	0.000000	0.000000	158	158	303	0	0	0	404.146854	67.005738	361.811781	0.000000	0.000000	0.000000
137	piR-bta-1563447	GGATAGCTCAGTCGGTAGAGCATCAGACT	16:2247973-2248002:+	-127.317163	7.464617	3.20066E-09	2.80152E-08	8.367004	1.053041	8.091982	8.087081	8.921948	0.000000	3.159123	0.000000	21	201	67	0	2	0	53.715721	85.241477	80.004585	0.000000	36.091962	0.000000
138	piR-bta-99282	TCGGGGTTTCGTACGTAGCAGAGCAGCTCC	ENSBTAG00000049095	5.683927	7.415893	0.001700123	0.003033553	5.131605	8.134211	3.801717	6.378892	5.214206	8.296994	8.015795	8.089844	1	61	5	90	65	103	2.557891	25.869304	5.970491	1565.353509	1172.988775	1155.226559
139	piR-bta-629516	GTGGTTCAGTGGTAGAATTCTCGCCTGC	19:27767599-27767627:+	-9813.777170	10.939382	1.28921E-09	2.13306E-08	11.162980	0.000000	12.190531	8.584225	12.714183	0.000000	0.000000	0.000000	361	284	930	0	0	0	923.398824	120.440694	1110.511408	0.000000	0.000000	0.000000
140	piR-bta-1569440	GTCGGGGTTTCGTACGTAGCAGAGCAGCT	1:85343019-85343048:+	4.668830	7.841648	0.005297933	0.008609141	6.165881	8.502150	6.708231	6.575207	5.214206	8.734675	8.240548	8.531226	8	70	5	122	76	140	20.463132	29.686087	5.970491	2121.923646	1371.494568	1570.210857
141	piR-bta-3509392	TGGTTCAGTGGTAGAATTCTCGCCTGCC	2:39839399-39839427:+	-7770.306898	10.603063	9.40289E-10	2.13306E-08	10.925445	0.000000	11.874660	8.558667	12.343007	0.000000	0.000000	0.000000	290	279	719	0	0	0	741.788529	118.320259	858.556670	0.000000	0.000000	0.000000
142	piR-bta-342563	GTTTCCGTAGTGTAGTGGTTATCACGTTCG	3:20957583-20957613:+	-166.747524	10.155834	7.62775E-08	3.10802E-07	10.869051	2.889302	11.202774	9.556753	11.847626	4.203014	0.000000	4.464893	182	558	510	5	0	8	465.536249	236.640519	608.990127	86.964084	0.000000	89.726335
143	piR-bta-3141308	CCCGGATAGCTCAGTCGGTAGAGCATCAGA	23:30029707-30029737:-	-249.911431	8.428262	2.95116E-09	2.80152E-08	9.367728	1.053041	9.424389	8.905402	9.773394	0.000000	3.159123	0.000000	53	355	121	0	2	0	135.568248	150.550868	144.485893	0.000000	36.091962	0.000000
144	piR-bta-7820414	AGGCCCCGGGTTCGATCCCCGGCATCTCCACCA	17:50880782-50880815:+	-61.465562	7.926328	3.36633E-05	8.39276E-05	7.856804	2.328206	4.749040	9.556753	9.264620	0.000000	3.159123	3.825496	2	558	85	0	2	5	5.115783	236.640519	101.498355	0.000000	36.091962	56.078959
145	piR-bta-4489718	TGGTGGTTCAGTGGTAGAATTCTCGC	3:8034299-8034325:+	-10223.931659	10.998210	8.07651E-09	4.26887E-08	10.856902	0.000000	12.279620	7.494022	12.797064	0.000000	0.000000	0.000000	384	133	985	0	0	0	982.230328	56.403565	1176.186814	0.000000	0.000000	0.000000
146	piR-bta-3490722	CTGCTTCTGGGTCGGGGTTTCGTACGTAG	16:58623634-58623663:-	8.204793	9.851983	0.018483203	0.026911544	5.622485	10.677329	3.801717	9.116311	3.949426	10.684067	10.682208	10.665714	1	411	2	472	414	616	2.557891	174.299737	2.388197	8209.409514	7471.036200	6908.927770
147	piR-bta-1959351	CCCCCCACTGCTAAATTTGACTGGTT	4:112160140-112160166:+	-52387.844267	19.278026	8.41042E-16	4.59209E-13	20.272886	4.366022	20.358718	20.360819	20.099122	2.994044	5.197626	4.906397	103865	998985	155466	2	9	11	###########	###########	###########	34.785634	162.413830	123.373710
148	piR-bta-4879717	TCCAAGCAGTTGACCCGGGTTCGA	19:27846601-27846625:+	-52.087100	3.924514	0.023843308	0.033962195	4.257056	0.000000	5.316007	2.952241	4.502920	0.000000	0.000000	0.000000	3	5	3	0	0	0	7.673674	2.120435	3.582295	0.000000	0.000000	0.000000
149	piR-bta-443453	GGTCGGGGTTTCGTACGTAGCAGAGC	1:85343018-85343044:+	5.563099	9.002448	0.007837923	0.012440424	7.015823	9.740973	7.608668	7.750313	5.688486	9.873548	9.654969	9.694402	15	159	7	269	203	314	38.368372	67.429825	8.358688	4678.667710	3663.334175	3521.758636
150	piR-bta-648866	GGTAGCGTGGCCGAGCGGTCTAAG	23:30293278-30293302:+	-43.438394	5.301071	0.002979901	0.004954715	5.483419	0.770737	3.801717	5.463780	7.184760	0.000000	2.312210	0.000000	1	32	20	0	1	0	2.557891	13.570782	23.881966	0.000000	18.045981	0.000000
151	piR-bta-781107	GGTAGCGTGGCCGAGCGGTCTAAGGC	23:30293278-30293304:+	-49.684713	5.474855	0.001635554	0.002956995	5.712690	0.770737	3.801717	6.151592	7.184760	0.000000	2.312210	0.000000	1	52	20	0	1	0	2.557891	22.052521	23.881966	0.000000	18.045981	0.000000
152	piR-bta-241774	GCGTTGGTGGTATAGTGGTGAGCATAGCTG	NKLS02000508.1:1690-1720:-	-18.616713	4.994089	0.020812483	0.030142217	4.983793	0.998015	5.316007	6.595458	3.039914	2.994044	0.000000	0.000000	3	71	1	2	0	0	7.673674	30.110174	1.194098	34.785634	0.000000	0.000000
153	piR-bta-45826	GGTGGTTCAGTGGTAGAATTCTCGCCT	3:20949168-20949195:-	-10850.284756	11.083953	2.97924E-09	2.80152E-08	11.081776	0.000000	12.377633	8.006192	12.861504	0.000000	0.000000	0.000000	411	190	1030	0	0	0	1051.293398	80.576521	1229.921237	0.000000	0.000000	0.000000
154	piR-bta-5773857	GGTCCAGGGTTCAAGTCCCTGTTCGGGCG	23:30029665-30029694:-	-73.991326	5.884527	0.000151744	0.00033274	6.109109	0.620709	6.038414	7.785994	4.502920	0.000000	0.000000	1.862126	5	163	3	0	0	1	12.789457	69.126173	3.582295	0.000000	0.000000	11.215792
155	piR-bta-11331067	AGGGTCCAGGGTTCAAGTCCCTGTTCGG	23:30029668-30029696:-	-62.386872	5.696973	0.011332397	0.017235345	3.902024	0.620709	3.801717	7.904354	0.000000	0.000000	0.000000	1.862126	1	177	0	0	0	1	2.557891	75.063390	0.000000	0.000000	0.000000	11.215792
156	piR-bta-1311267	TGGTGGTTCAGTGGTAGAATTCTCGCCTGC	18:1416100-1416130:-	-11165.135272	11.125271	9.43036E-10	2.13306E-08	11.337316	0.000000	12.405439	8.724149	12.882360	0.000000	0.000000	0.000000	419	313	1045	0	0	0	1071.756530	132.739216	1247.832712	0.000000	0.000000	0.000000
157	piR-bta-1439175	GCGCCGCTGGTGTAGTGGTATCATGCAAGATT	25:546240-546272:-	-32.946139	17.010582	0.000853273	0.0016233	16.865663	12.828295	17.935909	13.716079	18.945001	12.067669	13.050733	13.366482	19370	9983	69857	1232	2139	4007	49546.357946	4233.660036	83416.124148	21427.950257	38600.353701	44941.677882
158	piR-bta-2927170	GTCGGGGTTTCGTACGTAGCAGAGCAG1:85343019-85343046:+		5.326516	7.815590	0.002394298	0.004059896	6.024676	8.502150	6.708231	6.151592	5.214206	8.734675	8.240548	8.531226	8	52	5	122	76	140	20.463132	22.052521	5.970491	2121.923646	1371.494568	1570.210857
159	piR-bta-1414145	AGCTGGGGGATTAGCTCAAATGGT	11:72585331-72585355:-	-182.460629	9.255887	2.53494E-05	6.41755E-05	9.143504	1.460247	10.381949	5.914269	11.134294	0.000000	4.380741	0.000000	103	44	311	0	5	0	263.462822	18.659826	371.364568	0.000000	90.229906	0.000000
160	piR-bta-2459337	TTCCGGGTTCGAGTCCCGGCGGAGTCGCC		-503.200461	6.710060	3.074E-08	1.37574E-07	7.650324	0.000000	7.608668	7.483194	7.859109	0.000000	0.000000	0.000000	15	132	32	0	0	0	38.368372	55.979478	38.211145	0.000000	0.000000	0.000000
161	piR-bta-1272733	TCTGGGTCGGGGTTTCGTACGTAGCAGAG	16:58623629-58623658:-	3.905622	10.761503	0.046959102	0.061046833	9.228261	11.427848	8.399087	10.300681	8.985014	11.443116	11.429235	11.411193	26	935	70	799	695	1033	66.505178	396.521300	83.586880	13896.860597	12541.956906	11585.912965
162	piR-bta-3397	GGTAGTGTGGCCGAGCGGTCTAAGGC	10:26471894-26471920:-	-25.357593	4.697136	0.009464395	0.014680567	5.028868	0.770737	3.801717	5.814139	5.470749	0.000000	2.312210	0.000000	1	41	6	0	1	0	2.557891	17.387565	7.164590	0.000000	18.045981	0.000000
163	piR-bta-1542926	GCCCGGCTAGCTCAGTCGGTAGAGCA	3:45753112-45753138:+	-57.144950	5.638160	0.000367135	0.00073427	6.294566	0.770737	5.316007	6.533832	7.033858	0.000000	2.312210	0.000000	3	68	18	0	1	0	7.673674	28.837913	21.493769	0.000000	18.045981	0.000000
164	piR-bta-2354515	TAGCTTATCAGACTGATGTTGACG	19:10803478-10803502:+	-220.397351	8.101531	1.75353E-08	8.32546E-08	8.779252	0.882888	8.399087	9.992464	7.946206	0.000000	0.000000	2.648663	26	755	34	0	0	2	66.505178	320.185648	40.599342	0.000000	0.000000	22.431584
165	piR-bta-8574169	GTGGTTCAGTGGTAGAATTCTCGCCTGCCA	19:27767599-27767629:+	-9851.974840	10.945011	9.32795E-10	2.13306E-08	11.217224	0.000000	12.190531	8.746959	12.714183	0.000000	0.000000	0.000000	361	318	930	0	0	0	923.398824	134.859651	1110.511408	0.000000	0.000000	0.000000
166	piR-bta-1364554	TTCCGTAGTGTAGTGGTTATCACGTTCGCC	7:40353426-40353456:-	-213.940209	9.579903	3.63494E-08	1.61356E-07	10.310067	1.427184	10.844899	8.964986	11.120316	0.000000	0.000000	4.281551	142	370	308	0	0	7	363.220590	156.912172	367.782273	0.000000	0.000000	78.510543
167	piR-bta-869213	CACGCGGGAGACCGGGGTTCGATTCCCCGACGG	NKLS02000508.1:30960-30993	-4.842278	9.914997	0.022092003	0.031826475	10.229180	8.263998	9.019079	11.644269	10.024193	7.508929	8.836492	8.446574	40	2374	144	52	115	132	102.315659	1006.782423	171.950154	904.426472	2075.287833	1480.484522
168	piR-bta-7042188	CTAGACTGAAGCTCCTTGAGGTGT		-187.975427	8.022191	5.61128E-09	3.33017E-08	8.948144	1.053041	9.251325	9.181394	8.411713	0.000000	3.159123	0.000000	47	430	47	0	2	0	120.220900	182.357389	56.122620	0.000000	36.091962	0.000000
169	piR-bta-1664173	ACCGCCTGGGAATACCGGGTGCTGTAGGCTT	21:33002887-33002918:+	-23.796676	9.464022	0.000879082	0.0016551	9.749047	4.757536	9.251325	11.584103	8.411713	2.994044	7.207093	4.071469	47	2277	47	2	37	6	120.220900	965.645988	56.122620	34.785634	667.701303	67.294751
170	piR-bta-1048485	GCCCGGCTAGCTCAGTCGGTAGAG	25:2617430-2617454:+	-136.140747	5.010190	0.000631598	0.001227234	5.619517	0.000000	4.749040	5.779156	6.330354	0.000000	0.000000	0.000000	2	40	11	0	0	0	5.115783	16.963478	13.135081	0.000000	0.000000	0.000000
171	piR-bta-6221690	GGTTCAGTGGTAGAATTCTCGCCTGCC	NKLS02000508.1:13982-14009	-6742.537437	10.398813	9.63802E-10	2.13306E-08	10.754045	0.000000	11.631460	8.473697	12.156978	0.000000	0.000000	0.000000	245	263	632	0	0	0	626.683412	111.534868	754.670118	0.000000	0.000000	0.000000
172	piR-bta-8561308	GAAAGGTTGGTGGTTCGAGCCCACCCAGGG	3:20994960-20994990:+	-185.178717	8.804994	1.23337E-08	6.12202E-08	9.568094	1.935929	8.785104	9.297196	10.621982	0.000000	3.159123	2.648663	34	466	218	0	2	2	86.968310	197.624519	260.313427	0.000000	36.091962	22.431584
173	piR-bta-6774674	TCACCTGCCGAATCAACTAGCCCC	NKLS02002111.1:9239-9263:-	-16.541276	7.997193	0.000600246	0.00117048	8.354932	4.252653	7.288587	7.732137	10.044073	2.164632	4.846244	5.747081	12	157	146	1	7	20	30.694698	66.581651	174.338350	17.392817	126.321868	224.315837
174	piR-bta-7420587	GGTGGTTCAGTGGTAGAATTCTCGCCTGCC	NKLS02000508.1:4489-4519:+	-11129.194966	11.120648	7.4799E-10	2.13306E-08	11.373607	0.000000	12.395075	8.851693	12.874054	0.000000	0.000000	0.000000	416	342	1039	0	0	0	1064.082855	145.037737	1240.668122	0.000000	0.000000	0.000000
175	piR-bta-1672350	CGGATAGCTCAGTCGGTAGAGCATCAGACT	23:30029705-30029735:-	-173.429968	7.906685	4.78406E-09	3.07306E-08	8.822738	1.053041	8.453376	8.667886	9.346952	0.000000	3.159123	0.000000	27	301	90	0	2	0	69.063070	127.650172	107.468846	0.000000	36.091962	0.000000
176	piR-bta-6088910	CGCGGGAGGCCCGGGTTCGATTCCCGG	18:1819461-1819488:+	-4.257151	8.429420	0.015353691	0.02271847	8.946200	7.017255	9.311349	7.904354	9.622897	6.981930	6.906807	7.163028	49	177	109	36	30	54	125.336682	75.063390	130.156713	626.141404	541.379435	605.652759
177	piR-bta-645717	GGGTGGTTCAGTGGTAGAATTCTCGCC	13:21370303-21370330:-	-10878.031138	11.087615	3.76314E-09	2.89988E-08	11.043842	0.000000	12.384635	7.879796	12.867095	0.000000	0.000000	0.000000	413	174	1034	0	0	0	1056.409181	73.791130	1234.697630	0.000000	0.000000	0.000000
178			4:85606089-85606116:-																								
179	piR-bta-13320894	TCCCTGGTGGTCTAGTGGTTAGGATTTGGT	12:76975289-76975319:-	-75.702655	11.683006	8.16452E-06	2.18521E-05	11.770938	6.368195	12.711390	9.022207	13.579218	5.993298	6.177834	6.933452	518	385	1694	18	18	46	1324.987786	163.273476	2022.802501	313.070702	324.827661	515.926424
180			2:56475247-56475277:-																								
181	piR-bta-132729	CTTCTGGGTCGGGGTTTCGTACGTA	16:58623635-58623660:-	9.441231	9.631327	0.008503078	0.013302809	5.474601	10.476083	3.801717	8.672659	3.949426	10.477516	10.504536	10.446197	1	302	2	409	366	529	2.557891	128.074259	2.388197	7113.662058	6604.829105	5933.153881
182	piR-bta-1271698	TTCTCGCCTGCCACGCGGGAGGCCCGG	2:39839415-39839442:+	-96.138563	4.603265	0.002346126	0.004003078	5.220745	0.000000	4.749040	5.224709	5.688486	0.000000	0.000000	0.000000	2	27	7	0	0	0	5.115783	11.450348	8.358688	0.000000	0.000000	0.000000
183	piR-bta-18665608	GTTCAGTGGTAGAATTCTCGCCTGCCACGC	2:39839401-39839431:+	-3750.555682	9.555045	2.23151E-10	2.03067E-08	10.063261	0.000000	10.998853	8.094215	11.096714	0.000000	0.000000	0.000000	158	202	303	0	0	0	404.146854	85.665564	361.811781	0.000000	0.000000	0.000000
184	piR-bta-19437751	CCACTCCCACTGCTTCACTTGACCAGCCT	4:112179929-112179958:+	-180.252359	8.704818	2.1267E-07	7.89917E-07	9.104607	1.822345	7.701316	10.802210	8.810295	0.000000	2.312210	3.154825	16	1324	62	0	1	3	40.926264	561.491124	74.034094	0.000000	18.045981	33.647376
185	piR-bta-1193317	TTCTGGGTCGGGGTTTCGTACGTAGC	16:58623633-58623659:-	4.720188	10.672825	0.032983752	0.045477597	8.717664	11.390673	7.608668	10.195158	8.349165	11.399133	11.399887	11.372997	15	869	45	775	681	1006	38.368372	368.531561	53.734423	13479.432994	12289.313170	11283.086586
186	piR-bta-6315668	GCGGCCCGGGTTCGATTCCCGGTCAGGGAA	3:21204635-21204665:+	-87.714241	12.321087	2.01859E-06	5.8625E-06	13.093993	6.638357	12.968343	12.211558	14.102077	5.734786	6.527132	7.653151	619	3518	2434	15	23	76	1583.334825	1491.937895	2906.435234	260.892252	415.057567	852.400179
187	piR-bta-152946	TCCCTGGTGGTCTAGTGGTTAGGATTTGGC	19:38898437-38898467:+	-173.392791	13.005026	1.8142E-06	5.35434E-06	13.173712	6.519115	14.118614	10.541513	14.861008	6.278681	6.254787	7.023877	1374	1105	4119	22	19	49	3514.542892	468.616081	4918.490851	382.641969	342.873642	549.573800
188			3:24146632-24146662:-																								
189	piR-bta-7678707	CGGGGTTTCGTACGTAGCAGAGCA	1:85343021-85343045:+	8.903692	7.283450	7.69526E-05	0.000181104	4.710895	8.056562	3.801717	5.116761	5.214206	8.280926	7.827099	8.061660	1	25	5	89	57	101	2.557891	10.602174	5.970491	1547.960692	1028.620926	1132.794975
190	piR-bta-5825357	GCTCAGTCGGTAGAGCATCAGACTT	19:27761058-27761083:+	-82.936416	6.879459	9.33138E-07	2.96217E-06	7.739496	1.053041	8.021857	6.981337	8.215296	0.000000	3.159123	0.000000	20	93	41	0	2	0	51.157830	39.440086	48.958030	0.000000	36.091962	0.000000
191	piR-bta-364081	TTCAGTGGTAGAATTCTCGCCTGC	2:39839402-39839426:+	-1789.919514	8.493257	8.13118E-09	4.26887E-08	8.967885	0.000000	9.981197	6.918424	10.004034	0.000000	0.000000	0.000000	78	89	142	0	0	0	199.515535	37.743739	169.561957	0.000000	0.000000	0.000000
192	piR-bta-102173	GTCCAGGGTTCAAGTCCCTGTTCGGGCG	23:30029665-30029693:-	-56.308182	5.527912	0.000239227	0.000500452	5.916673	0.620709	6.038414	7.208685	4.502920	0.000000	0.000000	1.862126	5	109	3	0	0	1	12.789457	46.225478	3.582295	0.000000	0.000000	11.215792
193	piR-bta-1478256	TCGGGGTTTCGTACGTAGCAGAGCA	1:85343020-85343045:+	8.253125	7.319415	7.92305E-05	0.000185665	4.796257	8.087825	3.801717	5.372849	5.214206	8.296994	7.876637	8.089844	1	30	5	90	59	103	2.557891	12.722609	5.970491	1565.353509	1064.712888	1155.226559
194	piR-bta-698159	GGTGGTTCAGTGGTAGAATTCTCGCCTG	18:1819430-1819458:+	-11012.315017	11.105404	1.08491E-09	2.13306E-08	11.282003	0.000000	12.377633	8.594323	12.874054	0.000000	0.000000	0.000000	411	286	1039	0	0	0	1051.293398	121.288868	1240.668122	0.000000	0.000000	0.000000
195	piR-bta-1218773	TTCAGTGGTAGAATTCTCGCCTGCC	2:39839402-39839427:+	-1811.296183	8.510717	4.29479E-09	3.01423E-08	9.060237	0.000000	9.981197	7.195479	10.004034	0.000000	0.000000	0.000000	78	108	142	0	0	0	199.515535	45.801391	169.561957	0.000000	0.000000	0.000000
196	piR-bta-1198098	ATGGGTGGTTCAGTGGTAGAATTCTCGCCT	13:21370302-21370332:-	-16926.670436	11.724999	2.76752E-09	2.80152E-08	11.560233	0.000000	12.979948	8.156877	13.543873	0.000000	0.000000	0.000000	624	211	1653	0	0	0	1596.124283	89.482347	1973.844471	0.000000	0.000000	0.000000
197			4:85606088-85606118:-																								
198	piR-bta-4687013	CGGATAGCTCAGTCGGTAGAGCATCAGA	23:30029707-30029735:-	-159.360283	7.781229	1.22611E-09	2.13306E-08	8.695535	1.053041	8.399087	8.457193	9.230325	0.000000	3.159123	0.000000	26	260	83	0	2	0	66.505178	110.262607	99.110158	0.000000	36.091962	0.000000
199	piR-bta-297914	GTCGGGGTTTCGTACGTAGCAGAGCA	1:85343019-85343045:+	5.458521	7.811199	0.002077074	0.003623266	5.996514	8.502150	6.708231	6.067104	5.214206	8.734675	8.240548	8.531226	8	49	5	122	76	140	20.463132	20.780261	5.970491	2121.923646	1371.494568	1570.210857
200	piR-bta-7446036	CCTGCTTCTGGGTCGGGGTTTCGTACGTA	16:58623635-58623664:-	7.969028	9.658910	0.012379717	0.018620731	5.867956	10.478568	4.749040	8.905402	3.949426	10.481036	10.508469	10.446197	2	355	2	410	367	529	5.115783	150.550868	2.388197	7131.054874	6622.875086	5933.153881
201	piR-bta-396903	GGTAGCGTGGCCGAGCGGTCTAAGG	23:30293278-30293303:+	-45.323203	5.355894	0.002462753	0.004163043	5.564338	0.770737	3.801717	5.706537	7.184760	0.000000	2.312210	0.000000	1	38	20	0	1	0	2.557891	16.115304	23.881966	0.000000	18.045981	0.000000
202	piR-bta-3179555	GAGGGTCCAGGGTTCAAGTCCCTGTTCGGG	23:30029667-30029697:-	-67.563912	8.476927	3.66212E-07	1.26552E-06	9.346936	2.486313	9.311349	9.990554	8.738905	2.994044	0.000000	4.464893	49	754	59	2	0	8	125.336682	319.761561	70.451799	34.785634	0.000000	89.726335
203	piR-bta-757434	ATTGGTGGTTCAGTGGTAGAATTCTCGCC	28:40004078-40004107:+	-18320.997493	11.839136	4.44966E-09	3.0184E-08	11.590053	0.000000	13.047684	8.028707	13.693768	0.000000	0.000000	0.000000	654	193	1834	0	0	0	1672.861027	81.848782	2189.976261	0.000000	0.000000	0.000000
204	piR-bta-1489854	CACGCGGGAGACCGGGGTTCAATTCCCCGACGGG	5:62676670-62676704:+	-4.386567	10.559493	0.029323952	0.040844076	10.988925	9.114000	9.885717	12.087918	10.993141	8.848075	9.312042	9.181884	73	3229	282	132	160	220	186.726078	1369.376766	336.735717	2295.851813	2887.356986	2467.474204
205	piR-bta-136956	GGTCGGGGTTTCGTACGTAGCAGAGCAGC	1:85343018-85343047:+	5.270219	9.013849	0.010110674	0.01559443	7.067170	9.740973	7.608668	7.904354	5.688486	9.873548	9.654969	9.694402	15	177	7	269	203	314	38.368372	75.063390	8.358688	4678.667710	3663.334175	3521.758636
206	piR-bta-3614754	GTGGTTCAGTGGTAGAATTCTCGC	11:64380168-64380192:+	-8918.960530	10.801472	8.8647E-09	4.4816E-08	10.691395	0.000000	12.047855	7.405036	12.621295	0.000000	0.000000	0.000000	327	125	872	0	0	0	836.430514	53.010869	1041.253708	0.000000	0.000000	0.000000
207	piR-bta-1405544	GGGTGGTTCAGTGGTAGAATTCTCGCCT	13:21370302-21370330:-	-10923.320384	11.093621	3.00519E-09	2.80152E-08	11.098047	0.000000	12.388123	8.036134	12.869882	0.000000	0.000000	0.000000	414	194	1036	0	0	0	1058.967072	82.272869	1237.085827	0.000000	0.000000	0.000000
208			4:85606088-85606116:-																								
209	piR-bta-226198	TTGGTGGTTCAGTGGTAGAATTCTCGCCTGC	18:1416100-1416131:-	-12146.269312	11.246670	9.44694E-10	2.13306E-08	11.450857	0.000000	12.508396	8.826214	13.017962	0.000000	0.000000	0.000000	450	336	1148	0	0	0	1151.051166	142.493216	1370.824835	0.000000	0.000000	0.000000
210	piR-bta-1655741	TAGCTCAGTCGGTAGAGCATCAGAC	16:2247976-2248001:+	-107.462263	7.235847	1.17728E-07	4.76142E-07	8.023261	1.053041	8.091982	7.099485	8.878318	0.000000	3.159123	0.000000	21	101	65	0	2	0	53.715721	42.832782	77.616389	0.000000	36.091962	0.000000

211	piR-bta-370769	TGGGTCGGGGTTTCGTACGTAGCAGAG	16:58623629-58623656:-	4.298255	10.722736	0.036097319	0.048906045	9.083883	11.415939	8.399087	10.163292	8.689271	11.439502	11.408333	11.399981	26	850	57	797	685	1025	66.505178	360.473909	68.063602	13862.074963	12361.497095	11496.186631
212	piR-bta-881110	GCCCGGCTAGCTCAGTCGGTAGAGCATGAGA	25:2622396-2622427:+	-76.190246	7.675955	1.12678E-06	3.47584E-06	8.388666	1.358674	7.870470	7.768264	9.527263	0.000000	4.076023	0.000000	18	161	102	0	4	0	46.042047	68.277999	121.798025	0.000000	72.183925	0.000000
213	piR-bta-7704701	CCCGGATAGCTCAGTCGGTAGAGCATCAG	23:30029708-30029737:-	-247.827550	8.416426	3.18753E-09	2.80152E-08	9.354185	1.053041	9.424389	8.876730	9.761435	0.000000	3.159123	0.000000	53	348	120	0	2	0	135.568248	147.582259	143.291795	0.000000	36.091962	0.000000
214	piR-bta-418626	CCGGCTAGCTCAGTCGGTAGAGCATGAGA	25:2627615-2627644:+	-710.588102	7.190419	1.14549E-09	2.13306E-08	8.046487	0.000000	7.701316	7.627851	8.810295	0.000000	0.000000	0.000000	16	146	62	0	0	0	40.926264	61.916695	74.034094	0.000000	0.000000	0.000000
215	piR-bta-10940055	CAAGATTCCCATTCTTGCGACCCGGGT	25:546220-546247:-	-602.681509	6.973045	4.54564E-07	1.53205E-06	7.364447	0.000000	5.316007	8.527387	8.249946	0.000000	0.000000	0.000000	3	273	42	0	0	0	7.673674	115.775738	50.152128	0.000000	0.000000	0.000000
216	piR-bta-1023080	GGCTAGCTCAGTCGGTAGAGCATGAGACTC	25:2624996-2625026:+	-239.578718	7.476819	6.65617E-10	2.13306E-08	8.242295	0.620709	7.788371	7.657049	9.281466	0.000000	0.000000	1.862126	17	149	86	0	0	1	43.484155	63.188956	102.692453	0.000000	0.000000	11.215792
217	piR-bta-595968	GGTCGGGGTTTCGTACGTAGCAGA	1:85343018-85343042:+	6.438226	8.971923	0.005069762	0.008262955	6.742637	9.738601	7.608668	7.405036	5.214206	9.873548	9.647853	9.694402	15	125	5	269	202	314	38.368372	53.010869	5.970491	4678.667710	3645.288194	3521.758636
218	piR-bta-176121	GGTTCAGTGGTAGAATTCTCGCCT	NKLS02000508.1:23493-23517	-6488.280343	10.343221	4.61141E-09	3.03353E-08	10.410678	0.000000	11.601722	7.494022	12.136290	0.000000	0.000000	0.000000	240	133	623	0	0	0	613.893955	56.403565	743.923234	0.000000	0.000000	0.000000
219	piR-bta-18349213	CGCGGGAGGCCCGGGTTCGATTCCCGGCC	NKLS02000508.1:23521-23550	-8.605018	9.363831	0.000469397	0.000925237	10.118147	7.090842	9.845683	9.736188	10.772570	7.202691	6.906807	7.163028	71	632	242	42	30	54	181.610295	268.022953	288.971786	730.498304	541.379435	605.652759
220	piR-bta-1160321	TTCCCTGGTGGTCTAGTGGTTAGGATTCGGC	18:55869309-55869340:-	-217.904560	13.521263	1.86639E-06	5.47875E-06	13.658126	6.714596	14.671702	10.939244	15.363433	6.278681	6.756139	7.108967	2016	1456	5835	22	27	52	5156.709221	617.470601	6967.563514	382.641969	487.241491	583.221175
221	piR-bta-603892	GGGTCGGGGTTTCGTACGTAGCAG	16:58623631-58623655:-	4.750528	10.664814	0.020525036	0.029804973	8.960124	11.383801	8.342674	9.899831	8.637867	11.415785	11.356899	11.378720	25	708	55	784	661	1010	63.947287	300.253562	65.675406	13635.968345	11928.393547	11327.949753
222	piR-bta-1588523	TTCTGGGTCGGGGTTTCGTACGTAGCAGA	16:58623630-58623659:-	3.935519	10.758581	0.045883452	0.059790847	9.213320	11.427156	8.399087	10.297594	8.943278	11.443116	11.427158	11.411193	26	933	68	799	694	1033	66.505178	395.673126	81.198684	13896.860597	12523.910925	11585.912965
223	piR-bta-2242511	GTTTCCGTAGTGTAGTGGTTATCACATTCGCC	3:20978864-20978896:+	-100.152599	14.102310	0.000138541	0.000307493	14.305967	7.445521	15.302657	11.726335	15.888907	6.212452	6.327843	9.796267	3122	2513	8399	21	20	337	7985.737197	1065.730509	10029.231526	365.249152	360.919623	3779.721848
224	piR-bta-12700960	CTCAGTCGGTAGAGCATGAGACTCTTA	25:2617440-2617467:+	-122.283059	6.537069	1.67879E-07	6.40993E-07	7.417165	0.620709	7.608668	6.783718	7.859109	0.000000	0.000000	1.862126	15	81	32	0	0	1	38.368372	34.351043	38.211145	0.000000	0.000000	11.215792
225	piR-bta-18430398	AGCTGGGGGATTAGCTCAAATGGTAG	11:72585329-72585355:-	-189.640627	9.301141	3.56927E-06	1.00982E-05	9.625556	1.460247	10.395878	7.346497	11.134294	0.000000	4.380741	0.000000	104	120	311	0	5	0	266.020714	50.890434	371.364568	0.000000	90.229906	0.000000
226	piR-bta-1631464	GCATTGGTGGTTCAGTGGTAGAATTCTCGC	18:1423806-1423836:+	-44269.846473	13.111527	3.03648E-09	2.80152E-08	12.840939	0.000000	14.102779	9.330802	15.089236	0.000000	0.000000	0.000000	1359	477	4825	0	0	0	3476.174520	202.289476	5761.524243	0.000000	0.000000	0.000000
227	piR-bta-1679429	TGCTTCTGGGTCGGGGTTTCGTACGTA	16:58623635-58623662:-	8.700753	9.644357	0.011971195	0.018106019	5.518592	10.477394	3.801717	8.804632	3.949426	10.477516	10.508469	10.446197	1	331	2	409	367	529	2.557891	140.372781	2.388197	7113.662058	6622.875086	5933.153881
228	piR-bta-1140467	GCGTTGGTGGTATAGTGGTGAGCATAGCTGCCTT	19:27846568-27846602:+	-73.767554	6.679971	9.7475E-07	3.07638E-06	7.509391	0.998015	7.027397	7.285482	8.215296	2.994044	0.000000	0.000000	10	115	41	2	0	0	25.578915	48.769999	48.958030	34.785634	0.000000	0.000000
229	piR-bta-1480212	ATAGCTCAGTCGGTAGAGCATCAGACT	16:2248343-2248370:-	-111.848581	7.286918	2.09892E-08	9.79408E-08	8.121272	1.053041	8.091982	7.393517	8.878318	0.000000	3.159123	0.000000	21	124	65	0	2	0	53.715721	52.586782	77.616389	0.000000	36.091962	0.000000
230	piR-bta-35049	GCCCGGCTAGCTCAGTCGGTAGAGCATGAG	25:2617430-2617460:+	-76.161222	7.675955	1.23594E-06	3.79114E-06	8.388666	1.358674	7.870470	7.768264	9.527263	0.000000	4.076023	0.000000	18	161	102	0	4	0	46.042047	68.277999	121.798025	0.000000	72.183925	0.000000
231	piR-bta-19249886	GCCGTTCTTAGTTGGTGGAGTGATTT	16:59036779-59036805:+	-33.431795	6.109730	0.000875145	0.001653389	6.535166	1.172449	6.517555	5.059599	8.028343	3.517347	0.000000	0.000000	7	24	36	3	0	0	17.905240	10.178087	42.987538	52.178450	0.000000	0.000000
232	piR-bta-6286570	AGCTCAGTCGGTAGAGCATCAGACTTT	23:30111116-30111143:-	-93.205583	7.037496	2.0033E-07	7.54348E-07	7.900892	1.053041	8.091982	7.168697	8.441998	0.000000	3.159123	0.000000	21	106	48	0	2	0	53.715721	44.953217	57.316718	0.000000	36.091962	0.000000
233	piR-bta-1192485	TGGGTGGTTCAGTGGTAGAATTCTCGCC	13:21370303-21370331:-	-11718.843684	11.194903	4.47888E-09	3.0184E-08	11.146186	0.000000	12.505187	7.967870	12.965501	0.000000	0.000000	0.000000	449	185	1107	0	0	0	1148.493274	78.456086	1321.866806	0.000000	0.000000	0.000000
234																											
235	piR-bta-3862166	TGGTCTAGGGGTATGATTCTCGGT	15:55651415-55651439:-	-44.114696	6.300896	0.000236578	0.000496813	6.915977	1.051608	7.608668	5.372849	7.766413	0.000000	0.000000	3.154825	15	30	30	0	0	3	38.368372	12.722609	35.822949	0.000000	0.000000	33.647376
236	piR-bta-2494643	CAAAGAATTCTCCTTTTGGGCTTAA		-95.965556	6.343757	0.002695728	0.004542801	4.761926	0.721544	5.721963	8.563815	0.000000	2.164632	0.000000	0.000000	4	280	0	1	0	0	10.231566	118.744346	0.000000	17.392817	0.000000	0.000000
237	piR-bta-344087	GCATTGGTGGTTCAGTGGTAGAATTCTCG	3:21085363-21085392:+	-24547.531823	12.261110	2.85421E-09	2.80152E-08	12.122839	0.000000	13.192373	8.917517	14.258628	0.000000	0.000000	0.000000	723	358	2713	0	0	0	1849.355539	151.823129	3239.588657	0.000000	0.000000	0.000000
238	piR-bta-5795170	ATTCTCGCCTGCCACGCGGGAGGCCCGGGT	NKLS02000508.1:23507-23537	-25.773591	5.228772	0.001910495	0.003386786	5.703753	0.882888	4.749040	6.673734	5.688486	0.000000	0.000000	2.648663	2	75	7	0	0	2	5.115783	31.806521	8.358688	0.000000	0.000000	22.431584
239	piR-bta-1613459	GTCGGGGTTTCGTACGTAGCAGAGCAGC	1:85343019-85343047:+	5.161269	7.821422	0.002925564	0.004884887	6.059831	8.502150	6.708231	6.257057	5.214206	8.734675	8.240548	8.531226	8	56	5	122	76	140	20.463132	23.748869	5.970491	2121.923646	1371.494568	1570.210857
240	piR-bta-7889985	GGGGGTGTAGCTCAGTGGTAGAGAGCGTGCT	1:158044571-158044602:+	-22.282357	11.839551	0.000469007	0.000925237	12.083317	8.175782	13.366929	9.641975	13.241048	7.976212	7.580363	8.970772	816	592	1340	72	48	190	2087.239447	251.059475	1600.091707	1252.282807	866.207096	2131.000449
241	piR-bta-989719	TGGGTGGTTCAGTGGTAGAATTCTCGCCTG	13:21370301-21370331:-	-11929.476970	11.220680	1.15598E-09	2.13306E-08	11.390106	0.000000	12.508396	8.682159	12.979765	0.000000	0.000000	0.000000	450	304	1118	0	0	0	1151.051166	128.922433	1335.001887	0.000000	0.000000	0.000000
242			4:85606087-85606117:-																								
243	piR-bta-1341707	GTTTCCGTAGTGTAGTGGTTATCACGT	3:21038977-21039004:+	-40.491750	7.229956	1.25305E-06	3.82216E-06	8.094551	2.021713	8.158855	8.563815	7.560981	4.203014	0.000000	1.862126	22	280	26	5	0	1	56.273613	118.744346	31.046556	86.964084	0.000000	11.215792
244	piR-bta-288080	CTGCTTCTGGGTCGGGGTTTCGTACGTA	16:58623635-58623663:-	8.235021	9.653902	0.014818276	0.022105953	5.548109	10.478568	3.801717	8.893184	3.949426	10.481036	10.508469	10.446197	1	352	2	410	367	529	2.557891	149.278607	2.388197	7131.054874	6622.875086	5933.153881
245	piR-bta-1656107	GGAAACTCTGGTGGAGGTCCGTAGT	20:7977420-7977445:-	-10.607986	6.429651	0.001926754	0.003404556	7.031000	3.667923	6.038414	7.195479	7.859109	4.665885	3.689221	2.648663	5	108	32	7	3	2	12.789457	45.801391	38.211145	121.749717	54.137943	22.431584
246	piR-bta-1193100	ATGGGTGGTTCAGTGGTAGAATTCTCGCC	13:21370303-21370332:-	-16878.008759	11.720839	3.38646E-09	2.80152E-08	11.503582	0.000000	12.977634	7.990985	13.542127	0.000000	0.000000	0.000000	623	188	1651	0	0	0	1593.566391	79.728347	1971.456275	0.000000	0.000000	0.000000
247			4:85606089-85606118:-																								
248	piR-bta-1860430	GCCCGGCTAGCTCAGTCGGTAAAGCATG	25:2629352-2629380:+	-157.976966	5.175395	0.000767118	0.001464498	5.614166	0.000000	4.749040	5.059599	7.033858	0.000000	0.000000	0.000000	2	24	18	0	0	0	5.115783	10.178087	21.493769	0.000000	0.000000	0.000000
249	piR-bta-7179000	GGGTTTCGTACGTAGCAGAGCAGCTCCCTC	1:85343023-85343053:+	-14.430280	4.614511	0.038363731	0.05146584	3.560536	0.882888	0.000000	6.178690	4.502920	0.000000	0.000000	2.648663	0	53	3	0	0	2	0.000000	22.476608	3.582295	0.000000	0.000000	22.431584
250	piR-bta-1367952	TCCCTGGTGGTCTAGTGGTTAGGATTTGG	3:24146633-24146662:-	-69.290521	11.537583	1.13551E-05	2.99512E-05	11.641267	6.341151	12.524335	8.949336	13.450132	5.912168	6.177834	6.933452	455	366	1549	17	18	46	1163.840623	155.215824	1849.658249	295.677885	324.827661	515.926424
251	piR-bta-10937931	CTCAGTCGGTAGAGCATCAGACTT	23:31230724-31230748:+	-70.318622	6.655129	4.06784E-06	1.13485E-05	7.519394	1.053041	8.021857	6.818592	7.717732	0.000000	3.159123	0.000000	20	83	29	0	2	0	51.157830	35.199217	34.628850	0.000000	36.091962	0.000000
252	piR-bta-2355100	CCCCCCACTGCTAAATTTGACTGGCTA	4:112160140-112160167:+	-40647.080717	19.488743	2.39648E-15	6.54238E-13	20.483073	4.886727	20.642918	20.475542	20.330759	3.517347	5.197626	5.945207	126480	1081668	182543	3	9	23	###########	###########	###########	52.178450	162.413830	257.963212
253	piR-bta-7985248	GGGTCCAGGGTTCAAGTCCCTGTTCGGGCGCCA	15:82774044-82774077:-	-23.560902	9.739646	4.20303E-05	0.000102908	10.182222	5.964166	9.424389	11.791420	9.330857	6.768041	6.096544	5.027913	53	2629	89	31	17	12	135.568248	1114.924595	106.274748	539.177320	306.781680	134.589502
254	piR-bta-5112195	GCCTGCCACGCGGGAGGCCCGGGT	27:44779639-44779663:+	-15.868047	4.683252	0.028165433	0.039431606	3.834208	0.882888	0.000000	5.814139	5.688486	0.000000	0.000000	2.648663	0	41	7	0	0	2	0.000000	17.387565	8.358688	0.000000	0.000000	22.431584
255	piR-bta-16463784	GGGGGGTGTAGCTCAGTGGTAGAGCGCGTGCT	23:30156914-30156946:+	-27.669550	12.209368	0.00030202	0.000617614	12.445016	8.280299	13.632590	9.957687	13.744770	8.109560	7.775800	8.955536	981	737	1900	79	55	188	2509.291541	312.552083	2268.786748	1374.032525	992.528964	2108.568865
256	piR-bta-96015	CAGTCGGTAGAGCATCAGACTTTT	16:2248340-2248364:-	-49.462276	6.184288	0.000192917	0.000408267	6.922891	1.053041	7.870470	5.946159	6.952044	0.000000	3.159123	0.000000	18	45	17	0	2	0	46.042047	19.083913	20.299671	0.000000	36.091962	0.000000
257	piR-bta-3646297	TAGCTCAGTCGGTAGAGCATCAGACTTTT	23:30158665-30158694:+	-120.172376	7.390506	4.06813E-08	1.76286E-07	8.168538	1.053041	8.091982	7.310198	9.103434	0.000000	3.159123	0.000000	21	117	76	0	2	0	53.715721	49.618173	90.751470	0.000000	36.091962	0.000000
258	piR-bta-18775925	CCACGCGGGAGGCCCGGGTTCGATTCCCGG	NKLS02000508.1:23518-23548	-4.458979	8.524309	0.011249489	0.017157042	9.081075	7.056152	9.311349	8.170440	9.761435	7.021150	6.906807	7.240501	49	213	120	37	30	57	125.336682	90.330521	143.291795	643.534220	541.379435	639.300135
259	piR-bta-11092456	GGGGGGTGTAGCTCAGTGGTAGAGCGTG	23:30156914-30156942:+	-26.787222	12.040375	0.000346675	0.00070366	12.247904	8.125573	13.543111	9.699238	13.501363	7.996033	7.487756	8.892930	922	616	1605	73	45	180	2358.375944	261.237562	1916.527753	1269.675624	812.069152	2018.842530
260	piR-bta-3713941	AGCTCAGTCGGTAGAGCATCAGACTTTTA	23:31089841-31089870:+	-93.776268	7.045321	1.58296E-07	6.08659E-07	7.914222	1.053041	8.091982	7.208685	8.441998	0.000000	3.159123	0.000000	21	109	48	0	2	0	53.715721	46.225478	57.316718	0.000000	36.091962	0.000000
261	piR-bta-583674	AGGGTCCAGGGTTCAAGTCCCTGTTCGGGC	23:30029666-30029696:-	-183.248105	7.107526	3.09485E-07	1.09018E-06	7.395062	0.620709	6.038414	9.194730	6.952044	0.000000	0.000000	1.862126	5	434	17	0	0	1	12.789457	184.053737	20.299671	0.000000	0.000000	11.215792
262	piR-bta-1647637	GGCTCGTTGGTCTAGGGGTATGATTCT	25:2630254-2630281:+	-324.258904	6.103698	1.64865E-05	4.30699E-05	6.747639	0.000000	6.517555	5.779156	7.946206	0.000000	0.000000	0.000000	7	40	34	0	0	0	17.905240	16.963478	40.599342	0.000000	0.000000	0.000000
263	piR-bta-3417691	CACCGCCGCGGCCCGGGTTCGATTCCCGGT	9:25530065-25530095:+	-4.720836	6.863542	0.008431732	0.013267221	7.426645	5.274800	6.708231	7.904354	7.667351	5.637184	4.846244	5.340970	8	177	28	14	7	15	20.463132	75.063390	33.434752	243.499435	126.321868	168.236878
264	piR-bta-7838412	TCCCATATGGTCTAGCGGTTAGGATTCCTGC	12:11276342-11276373:-	-157.309627	10.398439	1.70913E-06	5.09938E-06	10.915636	2.977732	11.839419	8.988147	11.919343	0.000000	3.689221	5.243975	283	376	536	0	3	14	723.883289	159.456694	640.036683	0.000000	54.137943	157.021086
265	piR-bta-1478700	TTGGTGGTTCAGTGGTAGAATTCTCGC	3:21210207-21210234:+	-11197.145854	11.129279	6.78923E-09	3.74436E-08	10.996393	0.000000	12.391603	7.657049	12.940528	0.000000	0.000000	0.000000	415	149	1088	0	0	0	1061.524964	63.188956	1299.178938	0.000000	0.000000	0.000000
266	piR-bta-9025344	TCTCGCCTGCCACGCGGGAGGCCCGGG	3:8034319-8034346:+	-102.581058	4.680219	0.001677791	0.003013401	5.300436	0.000000	4.749040	5.463780	5.688486	0.000000	0.000000	0.000000	2	32	7	0	0	0	5.115783	13.570782	8.358688	0.000000	0.000000	0.000000
267	piR-bta-1690629	TTCTCGCCTGCCACGCGGGAGGCCCGGG	18:1819448-1819476:+	-102.582736	4.680219	0.001670384	0.00301	5.300436	0.000000	4.749040	5.463780	5.688486	0.000000	0.000000	0.000000	2	32	7	0	0	0	5.115783	13.570782	8.358688	0.000000	0.000000	0.000000
268	piR-bta-629150	AGCTCAGTCGGTAGAGCATCAGACT	23:30158666-30158691:+	-92.993266	7.034878	2.36518E-07	8.60927E-07	7.896366	1.053041	8.091982	7.155118	8.441998	0.000000	3.159123	0.000000	21	105	48	0	2	0	53.715721	44.529130	57.316718	0.000000	36.091962	0.000000
269	piR-bta-1663677	GCCCGGCTAGCTCAGTCGGTAGAGCATGG	10:70547109-70547138:-	-82.047417	6.107048	8.55034E-05	0.000197817	6.666948	0.770737	5.316007	6.918424	7.766413	0.000000	2.312210	0.000000	3	89	30	0	1	0	7.673674	37.743739	35.822949	0.000000	18.045981	0.000000
270	piR-bta-1110562	GTTTCCGTAGTGTAGTGGTTATCACGTTCGC	NKLS02001384.1:28640-28671	-100.857849	14.290156	0.000170073	0.000364157	14.233503	7.768607	15.498529	11.095821	16.106158	6.570692	6.808129	9.926998	3576	1623	9764	27	28	369	9147.019929	688.293122	11659.175690	469.606053	505.287472	4138.627187
271	piR-bta-3265202	AGCTGCCTTCCAAGCAGTTGACCCGGGT	19:27846593-27846621:+	-341.694599	6.196092	7.4078E-06	2.01227E-05	6.873720	0.000000	5.721963	7.577837	7.321362	0.000000	0.000000	0.000000	4	141	22	0	0	0	10.231566	59.796260	26.270162	0.000000	0.000000	0.000000
272	piR-bta-1286547	TCTGGGTCGGGGTTTCGTACGTAGCA	16:58623632-58623658:-	4.837913	10.694676	0.02904586	0.040560203	8.739617	11.418719	7.701316	10.168371	8.349165	11.430426	11.427158	11.398573	16	853	45	792	694	1024	40.926264	361.746170	53.734423	13775.110879	12523.910925	11484.970839
273	piR-bta-3710614	GCCGCGGCCCGGGTTCGATTCCCGGTCAGG	NKLS02000508.1:1278-1308:-	-75.819598	12.289331	4.07384E-06	1.13485E-05	12.932978	6.864360	13.208247	11.517343	14.073343	7.021150	5.918780	7.653151	731	2174	2386	37	15	76	1869.818671	921.965032	2849.118517	643.534220	270.689717	852.400179
274	piR-bta-296355	GCCCGGATAGCTCAGTCGGTAGAGCA	23:30029712-30029738:-	-93.038852	6.280956	0.000167152	0.00035931	6.576765	0.770737	4.749040	6.765960	8.215296	0.000000	2.312210	0.000000	2	80	41	0	1	0	5.115783	33.926956	48.958030	0.000000	18.045981	0.000000
275	piR-bta-518628	GTTTCCGTAGTGTAGTGGTTATCACGTTCGCC	NKLS02001384.1:28639-28671	-103.631123	14.440046	0.00011159	0.000250733	14.600426	7.871999	15.647864	11.923440	16.229975	6.622617	6.953724	10.039655	3966	2881	10639	28	31	399	10144.597605	1221.794507	12704.011693	486.998869	559.425416	4475.100942
276	piR-bta-3374235	TTCTCGCCTGCCACGCGGGAGGCCCGGGT	3:8034318-8034347:+	-24.979122	5.190292	0.00221801	0.003808282	5.677662	0.882888	4.749040	6.595458	5.688486	0.000000	0.000000	2.648663	2	71	7	0	0	2	5.115783	30.110174	8.358688	0.000000	0.000000	22.431584
277	piR-bta-10712221	GGGGTGTAGCTCAGTGGTAGAGAGCGTGCTT	1:158044572-158044603:+	-7.302198	7.202332	0.001285486	0.002342245	7.860472	4.863129	7.870470	7.099485	8.611462	4.203014	4.380741	6.005632	18	101	54	5	5	24	46.042047	42.832782	64.481308	86.964084	90.229906	269.179004
278	piR-bta-9577460	CTCAGTCGGTAGAGCATCAGACTTTTAAT	23:30029699-30029728:-	-71.041001	6.668962	3.56949E-06	1.00982E-05	7.541832	1.053041	8.021857	6.885908	7.717732	0.000000	3.159123	0.000000	20	87	29	0	2	0	51.157830	36.895565	34.628850	0.000000	36.091962	0.000000
279	piR-bta-162131	GGGTCGGGGTTTCGTACGTAGCAGAG	16:58623629-58623655:-	4.672504	10.669727	0.0238855	0.033962195	8.984665	11.384528	8.342674	9.922050	8.689271	11.415785	11.359079	11.378720	25	719	57	784	662	1010	63.947287	304.918518	68.063602	13635.968345	11946.439528	11327.949753
280	piR-bta-198649	CAGGGTTCAAGTCCCTGTTCGGGCG	23:30029665-30029690:-	-38.862903	5.067001	0.000666262	0.001289996	5.564779	0.620709	5.721963	6.469456	4.502920	0.000000	0.000000	1.862126	4	65	3	0	0	1	10.231566	27.565652	3.582295	0.000000	0.000000	11.215792
281	piR-bta-151186	CATGGGTGGTTCAGTGGTAGAATTCTCGCC	13:21370303-21370333:-	-17779.181626	11.795855	3.67117E-09	2.89988E-08	11.571434	0.000000	13.011954	8.058189	13.644157	0.000000	0.000000	0.000000	638	197	1772	0	0	0	1631.934764	83.545129	2115.942167	0.000000	0.000000	0.000000
282			4:85606089-85606119:-																								
283	piR-bta-111423	AGCATTGGTGGTTCAGTGGTAGAATTCTCGC	18:1416104-1416135:-	-44507.953376	13.119263	3.3476E-09	2.80152E-08	12.847252	0.000000	14.111246	9.333819	15.096692	0.000000	0.000000	0.000000	1367	478	4850	0	0	0	3496.637652	202.713563	5791.376700	0.000000	0.000000	0.000000
284	piR-bta-720907	GCCCGGATAGCTCAGTCGGTAGAGCATCAGA	23:30029707-30029738:-	-218.869775	9.900608	7.30094E-09	3.98631E-08	10.666706	2.529919	10.602186	9.708564	11.689369	3.900537	3.689221	0.000000	120	620	457	4	3	0	306.946977	262.933910	545.702918	69.571267	54.137943	0.000000
285	piR-bta-4626184	TAGCTGCCTTCCAAGCAGTTGACCCGGGT	19:27846592-27846621:+	-341.684068	6.196092	7.51539E-06	2.02901E-05	6.873720	0.000000	5.721963	7.577837	7.321362	0.000000	0.000000	0.000000	4	141	22	0	0	0	10.231566	59.796260	26.270162	0.000000	0.000000	0.000000
286	piR-bta-6271932	AGAGCCCGGATAGCTCAGTCGGTAGA	16:2247966-2247992:+	-266.094066	5.846799	0.000202939	0.000427817	6.081522	0.000000	4.749040	5.549319	7.946206	0.000000	0.000000	0.000000	2	34	34	0	0	0	5.115783	14.418956	40.599342	0.000000	0.000000	0.000000
287	piR-bta-1201837	TGGGTCGGGGTTTCGTACGTAGCA	16:58623632-58623656:-	5.077607	10.671146	0.023601341	0.033733854	8.699090	11.406756	7.701316	10.046789	8.349165	11.426780	11.406227	11.387262	16	784	45	790	684	1016	40.926264	332.484170	53.734423	13740.325246	12343.451113	11395.244504
288	piR-bta-4623028	CTCAGTCGGTAGAGCATGAGACTCT	25:2622406-2622431:+	-121.652528	6.529495	1.91772E-07	7.27137E-07	7.405252	0.620709	7.608668	6.747980	7.859109	0.000000	0.000000	1.862126	15	79	32	0	0	1	38.368372	33.502869	38.211145	0.000000	0.000000	11.215792
289	piR-bta-809791	ATAGCTCAGTCGGTAGAGCATCAGACTTTT	15:82772311-82772341:+	-122.195185	7.412558	2.46871E-08	1.1327E-07	8.211489	1.053041	8.091982	7.439052	9.103434	0.000000	3.159123	0.000000	21	128	76	0	2	0	53.715721	54.283130	90.751470	0.000000	36.091962	0.000000
290	piR-bta-1941672	CCCCTGGTGGTCTAGTGGTTAGGATTCGGC	NKLS02000508.1:10825-10855	-231.335746	13.521912	1.63309E-06	4.92634E-06	13.659153	6.639739	14.675276	10.939244	15.362938	6.278681	6.646175	6.994361	2021	1456	5833	22	25	48	5169.498679	617.470601	6965.175318	382.641969	451.149529	538.358008
291	piR-bta-993045	TGGGTCGGGGTTTCGTACGTAGCAGAGC	16:58623628-58623656:-	3.849590	10.754027	0.04981361	0.063845613	9.250664	11.415939	8.742134	10.320586	8.689271	11.439502	11.408333	11.399981	33	948	57	797	685	1025	84.410419	402.034430	68.063602	13862.074963	12361.497095	11496.186631
292	piR-bta-3344348	GGTGGTTCAGTGGTAGAATTCTCGCCTGC	3:8034300-8034329:+	-11092.118478	11.115814	9.851E-10	2.13306E-08	11.324922	0.000000	12.395075	8.705637	12.874054	0.000000	0.000000	0.000000	416	309	1039	0	0	0	1064.082855	131.042868	1240.668122	0.000000	0.000000	0.000000
293	piR-bta-312574	CATTGGTGGTTCAGTGGTAGAATTCTCGC	3:21085364-21085393:+	-18199.455535	11.829535	5.45775E-09	3.31103E-08	11.514575	0.000000	13.029930	7.820814	13.692981	0.000000	0.000000	0.000000	646	167	1833	0	0	0	1652.397895	70.822521	2188.782163	0.000000	0.000000	0.000000
294	piR-bta-1485189	CTGCTTCTGGGTCGGGGTTTCGTACGT	16:58623636-58623663:-	8.058137	9.584745	0.015345541	0.02271847	5.532874	10.405646	3.801717	8.847478	3.949426	10.412630	10.419349	10.384960	1	341	2	391	345	507	2.557891	144.613650	2.388197	6800.591356	6225.863500	5686.406460
295	piR-bta-1140096	CGGGGTTTCGTACGTAGCAGAGCAG	1:85343021-85343046:+	8.902997	7.283450	7.57007E-05	0.000178929	4.710895	8.056562	3.801717	5.116761	5.214206	8.280926	7.827099	8.061660	1	25	5	89	57	101	2.557891	10.602174	5.970491	1547.960692	1028.620926	1132.794975
296	piR-bta-5255300	CTGGGGATTGTGGGTTCGTGTCCCATCTGGGTC	19:48966370-48966403:+	-36.929886	8.830162	2.03191E-05	5.25793E-05	9.095885	4.597893	9.188695	7.195479	10.903483	4.665885	4.846244	4.281551	45	108	265	7	7	7	115.105117	45.801391	316.436046	121.749717	126.321868	78.510543
297	piR-bta-3573655	GCGCCGCTGGTGTAGTGGTATCATGCAA	11:68555305-68555333:-	-31.636119	16.533942	0.000964391	0.001797124	16.396216	12.419009	17.479930	13.253831	18.454888	11.701846	12.631926	12.923256	14121	7246	49736	956	1600	2947	36119.985573	3072.934050	59389.672483	16627.532829	28873.569856	33052.938537
298	piR-bta-18332917	AGCCCGGATAGCTCAGTCGGTAGAGCATC	23:30029710-30029739:-	-36.312369	6.896942	2.79433E-05	7.0309E-05	7.519513	2.070916	6.297785	7.676190	8.584565	3.900537	2.312210	0.000000	6	151	53	4	1	0	15.347349	64.037130	63.287209	69.571267	18.045981	0.000000
299	piR-bta-10222726	CTCAGTCGGTAGAGCATCAGACTTTT	23:31093149-31093175:-	-71.042176	6.668962	3.54804E-06	1.00982E-05	7.541832	1.053041	8.021857	6.885908	7.717732	0.000000	3.159123	0.000000	20	87	29	0	2	0	51.157830	36.895565	34.628850	0.000000	36.091962	0.000000
300	piR-bta-1045523	GCCCGGATAGCTCAGTCGGTAGAGCATC	23:30029710-30029738:-	-36.309216	6.896942	2.86775E-05	7.18252E-05	7.519513	2.070916	6.297785	7.676190	8.584565	3.900537	2.312210	0.000000	6	151	53	4	1	0	15.347349	64.037130	63.287209	69.571267	18.045981	0.000000
301	piR-bta-25439	TGGGTCGGGGTTTCGTACGTAGCAGA	16:58623630-58623656:-	4.360002	10.718194	0.034110208	0.046912276	9.061063	11.415236	8.399087	10.146234	8.637867	11.439502	11.406227	11.399981	26	840	55	797	684	1025	66.505178	356.233039	65.675406	13862.074963	12343.451113	11496.186631
302	piR-bta-880164	GCTTCTGGGTCGGGGTTTCGTACGTA	16:58623635-58623661:-	9.090653	9.636646	0.010160736	0.015627498	5.494820	10.476083	3.801717	8.733316	3.949426	10.477516	10.504536	10.446197	1	315	2	409	366	529	2.557891	133.587390	2.388197	7113.662058	6604.829105	5933.153881
303	piR-bta-1674654	GCATTGGTGGTTCAGTGGTAGAATTCTCGCC	3:21085363-21085394:+	-45736.003948	13.158523	3.03347E-09	2.80152E-08	12.945904	0.000000	14.146688	9.556753	15.134272	0.000000	0.000000	0.000000	1401	558	4978	0	0	0	3583.605962	236.640519	5944.221281	0.000000	0.000000	0.000000
304	piR-bta-1574246	GGGTCGGGGTTTCGTACGTAGCAGA	16:58623630-58623655:-	4.747018	10.665014	0.021432779	0.030958458	8.960802	11.383801	8.342674	9.901865	8.637867	11.415785	11.356899	11.378720	25	709	55	784	661	1010	63.947287	300.677649	65.675406	13635.968345	11928.393547	11327.949753
305	piR-bta-3772411	CGTAGTGTAGTGGTTATCACGTTCGCC	3:21024319-21024346:+	-127.704327	8.833455	1.65644E-06	4.96933E-06	9.465206	1.427184	10.187458	7.846388	10.361773	0.000000	0.000000	4.281551	90	170	182	0	0	7	230.210233	72.094782	217.325889	0.000000	0.000000	78.510543
306	piR-bta-476574	GGATAGCTCAGTCGGTAGAGCATCAGA	23:31557021-31557048:+	-113.438540	7.303386	6.38914E-09	3.59636E-08	8.199047	1.053041	8.021857	7.812187	8.763097	0.000000	3.159123	0.000000	20	166	60	0	2	0	51.157830	70.398434	71.645897	0.000000	36.091962	0.000000
307	piR-bta-5913531	TGCCTTCCAAGCAGTTGACCCGGGT	19:27846596-27846621:+	-283.837731	5.943214	2.1986E-05	5.66244E-05	6.669729	0.000000	5.721963	6.965864	7.321362	0.000000	0.000000	0.000000	4	92	22	0	0	0	10.231566	39.016000	26.270162	0.000000	0.000000	0.000000
308	piR-bta-3299038	GCGCCGCTGGTGTAGTGGTATCATG	11:68555308-68555333:-	-37.136204	11.341671	0.000144805	0.000320095	11.277827	7.008773	12.422550	8.216931	13.193998	6.278681	7.318835	7.428804	424	220	1297	22	40	65	1084.545987	93.299129	1548.745480	382.641969	721.839246	729.026469
309	piR-bta-447441	TTCCGTAGTGTAGTGGTTATCACGTTCGCCT	3:20978866-20978897:+	-180.748565	9.664066	1.50456E-07	5.82615E-07	10.416981	1.542514	10.844899	9.164550	11.241495	0.000000	0.000000	4.627541	142	425	335	0	0	9	363.220590	180.236954	400.022927	0.000000	0.000000	100.942127
310	piR-bta-33597	TGGGTGGTTCAGTGGTAGAATTCTCGCCT	13:21370302-21370331:-	-11767.475962	11.200893	3.25734E-09	2.80152E-08	11.204264	0.000000	12.508396	8.136291	12.968105	0.000000	0.000000	0.000000	450	208	1109	0	0	0	1151.051166	88.210086	1324.255002	0.000000	0.000000	0.000000
311			4:85606088-85606117:-																								
312	piR-bta-7737002	ATAGCTCAGTCGGTAGAGCATCAG	16:2247975-2247999:+	-96.610177	7.088999	2.96868E-07	1.05941E-06	7.876517	1.053041	8.021857	6.918424	8.689271	0.000000	3.159123	0.000000	20	89	57	0	2	0	51.157830	37.743739	68.063602	0.000000	36.091962	0.000000
313	piR-bta-1107312	GGTCGGGGTTTCGTACGTAGCAGAGCAG	1:85343018-85343046:+	5.445095	9.006892	0.008485746	0.013302809	7.036447	9.740973	7.608668	7.812187	5.688486	9.873548	9.654969	9.694402	15	166	7	269	203	314	38.368372	70.398434	8.358688	4678.667710	3663.334175	3521.758636
314	piR-bta-18444149	GAGCCCGGATAGCTCAGTCGGTAGAG	23:30029714-30029740:-	-293.307764	5.982678	8.60516E-05	0.000198245	6.325836	0.000000	4.749040	6.282261	7.946206	0.000000	0.000000	0.000000	2	57	34	0	0	0	5.115783	24.172956	40.599342	0.000000	0.000000	0.000000
315	piR-bta-1003918	ATTGGTGGTTCAGTGGTAGAATTCTCG	19:27767595-27767622:+	-8095.898880	10.662068	1.34746E-08	6.56886E-08	10.501649	0.000000	11.792806	7.155118	12.557024	0.000000	0.000000	0.000000	274	105	834	0	0	0	700.862265	44.529130	995.877973	0.000000	0.000000	0.000000
316	piR-bta-838845	TCGGGGTTTCGTACGTAGCAGAGCAG	1:85343020-85343046:+	8.114010	7.321480	8.67034E-05	0.000198908	4.811651	8.087825	3.801717	5.419031	5.214206	8.296994	7.876637	8.089844	1	31	5	90	59	103	2.557891	13.146695	5.970491	1565.353509	1064.712888	1155.226559

317	piR-bta-1158085	CAGTCGGTAGAGCATGAGACTCTT	26:17255704-17255728:+	-89.980440	6.105051	7.54374E-06	2.02901E-05	6.900908	0.620709	7.403354	5.914269	7.385100	0.000000	0.000000	1.862126	13	44	23	0	0	1	33.252589	18.659826	27.464261	0.000000	0.000000	11.215792
318	piR-bta-871409	TGGTTCAGTGGTAGAATTCTCGCCTG	23:31686626-31686652:+	-7659.073511	10.582241	1.55362E-09	2.29265E-08	10.820291	0.000000	11.849576	8.268291	12.343007	0.000000	0.000000	0.000000	285	228	719	0	0	0	728.999071	96.691825	858.556670	0.000000	0.000000	0.000000
319	piR-bta-11292936	GAGGGTCCAGGGTTCAAGTCCCTGTTCGG	23:30029668-30029697:-	-26.023289	7.163651	1.91567E-05	4.98075E-05	7.868412	2.486313	7.870470	8.782722	6.952044	2.994044	0.000000	4.464893	18	326	17	2	0	8	46.042047	138.252346	20.299671	34.785634	0.000000	89.726335
320	piR-bta-3672315	GCCTGGGTAGCTCAGTCGGTAGAGCATCAGA	23:31230714-31230745:+	-113.181297	7.302269	1.26248E-08	6.21005E-08	8.165280	1.053041	8.021857	7.617986	8.855998	0.000000	3.159123	0.000000	20	145	64	0	2	0	51.157830	61.492608	76.422290	0.000000	36.091962	0.000000
321	piR-bta-3045173	TGGTGGTTCAGTGGTAGAATTCTCG	3:8034299-8034324:+	-4513.856287	9.821053	3.0571E-09	2.80152E-08	9.881285	0.000000	11.078738	6.950223	11.614893	0.000000	0.000000	0.000000	167	91	434	0	0	0	427.167877	38.591913	518.238657	0.000000	0.000000	0.000000
322	piR-bta-15524745	CGTACCGTGAGTAATAATGCGAGG		-3147.540063	9.305757	3.41933E-12	6.22319E-10	10.261343	0.000000	10.577954	10.409056	9.797019	0.000000	0.000000	0.000000	118	1008	123	0	0	0	301.831195	427.479647	146.874090	0.000000	0.000000	0.000000
323	piR-bta-11663843	GGGTGGTTCAGTGGTAGAATTCTCGC	3:8100315-8100341:-	-10223.958009	10.998210	8.72276E-09	4.45105E-08	10.856902	0.000000	12.279620	7.494022	12.797064	0.000000	0.000000	0.000000	384	133	985	0	0	0	982.230328	56.403565	1176.186814	0.000000	0.000000	0.000000
324	piR-bta-371405	TGGTGGTTCAGTGGTAGAATTCTCGCCT	28:40004080-40004108:+	-10923.305050	11.093621	2.87261E-09	2.80152E-08	11.098047	0.000000	12.388123	8.036134	12.869882	0.000000	0.000000	0.000000	414	194	1036	0	0	0	1058.967072	82.272869	1237.085827	0.000000	0.000000	0.000000
325	piR-bta-19097173	GAGGGTCCAGGGTTCAAGTCCCTGTTCAG	19:25396376-25396405:+	-260.365538	5.846705	6.05375E-05	0.000146254	6.414227	0.000000	5.316007	7.472285	6.454390	0.000000	0.000000	0.000000	3	131	12	0	0	0	7.673674	55.555391	14.329179	0.000000	0.000000	0.000000
326	piR-bta-3428491	TCTCGCCTGCCACGCGGGAGGCCCGGGT	2:39839416-39839444:+	-24.977975	5.190292	0.002154667	0.003722938	5.677662	0.882888	4.749040	6.595458	5.688486	0.000000	0.000000	2.648663	2	71	7	0	0	2	5.115783	30.110174	8.358688	0.000000	0.000000	22.431584
327	piR-bta-160922	TCAGTCGGTAGAGCATGAGACTCTTAAT	25:2622407-2622435:+	-117.530889	6.480792	4.09044E-07	1.38719E-06	7.345481	0.620709	7.403354	6.729774	7.903314	0.000000	0.000000	1.862126	13	78	33	0	0	1	33.252589	33.078782	39.405244	0.000000	0.000000	11.215792
328	piR-bta-10717460	GACGAGGTGGCCGAGTGGTTAAGGCTATGG	23:30485859-30485889:-	-4.841434	6.523210	0.027867965	0.039115448	6.932177	4.715786	6.038414	6.729774	8.028343	5.015769	5.602801	3.528787	5	78	36	9	12	4	12.789457	33.078782	42.987538	156.535351	216.551774	44.863167
329	piR-bta-716988	TAGCTGCCTTCCAAGCAGTTGACCCGGG	19:27846592-27846620:+	-160.460301	5.230647	0.000519551	0.001020413	5.681158	0.000000	5.721963	6.818592	4.502920	0.000000	0.000000	0.000000	4	83	3	0	0	0	10.231566	35.199217	3.582295	0.000000	0.000000	0.000000
330	piR-bta-8850897	AGAGCCCGGATAGCTCAGTCGGTA	16:2247966-2247990:+	-230.856113	5.643044	0.001698771	0.003033553	5.123808	0.000000	4.749040	2.676178	7.946206	0.000000	0.000000	0.000000	2	4	34	0	0	0	5.115783	1.696348	40.599342	0.000000	0.000000	0.000000
331	piR-bta-280154	ATTGGTGGTTCAGTGGTAGAATTCTCGCCTG	18:1416101-1416132:-	-18560.335320	11.857889	1.98273E-09	2.77582E-08	11.831095	0.000000	13.049888	8.737878	13.705519	0.000000	0.000000	0.000000	655	316	1849	0	0	0	1675.418919	134.011477	2207.887736	0.000000	0.000000	0.000000
332	piR-bta-11298278	GTGCTCAGTAAATATTTGTGAAATGAATGA	21:14388748-14388778:-	139.137026	5.140976	0.000456998	0.000907349	0.000000	5.724849	0.000000	0.000000	0.000000	5.637184	6.397377	5.139985	0	0	0	14	21	13	0.000000	0.000000	0.000000	243.499435	378.965604	145.805294
333	piR-bta-4031143	TCCGTAGTGTAGTGGTTATCACGTTCGCCTG	3:21086763-21086794:-	-165.761617	9.540128	2.27378E-07	8.33212E-07	10.327786	1.542514	10.695237	9.191407	11.096714	0.000000	0.000000	4.627541	128	433	303	0	0	9	327.410109	183.629650	361.811781	0.000000	0.000000	100.942127
334	piR-bta-1421055	TAGCTCAGTCGGTAGAGCATGAGACTCT	25:2617437-2617465:+	-226.203557	7.393442	2.69892E-09	2.80152E-08	8.073735	0.620709	7.701316	7.221772	9.298119	0.000000	0.000000	1.862126	16	110	87	0	0	1	40.926264	46.649565	103.886551	0.000000	0.000000	11.215792
335	piR-bta-2687491	AGCTACATTGTCTGCTGGGTTTAT		-239.156199	5.754906	0.000588802	0.001152279	5.591136	0.000000	6.038414	7.695081	3.039914	0.000000	0.000000	0.000000	5	153	1	0	0	0	12.789457	64.885304	1.194098	0.000000	0.000000	0.000000
336	piR-bta-3365925	TAGCTCAGTCGGTAGAGCATCAGACTT	23:31557024-31557051:+	-118.609959	7.371921	4.14615E-08	1.78252E-07	8.145559	1.053041	8.091982	7.260334	9.084360	0.000000	3.159123	0.000000	21	113	75	0	2	0	53.715721	47.921826	89.557372	0.000000	36.091962	0.000000
337	piR-bta-1275560	GCATGGGTGGTTCAGTGGTAGAATTCTCGC	13:21370304-21370334:-	-40389.949474	12.979227	3.22548E-09	2.80152E-08	12.675178	0.000000	14.001656	9.080840	14.943040	0.000000	0.000000	0.000000	1267	401	4360	0	0	0	3240.848504	170.058868	5206.268538	0.000000	0.000000	0.000000
338	piR-bta-207526	TTGGTGGTTCAGTGGTAGAATTCTCGCC	18:1819428-1819456:+	-11851.316490	11.211135	3.9562E-09	2.98904E-08	11.166433	0.000000	12.489034	8.006192	13.004074	0.000000	0.000000	0.000000	444	190	1137	0	0	0	1135.703817	80.576521	1357.689754	0.000000	0.000000	0.000000
339	piR-bta-1970408	TCCCTGGTTCGATCCCGGGTTTCGGCACC	17:50876700-50876729:+	-346.547063	8.056967	3.77091E-09	2.89988E-08	8.873287	0.721544	8.091982	9.694551	8.833328	2.164632	0.000000	0.000000	21	614	63	1	0	0	53.715721	260.389388	75.228192	17.392817	0.000000	0.000000
340	piR-bta-853684	CTGGGTCGGGGTTTCGTACGTAGC	16:58623633-58623657:-	4.877122	10.664648	0.025399944	0.036021739	8.694442	11.390673	7.608668	10.125494	8.349165	11.399133	11.399887	11.372997	15	828	45	775	681	1006	38.368372	351.143996	53.734423	13479.432994	12289.313170	11283.086586
341	piR-bta-9878887	AGAGCCCGGATAGCTCAGTCGGTAGAGC	16:2247966-2247994:+	-93.053520	6.280956	0.000162688	0.00035249	6.576765	0.770737	4.749040	6.765960	8.215296	0.000000	2.312210	0.000000	2	80	41	0	1	0	5.115783	33.926956	48.958030	0.000000	18.045981	0.000000
342	piR-bta-7600638	TCAGTCGGTAGAGCATGAGACTCTTAATCT	6:709212-709242:-	-123.710276	6.554507	1.34008E-07	5.34075E-07	7.443800	0.620709	7.509660	6.918424	7.903314	0.000000	0.000000	1.862126	14	89	33	0	0	1	35.810481	37.743739	39.405244	0.000000	0.000000	11.215792
343	piR-bta-2044719	TCCGTAGTGTAGTGGTTATCACGTTCGCCTGA	3:21057897-21057929:+	-165.759801	9.540128	2.26626E-07	8.33212E-07	10.327786	1.542514	10.695237	9.191407	11.096714	0.000000	0.000000	4.627541	128	433	303	0	0	9	327.410109	183.629650	361.811781	0.000000	0.000000	100.942127
344	piR-bta-431127	TTGGTGGTTCAGTGGTAGAATTCTCGCCT	18:1423809-1423838:+	-11904.447505	11.217605	2.81671E-09	2.80152E-08	11.232026	0.000000	12.492279	8.197190	13.006609	0.000000	0.000000	0.000000	445	217	1139	0	0	0	1138.261708	92.026868	1360.077951	0.000000	0.000000	0.000000
345	piR-bta-588091	CGGATAGCTCAGTCGGTAGAGCATCAGAC	23:30029706-30029735:-	-171.643985	7.889915	2.71125E-09	2.80152E-08	8.803222	1.053041	8.453376	8.609339	9.346952	0.000000	3.159123	0.000000	27	289	90	0	2	0	69.063070	122.561129	107.468846	0.000000	36.091962	0.000000
346	piR-bta-778516	TCGGGGTTTCGTACGTAGCAGAGCAGCT	1:85343020-85343048:+	6.432075	7.354123	0.00044717	0.000891077	5.007940	8.087825	3.801717	6.007898	5.214206	8.296994	7.876637	8.089844	1	47	5	90	59	103	2.557891	19.932087	5.970491	1565.353509	1064.712888	1155.226559
347	piR-bta-6083947	GGGGGATGTAGCTCAGTGGTAGAGCGCGC	2:39839793-39839822:-	-10.966864	11.534814	0.004063996	0.006703752	11.665478	8.851134	13.045477	9.116311	12.834646	8.758079	8.240548	9.554776	653	411	1011	124	76	285	1670.303136	174.299737	1207.233370	2156.709279	1371.494568	3196.500673
348	piR-bta-3561575	AGGGTCCAGGGTTCAAGTCCCTGTTCGGG	23:30029667-30029696:-	-112.158141	6.448164	0.000763222	0.001462173	5.682354	0.620709	3.801717	8.742426	4.502920	0.000000	0.000000	1.862126	1	317	3	0	0	1	2.557891	134.435564	3.582295	0.000000	0.000000	11.215792
349	piR-bta-6743488	GCCCGGCTAGCTCAGTCGGTAGAGCATCA	25:2597666-2597695:-	-22.972966	6.293012	0.000349669	0.000707108	7.035515	2.070916	6.297785	7.141409	7.667351	3.900537	2.312210	0.000000	6	104	28	4	1	0	15.347349	44.105043	33.434752	69.571267	18.045981	0.000000
350	piR-bta-1569374	GGGTCGGGGTTTCGTACGTAGCAGAGCAG	16:58623626-58623655:-	4.157783	10.700809	0.037268304	0.050243194	9.160310	11.384528	8.697845	10.093813	8.689271	11.415785	11.359079	11.378720	32	810	57	784	662	1010	81.852527	343.510431	68.063602	13635.968345	11946.439528	11327.949753
351	piR-bta-1566457	TCTGGGTCGGGGTTTCGTACGTAG	16:58623634-58623658:-	10.192752	9.817666	0.007305738	0.011697751	5.506853	10.673983	3.801717	8.769415	3.949426	10.681009	10.675225	10.665714	1	323	2	471	412	616	2.557891	136.980085	2.388197	8192.016697	7434.944238	6908.927770
352	piR-bta-696277	GCATGGGTGGTTCAGTGGTAGAATT	13:21370309-21370334:-	-527.394160	6.763784	8.85326E-07	2.84346E-06	7.344234	0.000000	8.021857	5.630069	8.380778	0.000000	0.000000	0.000000	20	36	46	0	0	0	51.157830	15.267130	54.928521	0.000000	0.000000	0.000000
353	piR-bta-1101884	CGCCTGCCACGCGGGAGGCCCGGGT	NKLS02000508.1:14001-14026	-20.259158	4.962957	0.01760086	0.025695373	4.037790	0.882888	0.000000	6.424884	5.688486	0.000000	0.000000	2.648663	0	63	7	0	0	2	0.000000	26.717478	8.358688	0.000000	0.000000	22.431584
354	piR-bta-1529063	TCTGGGTCGGGGTTTCGTACGTAGCAG	16:58623631-58623658:-	3.979213	10.755391	0.04378655	0.057562278	9.204484	11.427156	8.399087	10.271086	8.943278	11.443116	11.427158	11.411193	26	916	68	799	694	1033	66.505178	388.463647	81.198684	13896.860597	12523.910925	11585.912965
355	piR-bta-11053063	TAGCTCAGTCGGTAGAGCATCAGACTTTTA	23:31089840-31089870:+	-120.179592	7.390506	3.9657E-08	1.73222E-07	8.168538	1.053041	8.091982	7.310198	9.103434	0.000000	3.159123	0.000000	21	117	76	0	2	0	53.715721	49.618173	90.751470	0.000000	36.091962	0.000000
356	piR-bta-770607	GGGTCGGGGTTTCGTACGTAGCAGAGCA	16:58623627-58623655:-	4.193186	10.698467	0.035791272	0.048733254	9.153139	11.384528	8.697845	10.072300	8.689271	11.415785	11.359079	11.378720	32	798	57	784	662	1010	81.852527	338.421387	68.063602	13635.968345	11946.439528	11327.949753
357	piR-bta-3791326	CCCGGCTAGCTCAGTCGGTAGAGCATGAGA	25:2638931-2638961:+	-73.736082	7.233323	3.19441E-07	1.11804E-06	8.070278	1.229740	7.701316	7.676190	8.833328	0.000000	3.689221	0.000000	16	151	63	0	3	0	40.926264	64.037130	75.228192	0.000000	54.137943	0.000000
358	piR-bta-501270	AGCTCAGTCGGTAGAGCATGAGAC	25:2625000-2625024:+	-570.148450	6.880860	4.29523E-08	1.82652E-07	7.701433	0.000000	7.701316	6.902258	8.500726	0.000000	0.000000	0.000000	16	88	50	0	0	0	40.926264	37.319652	59.704914	0.000000	0.000000	0.000000
359	piR-bta-951703	TGCCGTGATCGTATAGTGGTTAGTACT	8:29729056-29729083:+	-660.052035	7.096801	3.73024E-08	1.64251E-07	7.677659	0.000000	6.708231	9.003384	7.321362	0.000000	0.000000	0.000000	8	380	22	0	0	0	20.463132	161.153042	26.270162	0.000000	0.000000	0.000000
360	piR-bta-1315155	CTGGGAATACCGGGTGCTGTAGGCTT	NKLS02002061.1:3377-3403:+	-26.942031	9.239411	0.002216375	0.003808282	9.398668	3.561269	8.505697	11.440363	8.249946	0.000000	6.858311	3.825496	28	2061	42	0	29	5	71.620961	874.043207	50.152128	0.000000	523.333454	56.078959
361	piR-bta-718070	TCAGTCGGTAGAGCATCAGACTTTT	19:27761060-27761085:+	-55.812667	6.347626	3.99814E-05	9.83327E-05	7.196158	1.053041	7.870470	6.765960	6.952044	0.000000	3.159123	0.000000	18	80	17	0	2	0	46.042047	33.926956	20.299671	0.000000	36.091962	0.000000
362	piR-bta-561316	CATGGGTGGTTCAGTGGTAGAATTCTCGC	13:21370304-21370333:-	-16764.624092	11.711121	4.78108E-09	3.07306E-08	11.406582	0.000000	12.942478	7.722962	13.554308	0.000000	0.000000	0.000000	608	156	1665	0	0	0	1555.198019	66.157564	1988.173651	0.000000	0.000000	0.000000
363			4:85606090-85606119:-																								
364	piR-bta-9740736	AGCTGGGGGATTAGCTCAAATGGTAGA	11:72585328-72585355:-	-140.039295	9.303822	1.12586E-06	3.47584E-06	9.625556	2.458262	10.395878	7.346497	11.134294	2.994044	4.380741	0.000000	104	120	311	2	5	0	266.020714	50.890434	371.364568	34.785634	90.229906	0.000000
365	piR-bta-1572299	TCCCTGGTGGTCTAGTGGTTAGGATTCGGCAC	4:24092581-24092613:-	-257.079974	14.116203	1.90954E-06	5.57545E-06	14.145508	7.092926	15.280305	11.190388	15.965832	6.768041	7.245310	7.265427	3074	1733	8859	31	38	58	7862.958406	734.942687	10578.516739	539.177320	685.747284	650.515926
366	piR-bta-716356	CGGGGTTTCGTACGTAGCAGAGCAGCTC	1:85343021-85343049:+	5.745786	7.345663	0.001152737	0.002124265	5.082443	8.056562	3.801717	6.231405	5.214206	8.280926	7.827099	8.061660	1	55	5	89	57	101	2.557891	23.324782	5.970491	1547.960692	1028.620926	1132.794975
367	piR-bta-1574806	CTTCTGGGTCGGGGTTTCGTACGTAGCA	16:58623632-58623660:-	4.651364	10.705202	0.034712523	0.047501348	8.767519	11.419411	7.701316	10.252077	8.349165	11.430426	11.429235	11.398573	16	904	45	792	695	1024	40.926264	383.374604	53.734423	13775.110879	12541.956906	11484.970839
368	piR-bta-2382982	CAAAGTGCTTACAGTGCAGGTAGA	12:65689429-65689453:+	-559.843912	6.888438	7.16321E-06	1.95556E-05	6.823874	0.000000	4.749040	9.048152	6.674430	0.000000	0.000000	0.000000	2	392	14	0	0	0	5.115783	166.242085	16.717376	0.000000	0.000000	0.000000
369	piR-bta-58402	TGCTTCTGGGTCGGGGTTTCGTACGTAGC	16:58623633-58623662:-	4.482895	10.687441	0.039518294	0.052626802	8.753865	11.392082	7.608668	10.303762	8.349165	11.399133	11.404117	11.372997	15	937	45	775	683	1006	38.368372	397.369473	53.734423	13479.432994	12325.405132	11283.086586
370	piR-bta-10998566	ATTCTCGCCTGCCACGCGGGAGGCCCGG	1:96512977-96513005:+	-98.720922	4.634668	0.002005542	0.003520983	5.254222	0.000000	4.749040	5.325139	5.688486	0.000000	0.000000	0.000000	2	29	7	0	0	0	5.115783	12.298522	8.358688	0.000000	0.000000	0.000000
371	piR-bta-674521	GCTTCTGGGTCGGGGTTTCGTACGTAGCA	16:58623632-58623661:-	4.605850	10.707727	0.03694831	0.049935092	8.774380	11.419411	7.701316	10.272659	8.349165	11.430426	11.429235	11.398573	16	917	45	792	695	1024	40.926264	388.887734	53.734423	13775.110879	12541.956906	11484.970839
372	piR-bta-479953	CGGGGTTTCGTACGTAGCAGAGCAGC	1:85343021-85343048:+	6.818656	7.316976	0.000293264	0.000602449	4.943354	8.056562	3.801717	5.814139	5.214206	8.280926	7.827099	8.061660	1	41	5	89	57	101	2.557891	17.387565	5.970491	1547.960692	1028.620926	1132.794975
373	piR-bta-1005366	ATTGGTGGTTCAGTGGTAGAATTCTCGCCT	2:39839394-39839424:+	-18398.294708	11.845214	4.05108E-09	2.98904E-08	11.655100	0.000000	13.049888	8.216931	13.698480	0.000000	0.000000	0.000000	655	220	1840	0	0	0	1675.418919	93.299129	2197.140851	0.000000	0.000000	0.000000
374	piR-bta-103279	AGTCGGTAGAGCATGAGACTCTTAATC	25:2607801-2607828:+	-38.410431	4.990950	0.026507301	0.037397897	3.479515	0.620709	0.000000	3.183868	7.254677	0.000000	0.000000	1.862126	0	6	21	0	0	1	0.000000	2.544522	25.076064	0.000000	0.000000	11.215792
375	piR-bta-633557	CGGATAGCTCAGTCGGTAGAGCATCAG	23:30029708-30029735:-	-157.276229	7.762583	1.28601E-09	2.13306E-08	8.676629	1.053041	8.399087	8.417932	9.212867	0.000000	3.159123	0.000000	26	253	82	0	2	0	66.505178	107.293999	97.916060	0.000000	36.091962	0.000000
376	piR-bta-3677353	GCCCGGATAGCTCAGTCGGTAGAGCATCAG	23:30029708-30029738:-	-217.939671	9.894220	6.55827E-09	3.65389E-08	10.658360	2.529919	10.602186	9.689850	11.683043	3.900537	3.689221	0.000000	120	612	455	4	3	0	306.946977	259.541214	543.314721	69.571267	54.137943	0.000000
377	piR-bta-7406826	GGGTGGTTCAGTGGTAGAATTCTCGCCTGC	13:21370300-21370330:-	-11165.158341	11.125271	1.01166E-09	2.13306E-08	11.337316	0.000000	12.405439	8.724149	12.882360	0.000000	0.000000	0.000000	419	313	1045	0	0	0	1071.756530	132.739216	1247.832712	0.000000	0.000000	0.000000
378			4:85606086-85606116:-																								
379	piR-bta-6145368	AGCCCGGATAGCTCAGTCGGTAGAGCAT	23:30029711-30029739:-	-102.338273	6.408481	6.97537E-05	0.000165589	6.728703	0.770737	4.749040	7.221772	8.215296	0.000000	2.312210	0.000000	2	110	41	0	1	0	5.115783	46.649565	48.958030	0.000000	18.045981	0.000000
380	piR-bta-127840	GCTCAGTCGGTAGAGCATGAGACTCTTAA	7:40357645-40357674:-	-165.194671	6.953851	2.01672E-08	9.49247E-08	7.752776	0.620709	7.608668	7.011793	8.637867	0.000000	0.000000	1.862126	15	95	55	0	0	1	38.368372	40.288260	65.675406	0.000000	0.000000	11.215792
381	piR-bta-1734759	GACGAGGTGGCCGAGTGGTTAAGGCAATGG	23:31691008-31691038:+	-5.051768	6.570954	0.022344624	0.03202143	7.005660	4.715786	6.038414	6.950223	8.028343	5.015769	5.602801	3.528787	5	91	36	9	12	4	12.789457	38.591913	42.987538	156.535351	216.551774	44.863167
382	piR-bta-11139077	TCGTACCGTGAGTAATAATGCGAG		-524.996950	9.961432	1.06941E-10	1.35803E-08	10.937545	1.172449	10.884955	11.215956	10.711723	3.517347	0.000000	0.000000	146	1764	232	3	0	0	373.452156	748.089382	277.030803	52.178450	0.000000	0.000000
383	piR-bta-653236	GCCCGGATAGCTCAGTCGGTAGAGC	23:30029713-30029738:-	-93.057081	6.280956	0.00016162	0.000351572	6.576765	0.770737	4.749040	6.765960	8.215296	0.000000	2.312210	0.000000	2	80	41	0	1	0	5.115783	33.926956	48.958030	0.000000	18.045981	0.000000
384	piR-bta-10191635	TCGCCGTGATCGTATAGTGGTTAGTACTCTG	10:65234886-65234917:-	-255.050920	8.313009	6.05315E-10	2.13306E-08	9.232425	0.882888	8.652154	9.546387	9.498733	0.000000	0.000000	2.648663	31	554	100	0	0	2	79.294636	234.944171	119.409829	0.000000	0.000000	22.431584
385	piR-bta-470526	TTCTGGGTCGGGGTTTCGTACGTAGCA	16:58623632-58623659:-	4.776839	10.697806	0.03024136	0.041908077	8.748546	11.418719	7.701316	10.195158	8.349165	11.430426	11.427158	11.398573	16	869	45	792	694	1024	40.926264	368.531561	53.734423	13775.110879	12523.910925	11484.970839
386	piR-bta-71218	CTAGCTCAGTCGGTAGAGCATGAGACTC	25:2638936-2638964:+	-224.133289	7.381038	2.2957E-09	2.80152E-08	8.063822	0.620709	7.701316	7.208685	9.281466	0.000000	0.000000	1.862126	16	109	86	0	0	1	40.926264	46.225478	102.692453	0.000000	0.000000	11.215792
387	piR-bta-15033396	TCCGTAGTGTAGTGGTTATCACTTTCGCCT	3:21086764-21086794:-	-144.264638	9.187560	3.89887E-07	1.33886E-06	10.008908	1.488298	10.339337	8.988147	10.699240	0.000000	0.000000	4.464893	100	376	230	0	0	8	255.789148	159.456694	274.642606	0.000000	0.000000	89.726335
388	piR-bta-193855	GCATGGGTGGTTCAGTGGTAGAATTCTCG	13:21370305-21370334:-	-22611.209592	12.142606	3.25974E-09	2.80152E-08	11.969293	0.000000	13.137471	8.658291	14.112118	0.000000	0.000000	0.000000	696	299	2451	0	0	0	1780.292469	126.801998	2926.734905	0.000000	0.000000	0.000000
389	piR-bta-19437729	TTCTCCACTCCCACTGCTTCACTTGACC	4:112179925-112179953:+	-146.491858	6.976889	0.002116199	0.003668079	4.150962	0.770737	0.000000	9.412972	3.039914	0.000000	2.312210	0.000000	0	505	1	0	1	0	0.000000	214.163910	1.194098	0.000000	18.045981	0.000000
390	piR-bta-11071317	AGCCCGGATAGCTCAGTCGGTAGAGCA	23:30029712-30029739:-	-93.076880	6.280956	0.000155794	0.000340254	6.576765	0.770737	4.749040	6.765960	8.215296	0.000000	2.312210	0.000000	2	80	41	0	1	0	5.115783	33.926956	48.958030	0.000000	18.045981	0.000000
391	piR-bta-1278200	GCATGGGTGGTTCAGTGGTAGAATTCTCGCC	13:21370303-21370334:-	-41698.125848	13.025204	2.83072E-09	2.80152E-08	12.781402	0.000000	14.044286	9.312568	14.987352	0.000000	0.000000	0.000000	1305	471	4496	0	0	0	3338.048380	199.744954	5368.665906	0.000000	0.000000	0.000000
392	piR-bta-1647541	CGGCTAGCTCAGTCGGTAGAGCATGAGACT	25:2629355-2629385:+	-765.558814	7.295017	1.24362E-10	1.35803E-08	8.186249	0.000000	7.788371	7.960082	8.810295	0.000000	0.000000	0.000000	17	184	62	0	0	0	43.484155	78.031999	74.034094	0.000000	0.000000	0.000000
393	piR-bta-770770	TCAGTCGGTAGAGCATGAGACTCTTA	25:2627624-2627650:+	-101.099380	6.271874	2.22874E-06	6.43858E-06	7.172743	0.620709	7.403354	6.729774	7.385100	0.000000	0.000000	1.862126	13	78	23	0	0	1	33.252589	33.078782	27.464261	0.000000	0.000000	11.215792
394	piR-bta-1171019	GCCTGGGTAGCTCAGTCGGTAGAGCATCAGAC	23:31230714-31230746:+	-124.238967	7.432884	8.34676E-09	4.34031E-08	8.297399	1.053041	8.091982	7.794778	9.005438	0.000000	3.159123	0.000000	21	164	71	0	2	0	53.715721	69.550260	84.780978	0.000000	36.091962	0.000000
395	piR-bta-4811430	GGCTGGGGGATGTAGCTCAGTGGT	2:39839802-39839826:-	-12.301149	11.457408	0.004336035	0.007109535	11.368262	8.585391	13.029930	8.317886	12.756972	8.545879	7.827099	9.383194	646	236	958	107	57	253	1652.397895	100.084521	1143.946160	1861.031394	1028.620926	2837.595334
396	piR-bta-15495496	GACGAGGTGGCCGAGTGGTTAAGGCTATGGAT		-4.841668	6.523210	0.027628428	0.038879179	6.932177	4.715786	6.038414	6.729774	8.028343	5.015769	5.602801	3.528787	5	78	36	9	12	4	12.789457	33.078782	42.987538	156.535351	216.551774	44.863167
397	piR-bta-1017907	GGCTCGTTGGTCTAGGGGTATGATTCTC	25:2605527-2605555:-	-83.697718	7.219082	1.36674E-07	5.40752E-07	8.015918	1.051608	8.556186	6.934411	8.557156	0.000000	0.000000	3.154825	29	90	52	0	0	3	74.178853	38.167826	62.093111	0.000000	0.000000	33.647376
398	piR-bta-4961108	AGCGTTGGTGGTATAGTGGTGAGCATAGCTGC	11:55712176-55712208:+	-69.047979	6.589538	2.2611E-06	6.49769E-06	7.387039	0.998015	7.027397	6.918424	8.215296	2.994044	0.000000	0.000000	10	89	41	2	0	0	25.578915	37.743739	48.958030	34.785634	0.000000	0.000000
399	piR-bta-1081687	GTCGGGGTTTCGTACGTAGCAGAGC	1:85343019-85343044:+	5.458201	7.811199	0.002108436	0.00366626	5.996514	8.502150	6.708231	6.067104	5.214206	8.734675	8.240548	8.531226	8	49	5	122	76	140	20.463132	20.780261	5.970491	2121.923646	1371.494568	1570.210857
400	piR-bta-1333614	TCTGGGTCGGGGTTTCGTACGTAGC	16:58623633-58623658:-	4.780135	10.669640	0.029826067	0.041437741	8.708735	11.390673	7.608668	10.168371	8.349165	11.399133	11.399887	11.372997	15	853	45	775	681	1006	38.368372	361.746170	53.734423	13479.432994	12289.313170	11283.086586
401	piR-bta-1558318	TCCCTGGTGGTCTAGTGGTTAGGATTCGGCA	4:24092582-24092613:-	-305.504662	14.100744	1.32423E-06	4.01683E-06	14.105914	6.836678	15.259982	11.100257	15.957503	6.672738	6.756139	7.081158	3031	1628	8808	29	27	51	7752.969073	690.413557	10517.617726	504.391686	487.241491	572.005384
402	piR-bta-338997	CTTCTGGGTCGGGGTTTCGTACGTAGCAGA	16:58623630-58623660:-	3.850234	10.765672	0.049054205	0.063468237	9.231016	11.427848	8.399087	10.350683	8.943278	11.443116	11.429235	11.411193	26	968	68	799	695	1033	66.505178	410.516169	81.198684	13896.860597	12541.956906	11585.912965
403	piR-bta-11017480	AGAGCCCGGATAGCTCAGTCGGTAGAG	16:2247966-2247993:+	-293.320329	5.982678	8.12932E-05	0.000189684	6.325836	0.000000	4.749040	6.282261	7.946206	0.000000	0.000000	0.000000	2	57	34	0	0	0	5.115783	24.172956	40.599342	0.000000	0.000000	0.000000
404	piR-bta-10129648	AGCCCGGATAGCTCAGTCGGTAGAGC	23:30029713-30029739:-	-93.098838	6.280956	0.000149548	0.000329247	6.576765	0.770737	4.749040	6.765960	8.215296	0.000000	2.312210	0.000000	2	80	41	0	1	0	5.115783	33.926956	48.958030	0.000000	18.045981	0.000000
405	piR-bta-1293074	CTAGCTCAGTCGGTAGAGCATGAGACTCT	25:2617436-2617465:+	-226.191156	7.393442	2.61513E-09	2.80152E-08	8.073735	0.620709	7.701316	7.221772	9.298119	0.000000	0.000000	1.862126	16	110	87	0	0	1	40.926264	46.649565	103.886551	0.000000	0.000000	11.215792
406	piR-bta-2510629	TGTGGGTTCGAGTCCCATCTGGGTCGCCA		-23.162757	7.757879	1.74375E-06	5.1744E-06	8.442439	4.141235	7.288587	8.724149	9.314581	4.452933	3.689221	4.281551	12	313	88	6	3	7	30.694698	132.739216	105.080649	104.356901	54.137943	78.510543
407	piR-bta-466012	TTGGTGGTTCAGTGGTAGAATTCTCG	3:21085366-21085392:+	-4952.324189	9.954523	5.90245E-09	3.46531E-08	10.010483	0.000000	11.095906	7.113595	11.821947	0.000000	0.000000	0.000000	169	102	501	0	0	0	432.283660	43.256869	598.243243	0.000000	0.000000	0.000000
408	piR-bta-2018590	TGAGGGTTCGAGTCCCTTCGTGGTCGCC	19:56412030-56412058:+	-21.481155	7.301438	1.08464E-05	2.87483E-05	8.132558	2.915594	7.788371	8.663097	7.946206	3.900537	4.846244	0.000000	17	300	34	4	7	0	43.484155	127.226085	40.599342	69.571267	126.321868	0.000000
409	piR-bta-905827	GGTTCAGTGGTAGAATTCTCGCCTG	18:1423814-1423839:+	-6631.234752	10.374792	1.37627E-09	2.17583E-08	10.640792	0.000000	11.601722	8.163675	12.156978	0.000000	0.000000	0.000000	240	212	632	0	0	0	613.893955	89.906434	754.670118	0.000000	0.000000	0.000000
410	piR-bta-11097439	TCCACTCCCACTGCTTCACTTGACCAGC	4:112179928-112179956:+	-237.773601	7.583746	4.10296E-06	1.13716E-05	7.390235	0.770737	4.749040	9.754314	7.667351	0.000000	2.312210	0.000000	2	640	28	0	1	0	5.115783	271.415649	33.434752	0.000000	18.045981	0.000000
411	piR-bta-1571072	TTCAGTGGTAGAATTCTCGCCTGCCA	5:23826391-23826417:+	-1812.290220	8.511630	3.28895E-09	2.80152E-08	9.064639	0.000000	9.981197	7.208685	10.004034	0.000000	0.000000	0.000000	78	109	142	0	0	0	199.515535	46.225478	169.561957	0.000000	0.000000	0.000000
412	piR-bta-1060819	GCCCGGCTAGCTCAGTCGGTAGAGCATGGGA	10:70547107-70547138:-	-32.448661	6.541958	0.000694898	0.001340663	6.953550	1.358674	5.316007	7.195479	8.349165	0.000000	4.076023	0.000000	3	108	45	0	4	0	7.673674	45.801391	53.734423	0.000000	72.183925	0.000000
413	piR-bta-7732821	AATTCTCGCCTGCCACGCGGGAGGCCCGG	3:8034316-8034345:+	-98.727354	4.634668	0.001958522	0.003449526	5.254222	0.000000	4.749040	5.325139	5.688486	0.000000	0.000000	0.000000	2	29	7	0	0	0	5.115783	12.298522	8.358688	0.000000	0.000000	0.000000
414	piR-bta-2358954	CCGCCTGGGAATACCGGGTGCTGTAGGCTTA	21:33002888-33002919:+	-15.141113	10.035305	0.001246095	0.002283114	10.240598	6.678889	9.529211	12.167007	9.025577	5.993298	8.037737	6.005632	57	3411	72	18	66	24	145.799814	1446.560591	85.975077	313.070702	1191.034757	269.179004
415			26:5302461-5302492:-																								
416	piR-bta-18390009	AGAGCCCGGATAGCTCAGTCGGTAG	16:2247966-2247991:+	-258.993919	5.808726	0.000272471	0.000565662	5.990348	0.000000	4.749040	5.275798	7.946206	0.000000	0.000000	0.000000	2	28	34	0	0	0	5.115783	11.874435	40.599342	0.000000	0.000000	0.000000
417	piR-bta-218493	GGTGGTTCAGTGGTAGAATTCTCGCC	3:21210209-21210235:+	-10805.043624	11.077906	4.29747E-09	3.01423E-08	11.026402	0.000000	12.374119	7.846388	12.858700	0.000000	0.000000	0.000000	410	170	1028	0	0	0	1048.735506	72.094782	1227.533041	0.000000	0.000000	0.000000
418	piR-bta-1175506	TGGGTCGGGGTTTCGTACGTAGCAG	16:58623631-58623656:-	4.363186	10.718001	0.034207462	0.046927825	9.060490	11.415236	8.399087	10.144517	8.637867	11.439502	11.406227	11.399981	26	839	55	797	684	1025	66.505178	355.808952	65.675406	13862.074963	12343.451113	11496.186631
419	piR-bta-777042	GGCATTGGTGGTTCAGTGGTAGAATTCTCGC	18:1819424-1819455:+	-44538.100143	13.120239	3.72373E-09	2.89988E-08	12.847748	0.000000	14.111246	9.333819	15.098178	0.000000	0.000000	0.000000	1367	478	4855	0	0	0	3496.637652	202.713563	5797.347191	0.000000	0.000000	0.000000
420	piR-bta-11300205	CGGGAGGCCCGGGTTCGATTCCCGG	NKLS02000508.1:23523-23548	-6.314000	7.772582	0.000909669	0.001706801	8.401490	5.869064	8.556186	7.526026	9.122259	5.993298	6.096544	5.517349	29	136	77	18	17	17	74.178853	57.675825	91.945568	313.070702	306.781680	190.668461
421	piR-bta-182313	GGGGGATGTAGCTCAGTGGTAGAGCGCG	2:39839794-39839822:-	-10.949015	11.532654	0.004172996	0.006862819	11.653898	8.851134	13.045477	9.084426	12.831790	8.758079	8.240548	9.554776	653	402	1009	124	76	285	1670.303136	170.482954	1204.845173	2156.709279	1371.494568	3196.500673
422	piR-bta-6139736	CCGGATAGCTCAGTCGGTAGAGCATCAG	23:30029708-30029736:-	-174.714339	7.915849	3.30976E-09	2.80152E-08	8.863056	1.053041	8.652154	8.724149	9.212867	0.000000	3.159123	0.000000	31	313	82	0	2	0	79.294636	132.739216	97.916060	0.000000	36.091962	0.000000

423	piR-bta-5827742	GACCTGCTTCTGGGTCGGGGTTTCGTACGT	16:58623636-58623666:-	7.356126	9.599666	0.016687093	0.024558363	5.883910	10.405646	4.749040	8.953264	3.949426	10.412630	10.419349	10.384960	2	367	2	391	345	507	5.115783	155.639911	2.388197	6800.591356	6225.863500	5686.406460
424	piR-bta-1498695	TTCTGGGTCGGGGTTTCGTACGTAGCAGAG	16:58623629-58623659:-	3.866023	10.764492	0.0489502	0.063468237	9.236414	11.427848	8.399087	10.325141	8.985014	11.443116	11.429235	11.411193	26	951	70	799	695	1033	66.505178	403.306691	83.586880	13896.860597	12541.956906	11585.912965
425	piR-bta-4631092	AGCCCGGCTAGCTCAGTCGGTAGAGCATGAGA	3:45753111-45753143:+	-76.233490	7.675955	9.80752E-07	3.07753E-06	8.388666	1.358674	7.870470	7.768264	9.527263	0.000000	4.076023	0.000000	18	161	102	0	4	0	46.042047	68.277999	121.798025	0.000000	72.183925	0.000000
426	piR-bta-861227	CTCAGTCGGTAGAGCATCAGACTTT	19:27761059-27761084:+	-70.495218	6.658601	4.05576E-06	1.13485E-05	7.525102	1.053041	8.021857	6.835718	7.717732	0.000000	3.159123	0.000000	20	84	29	0	2	0	51.157830	35.623304	34.628850	0.000000	36.091962	0.000000
427	piR-bta-8491508	AGCTCAGTCGGTAGAGCATCAGAC	23:30158666-30158690:+	-90.740944	7.003032	5.2899E-07	1.76115E-06	7.838439	1.053041	8.091982	6.981337	8.441998	0.000000	3.159123	0.000000	21	93	48	0	2	0	53.715721	39.440086	57.316718	0.000000	36.091962	0.000000
428	piR-bta-623346	GCTTCTGGGTCGGGGTTTCGTACGTAGCAG	16:58623631-58623661:-	3.821059	10.767908	0.049310884	0.063499393	9.236936	11.427848	8.399087	10.368444	8.943278	11.443116	11.429235	11.411193	26	980	68	799	695	1033	66.505178	415.605212	81.198684	13896.860597	12541.956906	11585.912965
429	piR-bta-7647581	AGCGCCGCTGGTGTAGTGGTATCATGCAAG	11:68555304-68555334:-	-33.364459	17.006564	0.000857794	0.001626234	16.849290	12.808027	17.931134	13.673552	18.943183	12.051185	13.035138	13.337757	19306	9693	69769	1218	2116	3928	49382.652892	4110.674820	83311.043499	21184.450822	38185.296135	44055.630328
430	piR-bta-3399442	AGCTCAGTCGGTAGAGCATCAGACTTTT	23:31557025-31557053:+	-93.805731	7.045321	1.38345E-07	5.41136E-07	7.914222	1.053041	8.091982	7.208685	8.441998	0.000000	3.159123	0.000000	21	109	48	0	2	0	53.715721	46.225478	57.316718	0.000000	36.091962	0.000000
431	piR-bta-6987700	TGAGGTAGTAGGTTGTGTGGTTGG		-16.490991	9.699904	3.70471E-05	9.15282E-05	10.386413	6.495222	10.105068	11.444555	9.609617	5.826201	6.999164	6.660300	85	2067	108	16	32	38	217.420776	876.587729	128.962615	278.285068	577.471397	426.200090
432	piR-bta-9778002	AGCCAAGGGAACGGGCTTGGCAGA	14:16684346-16684370:+	-5.939913	8.361562	0.006017545	0.009745695	8.811988	6.524204	9.554268	7.285482	9.596214	6.672738	6.096544	6.803331	58	115	107	29	17	42	148.357706	48.769999	127.768517	504.391686	306.781680	471.063257
433	piR-bta-9634894	GAACGGGCTTGGCAGAATCAGCGGG	14:16684354-16684379:+	-4.670030	12.340106	0.03818082	0.05134662	12.883395	10.836008	13.509874	11.797442	13.342868	10.804169	10.849711	10.854145	901	2640	1438	513	465	702	2304.660222	1119.589552	1717.113339	8922.515001	8391.381239	7873.485868
434	piR-bta-8741278	ACTCACCTGCCGAATCAACTAGCCCCG	NKLS02002111.1:9238-9265:-	-26.667002	10.156378	0.000187528	0.000399962	10.211331	6.232778	9.123221	9.091573	12.419199	5.297145	6.858311	6.542878	43	404	758	11	29	35	109.989334	171.331128	905.126503	191.320984	523.333454	392.552714
435	piR-bta-7324107	AGCCAAGGGAACGGGCTTGGCAGAA	14:16684346-16684371:+	-5.939945	8.361562	0.006033049	0.009745695	8.811988	6.524204	9.554268	7.285482	9.596214	6.672738	6.096544	6.803331	58	115	107	29	17	42	148.357706	48.769999	127.768517	504.391686	306.781680	471.063257
436	piR-bta-755639	AACGGGCTTGGCAGAATCAGCGGG	14:16684355-16684379:+	-4.694934	12.312252	0.036068274	0.048906045	12.858397	10.801756	13.461024	11.774312	13.339856	10.767159	10.808826	10.829284	871	2598	1435	500	452	690	2227.923478	1101.777900	1713.531044	8696.408383	8156.783484	7738.896366
437	piR-bta-11397238	CCAAGTGTTGGATTGTTCACCCACC	NKLS02002111.1:7614-7639:-	-19.674111	5.730742	0.001054362	0.001958101	6.316526	2.375169	5.316007	6.378892	7.254677	2.164632	2.312210	2.648663	3	61	21	1	1	2	7.673674	25.869304	25.076064	17.392817	18.045981	22.431584
438	piR-bta-16735312	GGTGGGGAGTTTGGCTGGGGCGGCA	NKLS02002111.1:7920-7945:-	5.047877	9.849591	0.008043097	0.012692286	8.136373	10.570413	8.826831	8.197190	7.385100	10.714295	10.398303	10.598642	35	217	23	482	340	588	89.526202	92.026868	27.464261	8383.337682	6135.633594	6594.885599
439	piR-bta-4705990	GGTGGGGAGTTTGGCTGGGGCGGCAC	NKLS02002111.1:7919-7945:-	3.293566	10.030626	0.045400778	0.059445623	8.904989	10.633661	9.019079	9.084426	8.611462	10.775784	10.480705	10.644493	40	402	54	503	360	607	102.315659	170.482954	64.481308	8748.586834	6496.553218	6807.985644
440	piR-bta-1619880	GGAACGGGCTTGGCAGAATCAGCGGG	14:16684353-16684379:+	-4.528207	12.370007	0.042494811	0.056399496	12.910213	10.902267	13.533692	11.842089	13.354856	10.859315	10.936946	10.910541	916	2723	1450	533	494	730	2343.028595	1154.788769	1731.442518	9270.371337	8914.714693	8187.528039
441	piR-bta-6583948	TCTCACACAGAAATCGCACCCGTCT	21:65057705-65057730:+	-35.751232	9.099186	6.82123E-05	0.000162637	9.039096	4.861428	7.701316	11.387629	8.028343	5.297145	4.380741	4.906397	16	1987	36	11	5	11	40.926264	842.660772	42.987538	191.320984	90.229906	123.373710
442	piR-bta-18501426	GTTGGTCTAGGGGTATGATTCTCGG	15:55651416-55651441:-	-353.755790	6.207180	2.35942E-05	6.01983E-05	6.778557	0.000000	7.509660	5.059599	7.766413	0.000000	0.000000	0.000000	14	24	30	0	0	0	35.810481	10.178087	35.822949	0.000000	0.000000	0.000000
443	piR-bta-423474	GCCCGGATGATCCTCAGTGGTCTGGGGTGCAGGC	18:53083919-53083953:-	-12.553794	5.270364	0.007584217	0.012072835	5.918115	1.850449	5.721963	5.463780	6.568601	0.000000	3.689221	1.862126	4	32	13	0	3	1	10.231566	13.570782	15.523278	0.000000	54.137943	11.215792
