# Supplementary material for: Exosomal small non-coding RNA profiling and the role of PIWI-interacting RNA pathway genes in Lumpy skin disease virus-infected bovines
Source: Anim Biosci. 2025 Jun 4;38(11):2364–76. doi: 10.5713/ab.25.0217 (PMC12580968; doi:10.5713/ab.25.0217)
Supplement: Supplementary file 2 [file ab-25-0217-supplementary-2.pdf]

Supplement 2. Gene ontology enrichment analysis

Sublist	Category	Term	Fold Enrichm	P-Value	Count	%
1	GOTERM_MF_DIRECT	protein binding	1.9	4.8E-5	33	11.2
2	GOTERM_MF_DIRECT	oxidoreductase activity	5.8	3.9E-5	6	2.0
3	GOTERM_MF_DIRECT	transcription factor activity, sequence-specific DNA binding	3.5	4.6E-5	9	3.1
4	GOTERM_MF_DIRECT	methenyltetrahydrofolate cyclohydrolase activity	87.0	2.3E-5	2	0.7
5	GOTERM_MF_DIRECT	methylenetetrahydrofolate dehydrogenase (NAD+) activity	87.0	2.3E-4	2	0.7
6	GOTERM_MF_DIRECT	methylenetetrahydrofolate dehydrogenase (NADP+) activity	65.2	3.0E-4	2	0.7
7	GOTERM_MF_DIRECT	RNA polymerase II transcription factor activity, sequence-specific DNA binding	1.8	3.3E-4	15	5.1
8	GOTERM_MF_DIRECT	RNA polymerase II core promoter proximal region sequence-specific DNA binding	1.8	4.6E-4	15	5.1
9	GOTERM_MF_DIRECT	tRNA binding	5.8	9.5E-4	3	1.0
10	GOTERM_MF_DIRECT	intracellular calcium activated chloride channel activity	17.4	1.1E-3	2	0.7
11	GOTERM_MF_DIRECT	metal ion binding	1.4	1.1E-3	19	6.5
12	GOTERM_MF_DIRECT	identical protein binding	1.7	1.3E-3	11	3.7
13	GOTERM_MF_DIRECT	C-C chemokine binding	12.4	1.5E-3	2	0.7
14	GOTERM_MF_DIRECT	iron-sulfur cluster binding	12.4	1.5E-3	2	0.7
15	GOTERM_MF_DIRECT	C-C chemokine receptor activity	10.4	1.7E-3	2	0.7
16	GOTERM_MF_DIRECT	actin filament binding	2.6	2.0E-2	4	1.4
17	GOTERM_MF_DIRECT	Wnt-protein binding	7.9	2.2E-2	2	0.7
18	GOTERM_MF_DIRECT	small GTPase binding	2.4	2.2E-2	4	1.4
19	GOTERM_MF_DIRECT	DNA binding	1.5	2.4E-2	10	3.4
20	GOTERM_MF_DIRECT	ATP binding	1.3	2.4E-2	15	5.1
21	GOTERM_MF_DIRECT	phosphotyrosine binding	7.1	2.5E-2	2	0.7
22	GOTERM_MF_DIRECT	pheromone receptor activity	6.4	2.7E-2	2	0.7
23	GOTERM_MF_DIRECT	promoter-specific chromatin binding	6.2	2.7E-2	2	0.7
24	GOTERM_MF_DIRECT	calcium channel activity	5.7	3.0E-2	2	0.7
25	GOTERM_MF_DIRECT	nucleotide binding	4.6	3.5E-2	2	0.7
26	GOTERM_MF_DIRECT	flavin adenine dinucleotide binding	4.2	3.8E-2	2	0.7
27	GOTERM_MF_DIRECT	protein dimerization activity	2.3	3.8E-2	3	1.0
28	GOTERM_MF_DIRECT	transcription coactivator activity	2.1	4.2E-2	3	1.0
29	GOTERM_MF_DIRECT	protein serine/threonine kinase activity	1.7	4.2E-2	4	1.4

30	GOTERM_MF_DIRECT	protein kinase activity	2.0	4.4E-2	3	1.0
31	GOTERM_MF_DIRECT	sequence-specific DNA binding	1.9	4.6E-2	3	1.0
32	GOTERM_MF_DIRECT	phospholipid binding	3.2	4.6E-2	2	0.7
