# Supplementary material for: Exosomal small non-coding RNA profiling and the role of PIWI-interacting RNA pathway genes in Lumpy skin disease virus-infected bovines
Source: Anim Biosci. 2025 Jun 4;38(11):2364–76. doi: 10.5713/ab.25.0217 (PMC12580968; doi:10.5713/ab.25.0217)
Supplement: Supplementary file 3 [file ab-25-0217-supplementary-3.pdf]

**Supplement 3.** KEGG pathway enrichment analysis

| Sublist | Category | Term | Fold Enrichment | P-Value | Count | % | P-Value | Fold Enrichment |
|---|---|---|---|---|---|---|---|---|
| 1 | KEGG_PATHWAY | Chemical carcinogenesis - receptor activation | 4.6 | 3.1E-10 | 10 | 3.4 | 3.1E-10 | 4.6 |
| 2 | KEGG_PATHWAY | Biosynthesis of cofactors | 3.3 | 6.4E-10 | 5 | 1.7 | 6.4E-10 | 3.3 |
| 3 | KEGG_PATHWAY | Fatty acid metabolism | 5.1 | 1.2E-10 | 3 | 1.0 | 1.2E-10 | 5.1 |
| 4 | KEGG_PATHWAY | Notch signaling pathway | 4.9 | 1.2E-9 | 3 | 1.0 | 1.2E-9 | 4.9 |
| 5 | KEGG_PATHWAY | Aminoacyl-tRNA biosynthesis | 4.7 | 1.3E-9 | 3 | 1.0 | 1.3E-9 | 4.7 |
| 6 | KEGG_PATHWAY | One carbon pool by folate | 10.9 | 1.7E-8 | 2 | 0.7 | 1.7E-8 | 10.9 |
| 7 | KEGG_PATHWAY | Fatty acid biosynthesis | 10.9 | 1.7E-8 | 2 | 0.7 | 1.7E-8 | 10.9 |
| 8 | KEGG_PATHWAY | Drug metabolism - other enzymes | 3.9 | 1.8E-7 | 3 | 1.0 | 1.8E-7 | 3.9 |
| 9 | KEGG_PATHWAY | Complement and coagulation cascades | 3.2 | 2.4E-7 | 3 | 1.0 | 2.4E-7 | 3.2 |
| 10 | KEGG_PATHWAY | Metabolic pathways | 1.2 | 2.4E-6 | 20 | 6.8 | 2.4E-6 | 1.2 |
| 11 | KEGG_PATHWAY | Glycosphingolipid biosynthesis - lacto and neolacto series | 6.8 | 2.5E-6 | 2 | 0.7 | 2.5E-6 | 6.8 |
| 12 | KEGG_PATHWAY | Propanoate metabolism | 6.3 | 2.7E-6 | 2 | 0.7 | 2.7E-6 | 6.3 |
| 13 | KEGG_PATHWAY | Th1 and Th2 cell differentiation | 2.0 | 6.3E-5 | 2 | 0.7 | 6.3E-5 | 2.0 |
| 14 | KEGG_PATHWAY | cGMP-PKG signaling pathway | 4.3 | 8.2E-5 | 2 | 0.7 | 8.2E-5 | 1.2 |
| 15 | KEGG_PATHWAY | Nicotinate and nicotinamide metabolism | 5.2 | 3.2E-5 | 2 | 0.7 | 3.2E-5 | 5.2 |
| 16 | KEGG_PATHWAY | Thyroid hormone signaling pathway | 2.4 | 3.4E-4 | 3 | 1.0 | 3.4E-4 | 2.4 |
| 17 | KEGG_PATHWAY | Platelet activation | 2.4 | 3.5E-4 | 3 | 1.0 | 3.5E-4 | 2.4 |
| 18 | KEGG_PATHWAY | AMPK signaling pathway | 2.4 | 3.5E-4 | 3 | 1.0 | 3.5E-4 | 2.4 |
| 19 | KEGG_PATHWAY | cGMP-PKG signaling pathway | 2.8 | 8.2E-4 | 2 | 0.7 | 8.2E-4 | 1.2 |
| 20 | KEGG_PATHWAY | Cytokine-cytokine receptor interaction | 3.4 | 9.7E-4 | 2 | 0.7 | 9.7E-4 | 0.6 |
| 21 | KEGG_PATHWAY | Valine, leucine and isoleucine degradation | 3.9 | 4.0E-4 | 2 | 0.7 | 4.0E-4 | 3.9 |
| 22 | KEGG_PATHWAY | Pyrimidine metabolism | 3.5 | 4.3E-4 | 2 | 0.7 | 4.3E-4 | 3.5 |
| 23 | KEGG_PATHWAY | Regulation of lipolysis in adipocytes | 3.3 | 4.5E-4 | 2 | 0.7 | 4.5E-4 | 3.3 |
| 24 | KEGG_PATHWAY | Calcium signaling pathway | 1.5 | 4.9E-4 | 4 | 1.4 | 4.9E-4 | 1.5 |
| 25 | KEGG_PATHWAY | Renin secretion | 2.7 | 5.2E-3 | 2 | 0.7 | 5.2E-3 | 2.7 |
| 26 | KEGG_PATHWAY | Platinum drug resistance | 2.5 | 5.5E-3 | 2 | 0.7 | 5.5E-3 | 2.5 |
| 27 | KEGG_PATHWAY | Synaptic vesicle cycle | 2.5 | 5.5E-3 | 2 | 0.7 | 5.5E-3 | 2.5 |
| 28 | KEGG_PATHWAY | Polycomb repressive complex | 2.4 | 5.7E-3 | 2 | 0.7 | 5.7E-3 | 2.4 |
| 29 | KEGG_PATHWAY | Arrhythmogenic right ventricular cardiomyopathy | 2.4 | 5.7E-3 | 2 | 0.7 | 5.7E-3 | 2.4 |

| | | | | | | | | |
|---|---|---|---|---|---|---|---|---|
| 30 | KEGG_PATHWAY | ECM-receptor interaction | 2.2 | 6.0E-3 | 2 | 0.7 | 6.0E-3 | 2.2 |
| 31 | KEGG_PATHWAY | Gap junction | 2.2 | 6.0E-3 | 2 | 0.7 | 6.0E-3 | 2.2 |
| 32 | KEGG_PATHWAY | Focal adhesion | 1.5 | 6.3E-3 | 3 | 1.0 | 6.3E-3 | 1.5 |
| 33 | KEGG_PATHWAY | Ribosome biogenesis in eukaryotes | 2.1 | 6.2E-3 | 2 | 0.7 | 6.2E-3 | 2.1 |
| 34 | KEGG_PATHWAY | Salmonella infection | 1.1 | 7.4E-3 | 3 | 1.0 | 7.4E-3 | 1.1 |
| 35 | KEGG_PATHWAY | Signaling pathways regulating pluripotency of stem cells | 1.4 | 7.7E-3 | 2 | 0.7 | 7.7E-3 | 1.4 |
| 36 | KEGG_PATHWAY | Fluid shear stress and atherosclerosis | 1.4 | 7.7E-3 | 2 | 0.7 | 7.7E-3 | 1.4 |
| 37 | KEGG_PATHWAY | Gastric cancer | 1.3 | 7.9E-3 | 2 | 0.7 | 7.9E-3 | 1.3 |
| 38 | KEGG_PATHWAY | Oxytocin signaling pathway | 1.3 | 7.9E-2 | 2 | 0.7 | 7.9E-2 | 1.3 |
| 39 | KEGG_PATHWAY | Hippo signaling pathway | 1.3 | 8.0E-2 | 2 | 0.7 | 8.0E-2 | 1.3 |
| 40 | KEGG_PATHWAY | Cellular senescence | 1.2 | 8.0E-2 | 2 | 0.7 | 8.0E-2 | 1.2 |
| 41 | KEGG_PATHWAY | Efferocytosis | 1.2 | 8.2E-2 | 2 | 0.7 | 8.2E-2 | 1.2 |
| 42 | KEGG_PATHWAY | Phagosome | 1.2 | 8.2E-2 | 2 | 0.7 | 8.2E-2 | 1.2 |
| 43 | KEGG_PATHWAY | Ribosome | 1.2 | 8.2E-2 | 2 | 0.7 | 8.2E-2 | 1.2 |
| 44 | KEGG_PATHWAY | Hepatocellular carcinoma | 1.1 | 8.3E-2 | 2 | 0.7 | 8.3E-2 | 1.1 |
| 45 | KEGG_PATHWAY | Axon guidance | 1.1 | 8.4E-2 | 2 | 0.7 | 8.4E-2 | 1.1 |
| 46 | KEGG_PATHWAY | Proteoglycans in cancer | 1.0 | 8.8E-2 | 2 | 0.7 | 8.8E-2 | 1.0 |
| 47 | KEGG_PATHWAY | JAK-STAT signaling pathway | 0.9 | 8.9E-2 | 2 | 0.7 | 8.9E-2 | 0.9 |
| 48 | KEGG_PATHWAY | Regulation of actin cytoskeleton | 0.9 | 9.0E-2 | 2 | 0.7 | 9.0E-2 | 0.9 |
| 49 | KEGG_PATHWAY | Epstein-Barr virus infection | 0.9 | 9.0E-2 | 2 | 0.7 | 9.0E-2 | 0.9 |
| 50 | KEGG_PATHWAY | cAMP signaling pathway | 0.8 | 9.2E-2 | 2 | 0.7 | 9.2E-2 | 0.8 |
| 51 | KEGG_PATHWAY | Amyotrophic lateral sclerosis | 0.5 | 9.8E-2 | 2 | 0.7 | 9.8E-2 | 0.5 |
| 52 | KEGG_PATHWAY | PI3K-Akt signaling pathway | 0.5 | 9.8E-2 | 2 | 0.7 | 9.8E-2 | 0.5 |
| 53 | KEGG_PATHWAY | Herpes simplex virus 1 infection | 0.5 | 9.8E-2 | 2 | 0.7 | 9.8E-2 | 0.5 |
| 54 | KEGG_PATHWAY | Pathways of neurodegeneration - multiple diseases | 0.4 | 9.9E-2 | 2 | 0.7 | 9.90E-02 | 0.4 |
| 55 | KEGG_PATHWAY | Olfactory transduction | 0.2 | 2.0E-2 | 2 | 0.7 | 2.00E-02 | 0.2 |
